# Prediction of hospitalisation in young children with pneumonia in Malawi: A machine learning-based approach

**DOI:** 10.1371/journal.pmed.1005122

**Published:** 2026-06-09

**Authors:** Patrick Staunton, Mohammad Adib Makrooni, Master Chisale, Billy Nyambolo, Joseph Wu, Damien McCarthy, Mark Ledwidge, Yasir Bin Nisar, Chris Watson, Balwani Mbakaya, Cathal Seoighe, Joe Gallagher

**Affiliations:** 1 School of Mathematical and Statistical Sciences, University of Galway, Galway, Ireland; 2 Department of Biological Sciences, Faculty of Science, Technology and Innovation, Mzuzu University, Mzuzu, Malawi; 3 Ministry of Health, Lilongwe, Malawi; 4 Luke International, Oslo, Norway; 5 School of Medicine, University College Dublin, Dublin, Ireland; 6 World Health Organisation, Geneva, Switzerland; 7 Wellcome-Wolfson Institute for Experimental Medicine, Queen’s University, Belfast, United Kingdom; 8 Department of Public Health, University of Livingstonia, Livingstonia, Malawi; Washington University School of Medicine, UNITED STATES OF AMERICA

## Abstract

**Background:**

Globally, pneumonia remains the single biggest cause of mortality in children under 5 years of age. This study sought to train and test a prediction model for hospitalisation within 7 days after initial presentation in 2- to 59-month-old Malawian children with WHO-defined pneumonia in primary care and compare its performance to existing risk prediction models.

**Methods and findings:**

BIOTOPE is a cohort study of children with pneumonia in a primary healthcare setting in Malawi. The training cohort involved nine primary care centres and the testing cohort involved two primary care centres in Northern Malawi. The training cohort was recruited between December 2022 and April 2023 while the testing cohort was recruited in 2016. Participants were consecutive children aged 2–59 months presenting with cough and/or difficulty breathing and who were diagnosed as WHO-defined pneumonia in primary care of any severity. The training cohort was used to train and validate a machine learning model with a prespecified primary outcome defined as hospitalisation and/or death within 7 days as the outcome. This model was then further evaluated in the testing cohort.

Median age was 15 months (interquartile range 8−27) in the training and 17 months (interquartile range 9−29) in the external testing cohort (52.1% and 54.4% male, respectively). Hospitalisation occurred in 14.3% (294) of the training cohort and 12.1% (55) of the testing cohort. There was one death in the training cohort only. WHO danger signs were present in 17.6% (360) and 15.9% (70) of children in the training and testing cohorts, respectively. The optimal machine learning model achieved an area under the receiver operating characteristic and precision recall curves of 0.87 and 0.57, respectively, in the testing cohort outperforming existing risk prediction models; furthermore, this model produced an expected calibration error of 0.16 (a logistic regression model using severity status as the response variable and the log odds of the machine learning model’s calibrated probabilities produced an intercept estimate of −0.32 and a slope estimate of 1.13). Key limitations include the use of hospitalisation and/or death as a severity outcome, which may reflect health system factors rather than true disease severity, that mortality-based comparisons were not possible due to low mortality in these primary care cohorts, and that comparator tools were developed for hospital populations rather than primary care populations.

**Conclusion:**

This machine learning score outperformed traditional pneumonia risk scores in predicting hospitalisation within 7 days in Malawian children presenting to primary care. Traditional pneumonia risk scores diminish in performance when externally applied to new datasets suggesting they may not generalise well beyond their original derivation settings. Mortality-related findings are not applicable as there was only one death in this cohort. Overall these findings support the potential of machine learning to meaningfully improve early identification of children at risk of severe pneumonia in low-resource primary care settings. Further external validation and clinical impact studies are needed to confirm these results.

## Author summary

**Why was this study done?:**

- Pneumonia remains the leading cause of death among children under five worldwide, especially in low-resource settings.
- Health workers in primary care often rely on pre-defined severity signs to decide which children need hospital care. However, these signs can miss some severely ill children, and existing risk prediction scores—developed mainly for hospitalised patients—perform poorly in community settings.
- This study aimed to create and test a new tool using machine learning to better identify young children with pneumonia who are at risk of hospitalisation soon after presentation in community settings.

**What did the researchers do and find?:**

- Researchers trained a machine learning model using data from 2,053 Malawian children aged 2–59 months with WHO-defined pneumonia who attended nine primary care centres between December 2022 and April 2023. Multiple classifier types were evaluated using cross-validation, with a random forest model trained on clinical features—including temperature, weight-for-age, chest indrawing, heart rate, and respiratory rate—selected as the best performer. The model was then tested on an independent dataset of 456 children collected in 2016.
- The model was used to predict which children would be hospitalised within seven days. The best-performing algorithm—a random forest model—achieved strong predictive performance (Area Under the Curve [AUC] = 0.87 in the testing group), outperforming existing WHO and research-based pneumonia severity scores.

**What do these findings mean?:**

- This study shows that a machine learning–based approach may more accurately identify children at risk of poor outcomes from pneumonia in primary care settings than current risk scores.
- If further validated, this type of model could be integrated into existing community health information systems, supporting frontline health workers in making timely hospital referral decisions.
- However, further testing in larger and more diverse populations is needed, and the findings should be interpreted with caution until clinical impact studies showing how it affects outcomes in children are undertaken

## Abstract 3

In a machine learning study, Patrick Staunton and colleagues develop and test a model to predict hospitalisation in children with pneumonia under 5 years of age in Malawi.

## Introduction

Despite recent advances in reducing childhood mortality, pneumonia remains a major cause of morbidity and mortality among children under five years of age [1]. In primary care, the focus is on identifying those children at risk of severe illness, providing antibiotics and ensuring they are referred to hospital appropriately for further management. The World Health Organisation (WHO) has identified severity criteria to guide referral to hospital and these criteria have been implemented in initiatives such as Integrated Management of Childhood Illness (IMCI) [2,3] and integrated Community Case Management (iCCM) [4].

However, recent studies have highlighted concerns regarding changing markers of severity, which may mean children with severe illness may not be appropriately identified [5]. Clinical prediction rules are tools that combine features derived from patient history, examination and basic investigations to guide clinicians by providing them with a likelihood of a target diagnosis or outcome [6]. Clinical prediction rules can serve to reassure clinicians in their decision to adopt a “watch and wait” approach and to provide a guide as to who is at risk of severe disease, requiring hospital admission, more intensive care and other interventions. Machine learning based clinical prediction rules have recently been shown to be of benefit in this regard [7–9]. The BIOTOPE study (BIOmarkers TO diagnose PnEumonia) involves two cohorts from 2016 [10] and 2022/23. We trained and tested a clinical prediction model for use by primary healthcare workers based on machine learning (ML) techniques in these cohorts. The aim was to identify children at risk of hospitalisation with WHO-defined pneumonia [11] who presented in primary healthcare facilities. We compared the performance of the model to WHO markers of severity and four other risk scores: Respiratory Index of Severity in Children (RISC) [12] for HIV–negative children, the Pneumonia Etiology Research for Child Health (PERCH) [13], the Pneumonia Research Partnership to Assess WHO Recommendations (PREPARE) [14] as well as a variant of the RISC score designed specifically for use in a Malawian context (RISC Malawi) [14]. It is important to note that risk models explored for comparative purposes involved hospitalised patients and used mortality alone as an outcome measure, implying a sicker cohort than BIOTOPE, which involved primary care patients only. However, it is in primary care where most patients initially present highlighting the need for risk scores that perform well in this setting. We hypothesised that a machine learning-based clinical prediction model, trained and tested in a primary care cohort of Malawian children with WHO-defined pneumonia, would outperform existing risk prediction tools in identifying those at risk of hospitalisation within seven days of initial presentation.

## Methods

### Study population

The BIOTOPE training cohort recruited children aged 2–59 months with WHO defined pneumonia from nine primary care facilities in Northern Malawi (Table A in S1 Appendix). Children presenting with cough and/or difficulty breathing to these facilities were assessed for study enrolment; children aged 2–59 months were deemed eligible to participate in the study if the main presenting complaint was cough or difficulty breathing associated with fast breathing (defined as ≥50 breaths per minute if aged 2–11 months or ≥40 breaths per minute if aged 12–59 months) and/or chest in-drawing including those with general danger signs. The study details were discussed with a parent/guardian of any patient who was eligible for our inclusion criteria, who in turn provided written informed consent. Under the Malawi Child Care, Protection and Justice Act, a guardian is an adult legally responsible for a child’s welfare, upbringing, and property. Details on study training and monitoring are in the supplemental file (e9 BIOTOPE study training schedule in S1 Appendix). Ethical approval was obtained from National Health Sciences Research Ethics Committee (NHSRC) from the Malawi Ministry of Health (Number 4469). In order to assess performance on unseen data and assess dataset shift, we utilised a separate dataset collected in accordance with the BIOTOPE framework in 2016 (BIOTOPE testing cohort). The BIOTOPE testing cohort has been described previously [10]. Although collected at a later date, the BIOTOPE training cohort was used for training purposes as it had a larger number of participants. All available data were used and learning curve analysis undertaken to determine if the model’s validation performance was plateauing. This study is reported as per the Transparent Reporting of a Multivariable Prediction Model for Individual Prognosis Or Diagnosis [2] AI statement [15] (TRIPOD AI Checklist in S1 Appendix).

### Data preprocessing

The variables included in this study are listed in the supplemental file (e2 Overview of BIOTOPE features in training model in S1 Appendix) and align with the Integrated Community Health Information System (iCHIS) in Malawi [16]. A critical challenge in prediction model development is ensuring that tools are implementable in the settings for which they are designed. Although our model includes a larger set of variables than traditional risk scores, these have been intentionally aligned with the data collected by the iCHIS being implemented by the Malawi Ministry of Health. This alignment ensures that the model can be deployed within existing community health workflows without adding extra data collection burden. Moreover, by drawing on iCHIS data—including household and community-level indicators in addition to clinical features—the model has the potential to leverage a richer feature set, allowing improved weighting of predictors and potentially more accurate risk stratification. Importantly, integration within iCHIS also facilitates sustainability: the model can be updated and retrained as new data accumulate, maintaining its relevance across changing epidemiological and health system contexts. For this study, we used a prespecified composite primary outcome defined as hospitalisation (admission to inpatient ward) and/or death within seven days of initial assessment. Due to only one death in this cohort, this model is best used to assess hospitalisation risk. Due to the nature of the outcome, it was not possible to blind assessors, but assessors were separate to those treating the children. Participant guardians were contacted by research assistants by phone for follow-up to collect outcome data. If more than 50% of a patient’s data or a feature’s data were missing, they were excluded from subsequent analysis. After removal of these patients/features, missing values were imputed using a *k*-nearest neighbour approach (Gower’s distance as the distance metric; *k* = 5) using the VIM R package (version 6.2.2) [17]. All categorical features were recoded as dummy variables using a dummy encoding using the fastDummies R package (version 1.7.3) [18].

### Model selection

We considered multiple hyperparameterisations across four off-shelf classifier families including: a support vector machine family (C×γ×kernel; C,γ ∈ {0.1,1,10}, kernel ∈ {radial basis function}), a naive Bayes family (α ∈ {0.1,1,10}), a logistic-activation deep-learning multilayer feed-forward neural network (multilayer perceptron) family (one-layer (${\text{layer}}_{\text{1}}$), two-layer (${layer}_{\text{1}}\times {layer}_{\text{2}}$) and three-layer (${layer}_{\text{1}}\times {layer}_{\text{2}}\times {layer}_{\text{3}}$) models were considered individually; all models were trained for a maximum of 10,000 iterations) (${layer}_{n}\;\in \;\{\text{1}\text{0},\text{5}\text{0},\text{1}\text{0}\text{0}\},\;n\;\in \;\{\text{1},\text{2},\text{3}\}$; where ${layer}_{n}=y$ denotes that there are y neurons in the nth layer), a random forest family ($n_{estimators}\times criterion\times n_{maximum\;features}$; ${\text{n}}_{estimators}\in \;\{\text{1}\text{0},\text{1}\text{0}\text{0},\text{1}\text{0}\text{0}\text{0}\}$, criterion ∈ {gini impurity, entropy}, $n_{\text{maximum features}}\;\in \;\{\sqrt{n_{\text{features}}},\;{\text{log}}_{\text{2}}(n_{\text{features}})\}$) where each tree was grown until maximal purity was achieved (each individual tree was trained on a randomly sampled-with-replacement sample of observations of size $n_{observations}$ ($n_{estimators},\;n_{features}$ and $n_{observations}$ correspond to the total number of trees, features and observations, respectively, in the supplied dataset while $n_{maximum\;features}$ corresponds to the maximum number of features used to build an individual tree)). XGBoost models (base learner×learning rate; base learner ∈ {tree, linear model}, learning rate ∈ {0.1, 0.3, 0.5}) were also explored using the xgboost Python package (version 2.1.4) [19,20]. All other models were trained using the scikit-learn Python package (version 1.6.0) [21]. Prior to classifier training, we performed feature selection by supplying each classifier with: (1) the complete set of selected features in the BIOTOPE training dataset, (2) the set of features remaining after application of near-zero variance feature selection [22], (3) the set of features remaining after application of Boruta feature selection [23] and (4) the set of features remaining after application of cluster-based filtering (i.e., selecting only one feature from each cluster, using the additive inverse of the Spearman correlation as a distance metric and Ward D2 as a clustering strategy; the silhouette method was used to determine the optimal number of clusters). We did not use class weights, undersampling/oversampling, cost-sensitive learning or any other approach to account for class imbalance.

All methods were configured to produce output probabilities for each patient observation. In order to choose the optimal model, we used a stratified *k*-fold cross-validation approach with *k* = 4. Specifically, we selected the combination of feature selection method, classifier family and corresponding hyperparameterisation that maximised the mean area under the precision-recall curve (PRAUC) (averaged across all *k* test folds) on the BIOTOPE training dataset from the space of all considered combinations. We also recorded the mean area under the receiver operating characteristic curve (AUC) [24]. In the case of model selection, probability calibration was performed after initial model training by fitting a logistic regression model to the relationship between predicted probability and observed response on previously unused data from the BIOTOPE training dataset; probability calibration was evaluated through both expected calibration error and through the parameter estimates derived from fitting a logistic regression model using the severity status as the response variable and the log odds of the calibrated probability as the predictor variable (under this approach, a perfectly calibrated predictive model should produce an intercept value of 0 and a slope value of 1). Nested cross-validation (with *j* = 4 outer folds) was also performed to evaluate the model selection procedure.

The selected model was externally validated on the BIOTOPE testing dataset. The selected model underwent retraining and recalibration on the BIOTOPE training dataset before application to the BIOTOPE testing dataset.

### Learning curve analysis

To estimate classifier performance as a function of the number of observations considered for analysis, we iteratively trained and tested the selected model on subsets of increasing size of the BIOTOPE training data. For each subset, we used stratified subsets of size 25% (514), 50% (1,027), 75% (1,540) and 100% (2053) of the entire BIOTOPE training dataset.

### Comparison to WHO severity criteria, RISC, RISC-Malawi, PERCH and PREPARE scores

In order to assess the performance of WHO severity criteria, RISC, RISC-Malawi, PERCH and PREPARE approaches on the BIOTOPE training and testing data, AUC and PRAUC performances on the BIOTOPE testing cohort were recorded for each approach. Where applicable, folds were identical to those used for assessing the performance of the approach selected from the model selection approach described above (Tables 1 and 2).

**Table 1:** Baseline characteristics.

Feature	Value	Unit	Training cohort	Testing cohort	*p*-value
**Demographic**					
Age		median (IQR)	15 (8–27)	17 (9–29)	
Age	2−12 months	*n*/total (%)	859/2053 (41.8%)	161/456 (35.3%)	0.03
Age	12−36 months	*n*/total (%)	917/2053 (44.7%)	223/456 (48.9%)	0.03
Age	36–60 months	*n*/total (%)	277/2053 (13.5%)	72/456 (15.8%)	0.03
Male		*n*/total (%)	1070/2053 (52.1%)	248/456 (54.4%)	0.41
**Assessment findings**					
Weight for age	<−3 SD	*n*/total (%)	69/2053 (3.4%)	6/447 (1.3%)	0.004
Weight for age	−3 to −2 SD	*n*/total (%)	148/2053 (7.2%)	32/447 (7.2%)	0.004
Weight for age	−2 to −1 SD	*n*/total (%)	430/2053 (20.9%)	66/447 (14.8%)	0.004
Weight for age	>−1 SD	*n*/total (%)	1406/2053 (68.5%)	343/447 (76.7%)	0.004
WHO danger sign present		*n*/total (%)	360/2050 (17.6%)	70/439 (15.9%)	0.44
History of fever		*n*/total (%)	1694/2053 (82.5%)	434/456 (95.2%)	*P* < 0.001
Cough		*n*/total (%)	1920/2053 (93.5%)	455/455 (100%)	*P* < 0.001
Wheeze		*n*/total (%)	377/2053 (18.4%)	30/447 (6.7%)	*P* < 0.001
Vomiting		*n*/total (%)	247/2053 (12%)	115/456 (25.2%)	*P* < 0.001
Diarrhoea		*n*/total (%)	157/2053 (7.6%)	22/456 (4.8%)	0.03
Sleepy		*n*/total (%)	16/2053 (0.8%)	7/439 (1.6%)	0.16
Runny nose		*n*/total (%)	377/2053 (18.4%)	238/456 (52.2%)	*P* < 0.001
Sneezing		*n*/total (%)	343/2053 (16.7%)	145/456 (31.8%)	*P* < 0.001
Grunting		*n*/total (%)	143/2053 (7%)	26/456 (5.7%)	0.35
Nasal flaring		*n*/total (%)	358/2053 (17.4%)	197/456 (43.2%)	*P* < 0.001
Chest indrawing		*n*/total (%)	1010/2053 (49.2%)	157/456 (34.4%)	*P* < 0.001
Head nodding		*n*/total (%)	60/2053 (2.9%)	16/456 (3.5%)	0.54
SpO2 < 90%		*n*/total (%)	80/2050 (3.9%)	20/445 (4.5%)	*P* > 0.99
Conscious level	Alert	*n*/total (%)	2050/2053 (99.9%)	453/453 (100%)	*P* > 0.99
	Responds to pain	*n*/total (%)	1/2053 (0%)	0/453 (0%)	*P* > 0.99
	Responds to Voice	*n*/total (%)	2/2053 (0.1%)	0/453 (0%)	*P* > 0.99
Unconscious		*n*/total (%)	2/2053 (0.1%)	0/456 (0%)	*P* > 0.99
Malaria RDT positive		*n*/total (%)	160/2053 (7.8%)	66/456 (14.5%)	*P* < 0.001
HIV–positive		*n*/total (%)	10/2053 (0.5%)	11/456 (2.4%)	*p* < 0.001
**Outcomes**					
Admitted to hospital		*n*/total (%)	294/2053 (14.3%)	55/456 (12.1%)	0.23
Death		*n*/total (%)	1/1872 (0.1%)	0/212 (0%)	*P* > 0.99

RDT, rapid diagnostic test, SpO2, peripheral oxygen saturation, WHO, World Health Organisation.

The WHO severity criteria, RISC, RISC-Malawi, PERCH and PREPARE approaches were also used as feature selectors. For each approach the raw (i.e., without the associated scoring criteria used by these approaches) set of predictor features was used to train the approach selected from the model selection approach described above.

### Feature importance analysis

We assessed feature importance using two distinct approaches: (1) a feature impurity-based approach, whereby features are progressively graded in importance as a result of how “pure” tree branches become (i.e., the heterogeneity of a branch with respect to the class outcome) as a result of splitting the data on that feature across all trees considered by the random forest [21] and (2) a permutation-based approach which computes importance as a function of the change in classification performance of the model resulting from using a permuted feature (i.e., randomly reassigning a feature’s values to patients such that any signal contained within the feature with respect to class outcome is perturbed) relative to the original unpermuted feature. We performed permutation-based feature importance after cluster-based feature selection as per the description above. Feature importance analysis was only performed on the BIOTOPE training cohort data; furthermore, derived importances were not used in any downstream analyses.

## Results

**Fig 1:**
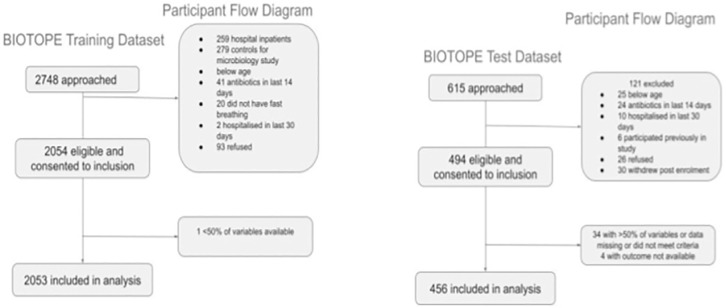
Participant flow diagram.

The BIOTOPE training cohort data was collected between December 2022 and April 2023 yielding 2053 children with data valid for analysis. The testing cohort data collected in 2,016 in primary care in Malawi [10] yielded 456 children with data valid for analysis (Fig 1).

### Prediction of severity of childhood pneumonia using machine learning in BIOTOPE training data

**Fig 2:**
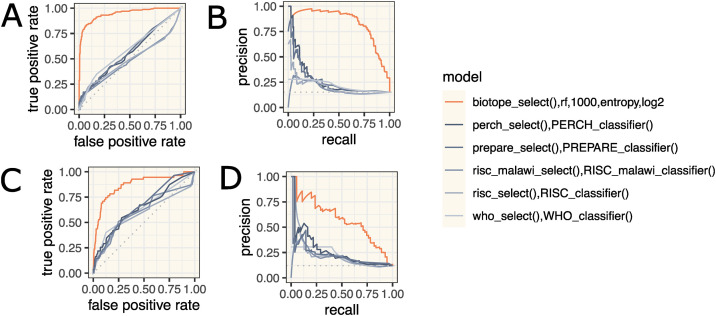
Performance of selected BIOTOPE-trained random forest classifier compared with WHO, RISC-Malawi, RISC, PERCH and PREPARE methods on BIOTOPE training data (A,B) and on BIOTOPE testing data (C,D).

We trained several machine learning classifiers on the BIOTOPE training data using stratified *k*-fold cross-validation and collected information on several performance metrics (Methods). Across all considered classifiers, a random forest classifier trained on all BIOTOPE features in the training dataset exhibited the best performance for both AUC and PRAUC, achieving a mean AUC of 0.95 (Standard Deviation (SD): 0.03) (e3 BIOTOPE model selection in S1 Appendix) and a mean PRAUC of 0.84 (SD: 0.06) (Fig 2 and Table 2); furthermore, this classifier yielded an expected calibration error of 0.08 (SD: 0.05) (a logistic regression model using severity status as the response variable and the log odds of the machine learning model’s calibrated probabilities produced an intercept estimate of 0.35 and a slope estimate of 1.11) (Fig C in S1 Appendix). With a view to quantifying the generalisation error of the cross-validated model selection approach, we employed a nested cross-validation procedure; this procedure yielded a mean AUC of 0.94 (SD: 0.03), a mean PRAUC of 0.84 (SD: 0.04) and an expected calibration error of 0.09 (SD: 0.06).

**Table 2:** Performance of WHO severity criteria, RISC-Malawi, RISC, PERCH and PREPARE methods as well as BIOTOPE random forest approach tested on BIOTOPE training and testing data.

Method	Training logistic regression parameters (intercept, slope)	Training PRAUC mean (sd)	Training AUC mean (sd)	Testing logistic regression parameters (intercept, slope)	Testing PRAUC	Testing AUC
BIOTOPE random forest	(0.35,1.11)	0.84 (0.06)	0.95 (0.03)	(−0.32,1.13)	0.57	0.87
PERCH		0.27 (0.08)	0.56 (0.09)		0.27	0.65
PREPARE		0.26 (0.07)	0.57 (0.07)		0.23	0.66
RISC		0.21 (0.06)	0.49 (0.11)		0.23	0.58
RISC-Malawi		0.18 (0.07)	0.48 (0.09)		0.20	0.61
WHO		0.22 (0.03)	0.59 (0.04)		0.22	0.64

AUC, area under curve, PRAUC, precision-recall area under curve.

In order to determine if children hospitalised without a current criterion for admission based on WHO danger signs could also be identified by the BIOTOPE algorithm, we evaluated the performance of the model in those without WHO danger signs (Figs G and H in S1 Appendix). In this case, the BIOTOPE random forest model (excluding children with a WHO danger sign) had an AUC of 0.95 (SD: 0.02) and a PRAUC of 0.80 (SD: 0.07) (probability calibration logistic regression intercept and slope (0.26,1.07) respectively). This is a clinically important subgroup, as these are the children most at risk of being missed by current guidelines. The model’s ability to identify at-risk children in this group provides evidence of the added value of this model.

### Learning curves for AUC and PRAUC in BIOTOPE training data

To estimate the relationship between classification performance and sample size, we trained and subsequently tested the selected random forest model on random subsets of the BIOTOPE training data using a stratified cumulative approach to preserve the class imbalance within each severity group (Methods). As expected, the model performed less well on the smallest subset ((mean AUC: 0.89 (SD: 0.07), mean PRAUC: 0.79 (SD: 0.12), probability calibration logistic regression intercept and slope (−0.21,0.95)) and performed best on the entire dataset (AUC: 0.95 (SD: 0.02), PRAUC: 0.85 (SD: 0.02), probability calibration logistic regression intercept and slope (0.15,1.15)) (S1 Appendix, Fig B and Table B).

### Comparison with other methods to predict severe pneumonia in BIOTOPE training data

We assessed the performance of several existing clinical prediction rules (WHO severity criteria, RISC, RISC-Malawi, PERCH and PREPARE) on the BIOTOPE training dataset using the same folds used for the model selection approach (Methods). Of the methods assessed, WHO severity criteria achieved the best performance on the BIOTOPE training dataset with respect to mean AUC (0.59 (SD: 0.04)) and the PERCH method achieved the best mean PRAUC (0.27 (SD: 0.08)). The performance of all methods fell below that of the selected random forest classifier trained on all features in the BIOTOPE training dataset (mean AUC: 0.95 (SD: 0.03), mean PRAUC: 0.84 (SD: 0.06), probability calibration logistic regression intercept and slope (0.35,1.11)) (Fig 2 and Table 2).

We also used each of the competing methods (WHO severity criteria, RISC, RISC-Malawi, PERCH and PREPARE) as feature selectors (Methods). For each selection, we trained a random forest classifier on the BIOTOPE training data. With the exception of WHO severity criteria, applying a random forest to the features comprising each feature set resulted in substantial improvements in performance compared to the original methods; the PREPARE method feature selection achieved the best performance of all competing feature selectors (AUC: 0.73 (SD: 0.05), PRAUC: 0.40 (SD: 0.08), probability calibration logistic regression intercept and slope (−0.21,0.77)) (S1 Appendix, Table C and Fig D). However, the performance achieved by training the random forest classifier on all features in the BIOTOPE training dataset remained better than the performance achieved by the random forest classifiers trained on the competing methods’ features (AUC: 0.95(SD: 0.03), PRAUC: 0.84 (SD: 0.06), probability calibration logistic regression intercept and slope (0.35,1.11)).

### Feature importance across BIOTOPE training data

**Fig 3:**
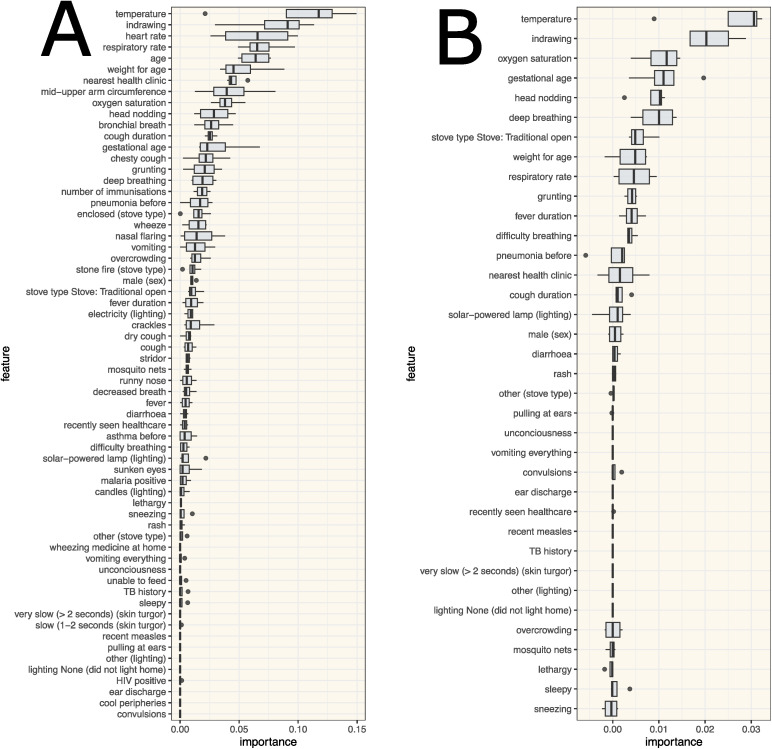
Feature importance results on BIOTOPE training data: (A) impurity-based importance (B) permutation-based importance.

Using an impurity-based approach to feature importance (Methods), axillary temperature was deemed considerably more important than any other feature (Fig 3). Following this, weight-for-age, indrawing, heart rate and respiratory rate were identified as the next most important features. Using a permutation-based approach to inferring feature importance after cluster-based feature selection (Methods), we once again identified temperature and indrawing among the most important features. Oxygen saturation and gestational age were among the additional important features identified using the permutation-based approach as well as the non-clinical feature of stove type used at home. We observed strong correlation among the median ranks of features using both approaches (correlation: 0.77; *p*-value <0.001) (Fig F in S1 Appendix).

### Dataset shift across BIOTOPE datasets

To externally test the derived random forest classifier trained on the BIOTOPE training dataset and assess dataset shift, we tested its performance on the BIOTOPE testing dataset (Methods). The trained classifier achieved a mean AUC of 0.87 and a mean PRAUC of 0.57 on the BIOTOPE testing dataset; this performance was superior to any of the compared methods (Fig 2 and Table 2). In addition, the model yielded an expected calibration error of 0.16 (a logistic regression model using severity status as the response variable and the log odds of the machine learning model’s calibrated probabilities produced an intercept estimate of −0.32 and a slope estimate of 1.13) (Fig C in S1 Appendix).

### Sensitivity of model performance to data imputation procedure

Data imputation facilitates the use of a larger amount of patient data; however, with a view to assessing the effect of the data imputation procedure on model performance, we applied the selected model to datasets filtered to retain only complete observations. When trained and tested on the filtered BIOTOPE training dataset (1,756 observations) (Methods), the model achieved an AUC of 0.95 (SD: 0.02) and a PRAUC of 0.84 (SD: 0.08) (probability calibration logistic regression intercept and slope (0.42,1.31)); when applied to the imputed BIOTOPE training dataset (2,053 observations), the model achieved an AUC of 0.95 (SD: 0.03) and a PRAUC of 0.84 (SD: 0.06) (probability calibration logistic regression intercept and slope (0.35,1.11)). In comparison, when trained on the filtered BIOTOPE training dataset and tested on the filtered BIOTOPE testing dataset (104 observations), the model achieved an AUC of 0.86 and a PRAUC of 0.71 (probability calibration logistic regression intercept and slope (0.75,1.22)); when trained on the imputed BIOTOPE training dataset and tested on the imputed BIOTOPE testing dataset (460 observations), the model achieved an AUC of 0.87 and a PRAUC of 0.57 (probability calibration logistic regression intercept and slope (−0.32,1.13)).

#### Patient and public involvement

In developing the study involvement from public and carers of children with pneumonia was undertaken and a sculpture created by local artists showing the expectations from them in terms of outcomes if successful (e10 Public Involvement in S1 Appendix). Additional information regarding the ethical, cultural, and scientific considerations specific to inclusivity in global research is included in the Supporting information (S1 Inclusivity in Global Research Checklist)

## Discussion

This study demonstrates that a machine learning algorithm based on a random forest classifier, trained and tested in two separate cohorts, has good predictive ability in identifying those at risk of hospitalisation in children presenting with WHO-defined pneumonia in primary care in Malawi. We compared the performance of the calibrated predictive model trained on the BIOTOPE training data with respect to the observed BIOTOPE testing data. The trained model produced an AUC of 0.87 and a PRAUC of 0.57. In terms of evaluation of predicted probability calibration, the trained model achieved an expected calibration error of 0.16; in addition, we fitted a logistic regression model to the observed severity status using the log odds of the calibrated predicted probability as the sole explanatory variable; given that the true log odds of a case of severe pneumonia should be equal to the log odds predicted by a perfectly calibrated model and that, under the logistic regression framework, the estimated log odds are equal to the sum of a constant intercept term and the product of the calibrated log odds with a slope term, an ideal logistic regression model should estimate a zero-valued intercept term and an estimated slope of unity for a perfectly calibrated model; for the logistic regression model, we observed estimated intercept and slope terms of −0.32 and 1.13, respectively; these estimates can be used to compute estimated probabilities and characterise the variation between the calibrated predictive model and the logistic regression model; in particular, for example, according to the logistic regression model estimates, when the predicted model estimates a 50% probability of severe pneumonia, the logistic regression estimates a 42% probability of severe pneumonia (calibrated model estimate: 10% -> logistic regression estimate: 6%, 25% -> 17%, 50% -> 42%, 75% -> 72%, 90% -> 90%). The performance of this classifier was superior to a number of existing risk prediction models in predicting this outcome (WHO [4,11], RISC [12], RISC-Malawi [14], PERCH [13] and PREPARE [14] across BIOTOPE datasets. It is important to note that these risk models involved hospitalised patients and used mortality alone as an outcome measure, implying a sicker cohort than BIOTOPE, which involved primary care patients only. Notwithstanding this, the superior performance achieved by the random forest could result from the additional features available to the classifier, from a more optimal use of the same features or from a combination of both. To distinguish between these possibilities, we trained the random forest classifier using only the variables found in the existing risk scores without their derived weighting as per these scores. With the exception of WHO severity criteria, applying a random forest to the features comprising each feature set resulted in substantial improvements in performance compared to the original methods, suggesting that the original splitting points, feature weightings and/or feature interactions used by these methods are not optimal for the BIOTOPE datasets. However the random forest classifier trained on all BIOTOPE features still outperformed all other methods. The superior performance of the random forest classifier on the full set of features relative to all other approaches suggests that the additional features collected in the BIOTOPE study may provide additional predictive power. These non-traditional features include markers of socio-economic deprivation [25] and household environment [26] that have been shown to be predictive of pneumonia, and are based on features routinely collected by community health workers in Malawi.

There have been concerns regarding the ability of risk scores to predict poor outcomes in child pneumonia [5,10,27] An external validation study noted that although pneumonia risk scores have performed well among the cohorts in which they were derived, their performance diminished when externally applied [28]. A study from Malawi showed only 37.2% of children hospitalised with pneumonia had a WHO danger sign [27]. Although concerns may arise that this is due to admission of low risk children, a further study showed 39% of fatal cases of pneumonia in children in hospital in Kenya were defined as having non-severe pneumonia [5]. We used approaches to identify variables which are likely to be important in the random forest model. The use of these approaches to infer explainability is important given concerns regarding model bias and dataset shift [29]. We observed strong correlation between impurity-based and permutation-based approaches for evaluating variable importance. The identified variables, such as chest indrawing, heart rate, temperature and markers of nutrition, have been associated with poorer outcomes in other studies. This provides reassurance that the model is clinically sensible.

A recent WHO stakeholder consultative meeting [30] emphasised the need to evaluate the use of technologies to improve pneumonia diagnosis and treatment; furthermore, the meeting also highlighted that current risk assessment tools are poorly defined and/or not validated. Prediction of risk is challenging in clinical practice. Over the course of time, shifts in population demographics, disease prevalence, clinical practices, and the healthcare system can occur. Such changes may render predictions based on static data obsolete and unreliable, a phenomenon commonly referred to as dataset shift [29]. This poses a major challenge when employing risk scores and prediction models in practice. This can be seen with the varying model prediction metrics seen in this study and, indeed, between the training and testing cohorts in this study also. Current approaches to risk prediction are often static approaches where a model is derived from one cohort and then validated in another cohort before deployment. In many cases, continuous monitoring and evaluation of the model’s discrimination and calibration is not possible, and changes in model performance can be hard to detect in a timely manner. A possible solution would involve the development of models capable of (1) continuous updates in real-time as new information becomes available and (2) integrating this information into routine healthcare data collection to enable continuous monitoring and evaluation. This dynamic approach aims to provide accurate risk predictions that adapt swiftly to evolving data, thereby minimising reliance on outdated models. It also allows for adaptation to new pathogens or changing clinical practice. This approach not only reduces the dependency on outdated models but also streamlines the process of cohort data collection by avoiding the creation and utilisation of multiple models, ultimately saving time and effort. Compared to existing risk scores, we have implemented an approach that can be continuously retrained and redeployed using machine learning. This does not involve continuous monitoring of individual patients. Instead, it refers to using routinely collected healthcare data, as it becomes available, to track model performance and retrain the algorithm when necessary. In this way, the model can adapt to changes such as seasonality or regional variation without requiring a complete offline redevelopment process. In practice, the algorithm can monitor its own accuracy and, if performance deteriorates, either update itself to restore accuracy or flag the need for review by an external panel. A robust AI governance framework is essential to ensure that updates are not triggered by trivial fluctuations, while substantial shifts (e.g., emergence of new pathogens) are carefully investigated and understood. This approach facilitates the integration of models into routine healthcare data streams, supporting their ongoing clinical relevance and reliability.

We intend to evaluate the integration of this tool as an eHealth tool for use in Malawi by community health workers, allowing for continuous retraining of the score as well as for other public health approaches such as proactive machine learning [31]. When the model is used to guide hospitalisation decisions, there is a risk that, during retraining, it may begin to learn institutional admission practices rather than true clinical severity. Moreover, by influencing patient management decisions, the model may inadvertently alter the data on which it is subsequently trained, thereby degrading its own performance. This creates a dependency between the model’s prediction (severity, defined by hospitalisation and its recommended action (hospitalisation based on predicted severity).

To mitigate this circularity, future approaches could aim to decouple prediction from clinical action by redefining the target outcome as clinical deterioration rather than hospitalisation. Deterioration could be defined by objective indicators such as re-presentation to healthcare within a specified timeframe, prolonged hospital stays, or escalation to a higher level of care. Another potential safeguard would be to record clinicians’ intended management decisions before model input, enabling comparison between model predictions and independent clinical judgment, and allowing assessment of the model’s true added value. At scale, variation in climate, resources, and clinician training would also naturally introduce diversity in decision-making allowing monitoring of model performance and clinical outcomes, but continuous oversight by an AI governance committee would be essential to monitor these. Ultimately, clinical impact studies will be necessary to determine the model’s real-world effectiveness.

This study involves datasets derived from primary care where children with pneumonia are primarily seen but where fewer studies are conducted. It involves datasets which are separated in time and space to demonstrate the generalisability of this approach. However, there are a number of limitations in this study. We used a marker of severity defined by hospitalisation and/or death within seven days of assessment. Study participants may have been hospitalised for reasons other than severe pneumonia and hospitalisation is a subjective decision dependent on a number of factors relating to the health system, health worker and family. Death is a more objective representation, but is a rare outcome in primary care cohorts with only one death in our cohort. This is consistent with other studies showing low mortality in primary care [32–35]. However, in primary care, one of the main objectives is to appropriately identify those who require hospitalisation. As such, we consider that this is a useful initial outcome measure. Further studies validating this–and alternative–score(s) should be conducted. Previous studies have shown that of children who died in Malawi most caregivers brought their child to a healthcare setting, and many sought care multiple times from different healthcare providers, suggesting there are clinical issues in identifying children with severe pneumonia [36]. The prediction tools used for comparison (RISC, RISC-Malawi, PERCH and PREPARE) were originally developed and validated in hospital populations, rather than in children presenting in outpatient settings. In addition, these tools were designed to predict mortality, whereas the BIOTOPE dataset contained too few deaths to reliably distinguish between children who died and those who survived. Nevertheless, comparison with these established tools is still important, as they represent the current benchmarks in paediatric pneumonia risk stratification. Evaluating their performance in a primary care population provides valuable insight into their generalizability and highlights the potential added value of the BIOTOPE tool in broader clinical contexts. Furthermore, the difference in outcome is clinically relevant: in primary care, the central question for health workers is not only whether a child will survive, but whether they are at risk of deterioration and therefore require referral for higher-level care with treatment failure being a concern [37]. While mortality is a more objective endpoint, it is rare in outpatient cohorts such as BIOTOPE and is influenced by multiple health system factors beyond the initial clinical presentation. By contrast, hospitalisation is a pragmatic and actionable outcome that reflects real-world decision-making in primary care. Nevertheless, we recognise that both the outcome definition and the predictor features may affect model performance. The approach to missing or incomplete data in the model requires further investigation in terms of impact on prediction ability and methods to mitigate this. The imputation method described in the methods section can be used, but further research into its efficacy is required. This should be considered when comparing across models and requires further validation and clinical impact studies.

Finally, as alluded to in the above, WHO definition of pneumonia based on clinical signs (fast breathing and/or chest indrawing) is sensitive but not specific and so children may have had other conditions in addition to, or, instead of, pneumonia [1]. Despite this shortcoming, this definition represents the current standard used in primary healthcare to identify and treat children. Although the model contains more variables than traditionally used in a risk score, it does align with the variables collected in the iCHIS system as developed by the Malawi Ministry of Health. As such, the model can be deployed in a real-world setting without extra data collection burden on workers and cut offs for referral developed to enable clear guidance on need for hospital referral or close monitoring. Work is currently underway to integrate this score directly into the iCHIS system.

The BIOTOPE machine learning algorithm provided a substantial improvement over existing methods to assess risk of hospitalisation in childhood pneumonia in our cohorts; in particular, the selected model achieved strong results with respect to AUC, PRAUC and probability calibration. However, further validation in larger and more diverse datasets is required with a focus on outcomes such as mortality and deterioration. If proven to be useful, integrating machine learning scores into routine clinical assessment using existing clinical management tools may allow ongoing monitoring and adaptation of methods to assess severity. Such monitoring not only facilitates optimal performance, but also allows for rapid updating of scores, particularly if new markers of severity are identified. Clinical impact studies will be important to ensure that these innovations result in improved clinical outcomes for patients and such studies should be undertaken in a way that takes account of broader considerations for the appropriate implementation and governance of AI strategies in healthcare. Embedding the tool within iCHIS will not only enhance its immediate usability in Malawi but also provide a framework for scalable adaptation to other geographic settings. In doing so, this approach moves beyond demonstrating predictive performance of machine learning tools to creating a tool that is both clinically relevant and sustainable in real-world practice. Further work is ongoing to implement this.

## Supporting information

S1 Appendix Supplemental File. e1: BIOTOPE study sites. e2: Overview of BIOTOPE features in training model. e3: BIOTOPE model selection. e4: BIOTOPE model performance. e5: Feature importance in BIOTOPE data. e6: Effects of varying WHO danger sign representation in BIOTOPE data. e7: Machine learning glossary. e8: Immunisation status of those included in BIOTOPE. e9: BIOTOPE study training schedule. e10: Public Involvement. e11: Link to Github repository. e12: Tripod AI checklist. e13 Discrimination of existing childhood pneumonia risk scores vs. BIOTOPE. e14 Overview of the Integrated Community Health Information System (iCHIS) in Malawi.
(DOCX)

S1 Inclusivity in Global Health Checklist (PDF)

## Abbreviations:

AUC: Area Under the Curve
iCCM: integrated Community Case Management
iCHIS: Integrated community health information system
IMCI: Integrated Management of Childhood Illness
ML: machine learning
PERCH: Pneumonia Etiology Research for Child Health
PRAUC: precision recall area under curve
PREPARE: Pneumonia Research Partnership to Assess WHO Recommendations
RISC: Respiratory Index of Severity in Children
SD: Standard Deviation
WHO: World Health Organisation
