# Supplementary material for: Prediction of hospitalisation in young children with pneumonia in Malawi: A machine learning-based approach
Source: PLoS Med. 2026 Jun 9;23(6):e1005122. doi: 10.1371/journal.pmed.1005122 (PMC13271506; doi:10.1371/journal.pmed.1005122)
Supplement: S1 Appendix — e1: BIOTOPE study sites. e2: Overview of BIOTOPE features in training model. e3: BIOTOPE model selection. e4: BIOTOPE model performance. e5: Feature importance in BIOTOPE data. e6: Effects of varying WHO danger sign representation in BIOTOPE data. e7: Machine learning glossary. e8: Immunisation status of those included in BIOTOPE. e9: BIOTOPE study training schedule. e10: Public Involvement. e11: Link to Github repository. e12: Tripod AI checklist. e13 Discrimination of existing childhood pneumonia risk scores vs. BIOTOPE. e14 Overview of the Integrated Community Health Information System (iCHIS) in Malawi. (DOCX) [file pmed.1005122.s001.docx]

**Online Only Supplemental File:**

**S1 A Machine Learning Algorithm to Predict Hospitalisation in Childhood Pneumonia**

**Contents:**

[**e1: BIOTOPE study sites** 1](#_Toc230953814)

[**e2: Overview of BIOTOPE features in training model** 2](#_Toc230953815)

[**e3: BIOTOPE model selection** 10](#_Toc230953816)

[**e4: BIOTOPE model performance** 22](#_Toc230953817)

[**e5: Feature importance in BIOTOPE data** 26](#_Toc230953818)

[**e6: Effects of varying WHO danger sign representation in BIOTOPE data** 27](#_Toc230953819)

[**e7: Machine learning glossary** 28](#_Toc230953820)

[**e8: Immunisation status of those included in BIOTOPE** 32](#_Toc230953821)

[**e9: BIOTOPE study training schedule** 32](#_Toc230953822)

[**e10: Public Involvement** 33](#_Toc230953823)

[**e11: Link to Github repository** 35](#_Toc230953824)

[**e12: Tripod AI checklist** 36](#_Toc230953825)

[**e13 Discrimination of existing childhood pneumonia risk scores vs. BIOTOPE** 37](#_Toc230953826)

[**e14 Overview of the Integrated Community Health Information System (iCHIS) in Malawi** 39](#_Toc230953827)

# **e1: BIOTOPE study sites**

| **Site Name** | **Type** | **Status** |
| --- | --- | --- |
| Bwengu | Health Centre | Training |
| Bolero | Health Centre | Training |
| Jenda | Health Centre | Training |
| Mapale | Health Centre | Training (2022) Test (2016) |
| Mpamba Road | Health Centre | Training |
| Chinteche | Health Centre | Training |
| Rumphi | District Hospital | Training |
| Mzimba South | District Hospital | Training |
| Nkhata Bay | District Hospital | Training |
| Mzuzu Central Hospital OPD | Central Hospital | Test |

**Table A: Study sites for BIOTOPE project**

# **e2: Overview of BIOTOPE features in training model**

| **BIOTOPE** | **Feature type** | **RISC** | **PERCH** | **PREPARE** |
| --- | --- | --- | --- | --- |
| **Age** | Demographic |  | Yes | Yes |
| **Sex** | Demographic |  | Yes | Yes |
| **Weight for age** | Demographic | Yes |  | Yes |
| **Gestational age** | Demographic |  |  |  |
| **Vomiting everything** | Symptoms |  |  |  |
| **Convulsions** | Symptoms |  |  | Yes |
| **Lethargy** | Symptoms |  |  |  |
| **Unconsciousness** | Symptoms |  | Yes | Yes |
| **Unable to feed** | Symptoms | Refusing feeds |  |  |
| **Stridor** | Symptoms |  |  |  |
| **Recent attendance at healthcare provider** | Demographic |  |  |  |
| **Previous admission for pneumonia** | Demographic |  |  |  |
| **Diagnosis of asthma** | Demographic |  |  |  |
| **Use of wheezing medicine at home** | Demographic |  |  |  |
| **HIV positive** | Demographic | Yes |  |  |
| **Personal history of TB** | Demographic |  |  |  |
| **Malaria RDT positive** | Demographic |  |  |  |
| **Fever reported by caregiver** | Symptoms |  |  |  |
| **Cough** | Symptoms |  |  |  |
| **Difficulty Breathing** | Symptoms |  |  |  |
| **Wheeze** | Symptoms | Yes |  |  |
| **Any vomiting** | Symptoms |  |  |  |
| **Diarrhoea** | Symptoms |  |  |  |
| **Sleepy** | Symptoms |  |  |  |
| **Runny nose** | Symptoms |  |  |  |
| **Sneezing** | Symptoms |  |  |  |
| **Pulling at ears** | Symptoms |  |  |  |
| **Ear discharge** | Symptoms |  |  |  |
| **Sunken eyes** | Symptoms |  |  |  |
| **Cool peripheries** | Symptoms |  |  |  |
| **Grunting** | Symptoms |  | Yes |  |
| **Nasal flaring** | Symptoms |  |  |  |
| **Deep breathing** | Symptoms |  | Yes |  |
| **Chest indrawing** | Symptoms | Yes |  |  |
| **Head nodding** | Symptoms |  |  |  |
| **Rash** | Symptoms |  |  |  |
| **Decreased breath sounds** | Symptoms |  |  |  |
| **Bronchial breath sounds** | Symptoms |  |  |  |
| **Crackles in chest** | Symptoms |  |  |  |
| **Dry cough** | Symptoms |  | Cough |  |
| **Chesty cough** | Symptoms |  |  |  |
| **Fever duration** | Symptoms |  | Duration of illness |  |
| **Cough duration** | Symptoms |  |  |  |
| **Recent measles** | Demographic |  |  |  |
| **Temperature (axillary)** | Symptoms |  |  | Yes |
| **Heart rate** | Symptoms |  |  |  |
| **Respiratory rate** | Symptoms |  |  | Yes |
| **Oxygen saturation** | Symptoms | Hypoxaemia | Hypoxaemia | Yes |
| **Mid upper arm circumference** | Demographic |  |  |  |
| **Overcrowding** | Demographic |  |  |  |
| **Sleeps under mosquito net** | Demographic |  |  |  |
| **Time to travel to nearest health clinic** | Demographic |  |  |  |
| **Number of immunisations** | Demographic |  |  |  |
| **Skin turgor** | Symptoms |  |  |  |
| **Cooking type in house** | Demographic |  |  |  |
| **Lighting type in house** | Demographic |  |  |  |
| **Presence of WHO danger sign** | Symptoms |  |  |  |
|  |  |  | Weight for height | Cyanosis |

**Table B: Overview of BIOTOPE features and their appearance in comparative methods**

**
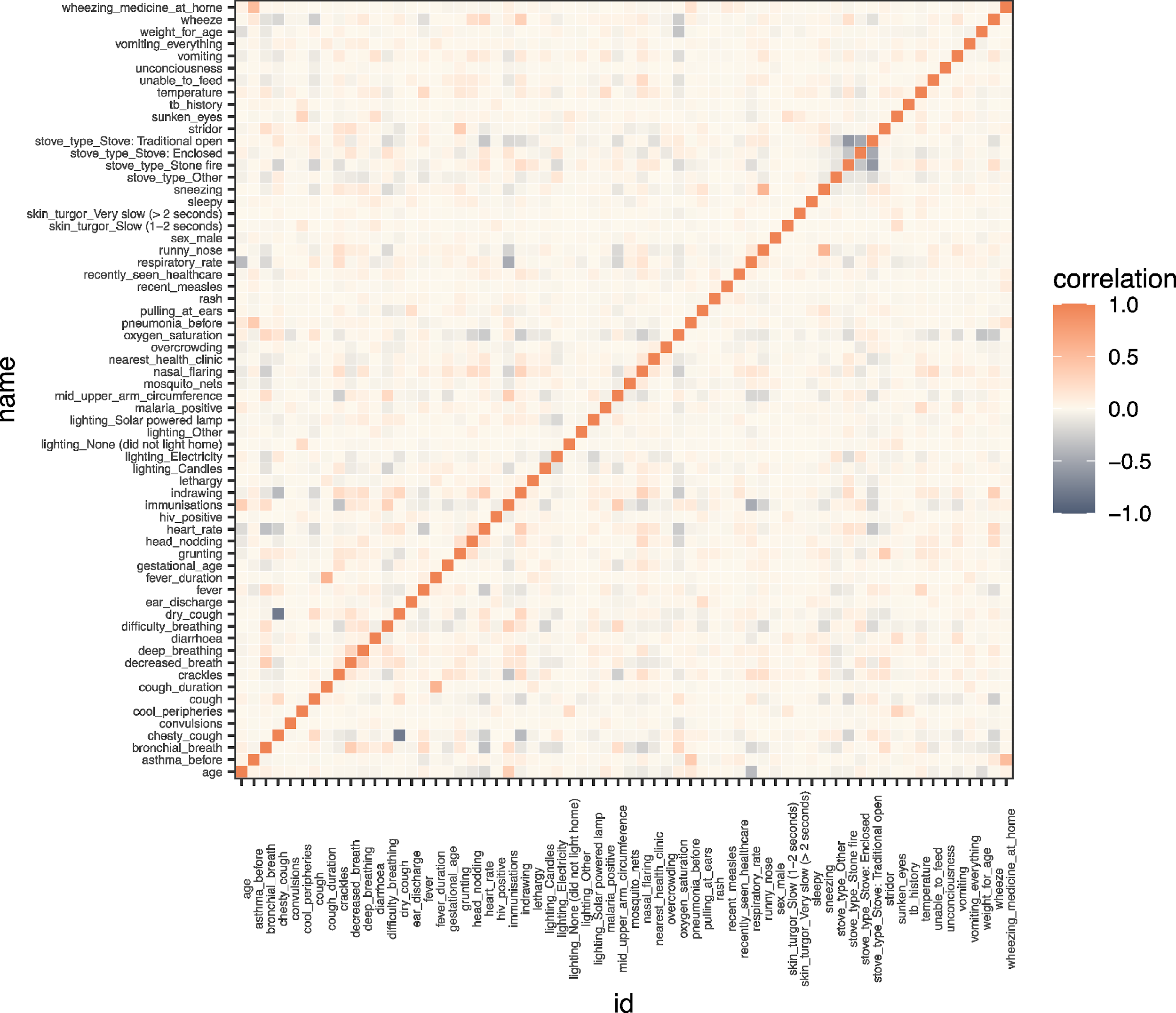
**

**Figure A: Correlation matrix of BIOTOPE features**

| name | # missing |
| --- | --- |
| age | 0 |
| sex_male | 0 |
| vomiting_everything | 0 |
| convulsions | 0 |
| lethargy | 0 |
| unconsciousness | 0 |
| unable_to_feed | 0 |
| stridor | 0 |
| malaria_positive | 0 |
| fever | 0 |
| difficulty_breathing | 0 |
| vomiting | 0 |
| diarrhoea | 0 |
| runny_nose | 0 |
| sneezing | 0 |
| pulling_at_ears | 0 |
| grunting | 0 |
| nasal_flaring | 0 |
| deep_breathing | 0 |
| indrawing | 0 |
| head_nodding | 0 |
| dry_cough | 0 |
| chesty_cough | 0 |
| overcrowding | 0 |
| id | 0 |
| cohort | 0 |
| pneumonia_before | 1 |
| cough | 1 |
| ear_discharge | 1 |
| lighting_Candles | 1 |
| lighting_Electricity | 1 |
| lighting_None (did not light home) | 1 |
| lighting_Other | 1 |
| lighting_Solar powered lamp | 1 |
| skin_turgor_Slow (1-2 seconds) | 2 |
| skin_turgor_Very slow (> 2 seconds) | 2 |
| asthma_before | 3 |
| wheezing_medicine_at_home | 3 |
| cool_peripheries | 3 |
| stove_type_Other | 3 |
| stove_type_Stone fire | 3 |
| stove_type_Stove: Enclosed | 3 |
| stove_type_Stove: Traditional open | 3 |
| sunken_eyes | 4 |
| recent_measles | 4 |
| target | 4 |
| admitted_hospital | 4 |
| mosquito_nets | 5 |
| recently_seen_healthcare | 6 |
| heart_rate | 6 |
| nearest_health_clinic | 6 |
| respiratory_rate | 7 |
| gestational_age | 8 |
| weight_for_age | 9 |
| wheeze | 10 |
| decreased_breath | 10 |
| bronchial_breath | 10 |
| crackles | 11 |
| rash | 14 |
| oxygen_saturation | 14 |
| sleepy | 17 |
| tb_history | 20 |
| temperature | 20 |
| danger_sign | 20 |
| mid_upper_arm_circumference | 26 |
| hiv_positive | 59 |
| immunisations | 87 |
| fever_duration | 102 |
| cough_duration | 109 |
| child_still_alive | 429 |

**Table C: Missing data counts for all BIOTOPE features**

| WHO danger sign/severity | non-severe | severe |
| --- | --- | --- |
| absent | 1,833 | 226 |
| present | 309 | 121 |

**Table D: 2x2 table of the relationship between WHO danger sign and severity**

# **e3: BIOTOPE model selection**

| model | mean AUC (sd) | mean PRAUC (sd) |
| --- | --- | --- |
| biotope_select(),rf,1000,entropy,log2 | 0.95 (0.03) | 0.84 (0.06) |
| biotope_select(),rf,1000,entropy,sqrt | 0.95 (0.03) | 0.84 (0.05) |
| biotope_select(),rf,1000,gini,sqrt | 0.94 (0.03) | 0.83 (0.06) |
| biotope_select(),rf,1000,gini,log2 | 0.94 (0.03) | 0.83 (0.06) |
| biotope_select(),rf,100,gini,sqrt | 0.94 (0.03) | 0.83 (0.05) |
| biotope_select(),rf,100,entropy,log2 | 0.94 (0.03) | 0.83 (0.04) |
| biotope_select(),rf,100,entropy,sqrt | 0.94 (0.02) | 0.83 (0.06) |
| biotope_select(),rf,100,gini,log2 | 0.95 (0.02) | 0.82 (0.05) |
| biotope_select(),xgb,gbtree,0.5 | 0.93 (0.02) | 0.81 (0.05) |
| biotope_select(),rf,100,entropy,None | 0.93 (0.02) | 0.8 (0.05) |
| biotope_select(),xgb,gbtree,0.3 | 0.93 (0.02) | 0.8 (0.05) |
| biotope_select(),rf,1000,entropy,None | 0.93 (0.02) | 0.8 (0.04) |
| biotope_corr_select(),rf,100,gini,log2 | 0.93 (0.02) | 0.8 (0.05) |
| biotope_select(),xgb,gbtree,0.1 | 0.93 (0.02) | 0.8 (0.05) |
| biotope_corr_select(),rf,100,gini,sqrt | 0.93 (0.02) | 0.79 (0.06) |
| biotope_corr_select(),rf,1000,entropy,log2 | 0.93 (0.02) | 0.79 (0.06) |
| biotope_corr_select(),rf,1000,entropy,sqrt | 0.93 (0.02) | 0.79 (0.06) |
| biotope_corr_select(),rf,1000,gini,sqrt | 0.93 (0.02) | 0.79 (0.07) |
| biotope_corr_select(),rf,1000,gini,log2 | 0.94 (0.02) | 0.79 (0.07) |
| biotope_corr_select(),rf,100,entropy,sqrt | 0.93 (0.02) | 0.78 (0.06) |
| biotope_corr_select(),rf,100,entropy,log2 | 0.93 (0.03) | 0.78 (0.06) |
| biotope_select(),svm,1.0,0.1,rbf | 0.93 (0.03) | 0.77 (0.05) |
| biotope_select(),rf,1000,gini,None | 0.93 (0.03) | 0.77 (0.05) |
| biotope_select(),svm,10.0,0.1,rbf | 0.92 (0.02) | 0.77 (0.02) |
| biotope_select(),rf,10,gini,sqrt | 0.92 (0.03) | 0.77 (0.07) |
| biotope_select(),rf,100,gini,None | 0.92 (0.03) | 0.76 (0.06) |
| biotope_corr_select(),xgb,gbtree,0.1 | 0.92 (0.03) | 0.75 (0.07) |
| biotope_corr_select(),rf,1000,entropy,None | 0.91 (0.01) | 0.75 (0.05) |
| biotope_select(),rf,10,entropy,log2 | 0.89 (0.04) | 0.75 (0.05) |
| biotope_corr_select(),xgb,gbtree,0.3 | 0.91 (0.02) | 0.75 (0.05) |
| biotope_corr_select(),xgb,gbtree,0.5 | 0.92 (0.02) | 0.74 (0.07) |
| biotope_select(),rf,10,entropy,sqrt | 0.9 (0.02) | 0.74 (0.06) |
| biotope_corr_select(),rf,100,entropy,None | 0.91 (0.01) | 0.74 (0.04) |
| biotope_select(),rf,10,gini,log2 | 0.91 (0.03) | 0.74 (0.04) |
| biotope_corr_select(),svm,1.0,0.1,rbf | 0.92 (0.03) | 0.73 (0.05) |
| biotope_select(),rf,10,entropy,None | 0.89 (0.02) | 0.73 (0.04) |
| biotope_corr_select(),rf,10,gini,log2 | 0.89 (0.03) | 0.73 (0.06) |
| biotope_corr_select(),rf,1000,gini,None | 0.92 (0.02) | 0.73 (0.05) |
| biotope_select(),mlp,(100, 50, 10) | 0.9 (0.02) | 0.72 (0.03) |
| biotope_select(),svm,0.1,0.1,rbf | 0.92 (0.03) | 0.71 (0.07) |
| biotope_select(),mlp,(100, 100) | 0.88 (0.03) | 0.71 (0.05) |
| biotope_corr_select(),rf,10,gini,sqrt | 0.89 (0.04) | 0.71 (0.07) |
| biotope_select(),mlp,100 | 0.89 (0.03) | 0.71 (0.01) |
| biotope_select(),mlp,(100, 50) | 0.89 (0.03) | 0.71 (0.04) |
| biotope_select(),mlp,(100, 10, 10) | 0.9 (0.02) | 0.71 (0.03) |
| biotope_corr_select(),svm,10.0,0.1,rbf | 0.9 (0.02) | 0.71 (0.04) |
| biotope_corr_select(),rf,100,gini,None | 0.91 (0.02) | 0.7 (0.06) |
| biotope_select(),mlp,50 | 0.9 (0.02) | 0.7 (0.01) |
| biotope_corr_select(),rf,10,entropy,None | 0.88 (0.03) | 0.7 (0.06) |
| biotope_corr_select(),rf,10,entropy,log2 | 0.9 (0.02) | 0.69 (0.03) |
| biotope_select(),mlp,(100, 50, 50) | 0.87 (0.03) | 0.69 (0.04) |
| biotope_select(),mlp,(100, 100, 100) | 0.87 (0.04) | 0.69 (0.05) |
| biotope_corr_select(),rf,10,entropy,sqrt | 0.89 (0.04) | 0.69 (0.04) |
| biotope_corr_select(),mlp,(100, 100, 10) | 0.86 (0.03) | 0.69 (0.02) |
| biotope_corr_select(),mlp,(50, 50, 10) | 0.87 (0.03) | 0.68 (0.02) |
| biotope_corr_select(),mlp,(50, 100, 10) | 0.86 (0.04) | 0.68 (0.05) |
| biotope_select(),mlp,(50, 10) | 0.87 (0.03) | 0.68 (0.03) |
| biotope_select(),mlp,(100, 50, 100) | 0.87 (0.01) | 0.68 (0.05) |
| biotope_select(),rf,10,gini,None | 0.89 (0.04) | 0.68 (0.09) |
| biotope_corr_select(),mlp,(100, 10, 100) | 0.88 (0.03) | 0.68 (0.03) |
| biotope_select(),mlp,(50, 50) | 0.88 (0.03) | 0.68 (0.04) |
| biotope_corr_select(),mlp,(100, 100, 100) | 0.87 (0.03) | 0.68 (0.01) |
| biotope_select(),mlp,(100, 100, 50) | 0.86 (0.02) | 0.68 (0.02) |
| biotope_select(),mlp,(100, 10) | 0.88 (0.01) | 0.68 (0.04) |
| biotope_select(),mlp,(50, 50, 100) | 0.87 (0.02) | 0.68 (0.03) |
| biotope_corr_select(),mlp,(100, 100) | 0.87 (0.03) | 0.67 (0.02) |
| biotope_corr_select(),svm,0.1,0.1,rbf | 0.91 (0.03) | 0.67 (0.07) |
| biotope_corr_select(),mlp,(100, 10, 10) | 0.87 (0.04) | 0.67 (0.05) |
| biotope_select(),mlp,(50, 50, 50) | 0.87 (0.02) | 0.67 (0.04) |
| biotope_corr_select(),mlp,(100, 50, 10) | 0.86 (0.03) | 0.67 (0.01) |
| biotope_corr_select(),mlp,(100, 50) | 0.87 (0.04) | 0.67 (0.04) |
| biotope_select(),mlp,(10, 50, 10) | 0.88 (0.02) | 0.67 (0.07) |
| biotope_select(),mlp,(10, 100, 10) | 0.88 (0.02) | 0.67 (0.06) |
| biotope_select(),mlp,(100, 10, 100) | 0.88 (0.03) | 0.67 (0.05) |
| biotope_select(),mlp,(100, 10, 50) | 0.86 (0.01) | 0.67 (0.04) |
| biotope_select(),mlp,(50, 100) | 0.88 (0.04) | 0.67 (0.04) |
| biotope_corr_select(),mlp,(100, 100, 50) | 0.86 (0.04) | 0.67 (0.02) |
| biotope_corr_select(),mlp,100 | 0.89 (0.02) | 0.67 (0.03) |
| biotope_corr_select(),mlp,(100, 50, 100) | 0.86 (0.03) | 0.67 (0.05) |
| biotope_select(),mlp,(50, 50, 10) | 0.88 (0.02) | 0.67 (0.04) |
| biotope_select(),mlp,(50, 10, 50) | 0.86 (0.01) | 0.67 (0.03) |
| biotope_corr_select(),mlp,(50, 10, 10) | 0.87 (0.04) | 0.67 (0.04) |
| biotope_corr_select(),mlp,(10, 10) | 0.87 (0.02) | 0.66 (0.05) |
| biotope_select(),mlp,(50, 100, 10) | 0.87 (0.03) | 0.66 (0.05) |
| biotope_corr_select(),mlp,(50, 100) | 0.87 (0.03) | 0.66 (0.01) |
| biotope_corr_select(),mlp,50 | 0.89 (0.02) | 0.66 (0.03) |
| biotope_corr_select(),mlp,(50, 100, 100) | 0.87 (0.03) | 0.66 (0.03) |
| biotope_select(),mlp,10 | 0.88 (0.02) | 0.66 (0.04) |
| biotope_select(),mlp,(100, 100, 10) | 0.88 (0.02) | 0.66 (0.06) |
| biotope_corr_select(),mlp,(50, 10) | 0.87 (0.03) | 0.66 (0.05) |
| biotope_corr_select(),mlp,(100, 50, 50) | 0.86 (0.03) | 0.66 (0.04) |
| biotope_corr_select(),mlp,(50, 100, 50) | 0.86 (0.03) | 0.66 (0.04) |
| biotope_corr_select(),mlp,(100, 10) | 0.86 (0.04) | 0.65 (0.05) |
| biotope_corr_select(),mlp,(50, 50, 50) | 0.86 (0.04) | 0.65 (0.05) |
| biotope_select(),xgb,gblinear,0.5 | 0.89 (0.02) | 0.65 (0.02) |
| biotope_corr_select(),mlp,(100, 10, 50) | 0.87 (0.03) | 0.65 (0.04) |
| biotope_select(),mlp,(50, 100, 100) | 0.86 (0.02) | 0.65 (0.03) |
| biotope_select(),mlp,(10, 50, 100) | 0.86 (0.04) | 0.65 (0.02) |
| biotope_select(),mlp,(50, 10, 100) | 0.86 (0.01) | 0.65 (0.02) |
| biotope_select(),mlp,(10, 10, 50) | 0.87 (0.01) | 0.65 (0.02) |
| biotope_select(),xgb,gblinear,0.3 | 0.89 (0.03) | 0.65 (0.01) |
| biotope_select(),mlp,(50, 10, 10) | 0.87 (0.02) | 0.65 (0.04) |
| biotope_corr_select(),mlp,(10, 50, 50) | 0.85 (0.03) | 0.64 (0.05) |
| biotope_select(),mlp,(10, 50) | 0.86 (0.03) | 0.64 (0.03) |
| biotope_select(),mlp,(50, 100, 50) | 0.84 (0.03) | 0.64 (0.04) |
| biotope_select(),mlp,(10, 100, 50) | 0.86 (0.02) | 0.64 (0.03) |
| biotope_corr_select(),mlp,(50, 50) | 0.87 (0.04) | 0.64 (0.02) |
| biotope_corr_select(),mlp,10 | 0.89 (0.01) | 0.64 (0.07) |
| biotope_corr_select(),xgb,gblinear,0.5 | 0.88 (0.02) | 0.64 (0.03) |
| biotope_corr_select(),mlp,(10, 50, 10) | 0.85 (0.02) | 0.63 (0.03) |
| biotope_corr_select(),mlp,(50, 10, 50) | 0.86 (0.02) | 0.63 (0.05) |
| biotope_corr_select(),mlp,(10, 100, 50) | 0.84 (0.04) | 0.63 (0.06) |
| biotope_corr_select(),mlp,(10, 50, 100) | 0.87 (0) | 0.63 (0.04) |
| biotope_corr_select(),mlp,(10, 10, 10) | 0.87 (0.02) | 0.63 (0.06) |
| biotope_corr_select(),xgb,gblinear,0.3 | 0.88 (0.02) | 0.63 (0.03) |
| biotope_select(),xgb,gblinear,0.1 | 0.88 (0.03) | 0.63 (0.01) |
| biotope_corr_select(),mlp,(50, 50, 100) | 0.86 (0.04) | 0.63 (0.04) |
| biotope_select(),mlp,(10, 10, 10) | 0.87 (0.02) | 0.63 (0.02) |
| biotope_select(),mlp,(10, 10) | 0.87 (0.03) | 0.63 (0.08) |
| biotope_corr_select(),mlp,(10, 50) | 0.86 (0.03) | 0.62 (0.08) |
| biotope_corr_select(),mlp,(50, 10, 100) | 0.85 (0.03) | 0.62 (0.02) |
| biotope_corr_select(),mlp,(10, 100, 10) | 0.85 (0.02) | 0.62 (0.05) |
| biotope_select(),mlp,(10, 50, 50) | 0.86 (0.02) | 0.62 (0.03) |
| biotope_corr_select(),rf,10,gini,None | 0.87 (0.01) | 0.62 (0.05) |
| biotope_corr_select(),mlp,(10, 10, 100) | 0.84 (0.02) | 0.61 (0.04) |
| biotope_corr_select(),xgb,gblinear,0.1 | 0.87 (0.02) | 0.6 (0.02) |
| biotope_corr_select(),mlp,(10, 100) | 0.84 (0.01) | 0.6 (0.04) |
| biotope_corr_select(),mlp,(10, 100, 100) | 0.82 (0.05) | 0.6 (0.06) |
| biotope_select(),nb,0.1 | 0.87 (0.01) | 0.59 (0.04) |
| biotope_select(),mlp,(10, 100) | 0.86 (0.01) | 0.59 (0.02) |
| biotope_select(),nb,1.0 | 0.87 (0.01) | 0.59 (0.04) |
| biotope_select(),mlp,(10, 100, 100) | 0.85 (0.04) | 0.58 (0.09) |
| biotope_select(),mlp,(10, 10, 100) | 0.85 (0.03) | 0.58 (0.05) |
| biotope_corr_select(),mlp,(10, 10, 50) | 0.83 (0.03) | 0.58 (0.1) |
| biotope_corr_select(),svm,0.1,1.0,rbf | 0.87 (0.04) | 0.57 (0.06) |
| biotope_corr_select(),svm,1.0,1.0,rbf | 0.87 (0.04) | 0.57 (0.06) |
| biotope_select(),nb,10.0 | 0.85 (0.01) | 0.54 (0.04) |
| biotope_corr_select(),nb,0.1 | 0.84 (0.01) | 0.52 (0.03) |
| biotope_corr_select(),nb,1.0 | 0.84 (0.01) | 0.52 (0.03) |
| biotope_corr_select(),svm,10.0,1.0,rbf | 0.86 (0.04) | 0.52 (0.04) |
| biotope_corr_select(),nb,10.0 | 0.83 (0.01) | 0.49 (0.03) |
| biotope_select(),svm,0.1,1.0,rbf | 0.85 (0.04) | 0.49 (0.05) |
| biotope_select(),svm,1.0,1.0,rbf | 0.85 (0.04) | 0.49 (0.05) |
| biotope_select(),svm,10.0,1.0,rbf | 0.85 (0.04) | 0.48 (0.05) |
| biotope_boruta_select(),rf,1000,entropy,log2 | 0.77 (0.04) | 0.48 (0.07) |
| biotope_boruta_select(),rf,1000,entropy,sqrt | 0.77 (0.04) | 0.48 (0.07) |
| biotope_boruta_select(),xgb,gbtree,0.1 | 0.75 (0.05) | 0.48 (0.06) |
| biotope_boruta_select(),rf,1000,gini,log2 | 0.78 (0.04) | 0.48 (0.08) |
| biotope_boruta_select(),rf,1000,gini,sqrt | 0.78 (0.04) | 0.48 (0.08) |
| biotope_boruta_select(),rf,100,gini,log2 | 0.78 (0.04) | 0.47 (0.07) |
| biotope_boruta_select(),rf,100,gini,sqrt | 0.78 (0.04) | 0.47 (0.07) |
| biotope_boruta_select(),rf,100,entropy,log2 | 0.76 (0.03) | 0.47 (0.07) |
| biotope_boruta_select(),rf,100,entropy,sqrt | 0.76 (0.03) | 0.47 (0.07) |
| biotope_boruta_select(),rf,1000,entropy,None | 0.76 (0.05) | 0.47 (0.07) |
| biotope_boruta_select(),rf,1000,gini,None | 0.77 (0.04) | 0.47 (0.07) |
| biotope_boruta_select(),rf,100,gini,None | 0.77 (0.05) | 0.46 (0.08) |
| biotope_boruta_select(),rf,100,entropy,None | 0.75 (0.05) | 0.46 (0.07) |
| biotope_boruta_select(),rf,10,gini,log2 | 0.74 (0.06) | 0.45 (0.07) |
| biotope_boruta_select(),rf,10,gini,sqrt | 0.74 (0.06) | 0.45 (0.07) |
| biotope_boruta_select(),xgb,gbtree,0.5 | 0.73 (0.04) | 0.44 (0.07) |
| biotope_boruta_select(),rf,10,entropy,log2 | 0.72 (0.02) | 0.44 (0.06) |
| biotope_boruta_select(),rf,10,entropy,sqrt | 0.72 (0.02) | 0.44 (0.06) |
| biotope_boruta_select(),svm,0.1,1.0,rbf | 0.72 (0.04) | 0.44 (0.04) |
| biotope_boruta_select(),xgb,gbtree,0.3 | 0.73 (0.05) | 0.43 (0.08) |
| biotope_boruta_select(),rf,10,gini,None | 0.73 (0.04) | 0.43 (0.08) |
| biotope_boruta_select(),svm,10.0,0.1,rbf | 0.71 (0.04) | 0.43 (0.08) |
| biotope_boruta_select(),mlp,(100, 100, 10) | 0.77 (0.03) | 0.43 (0.04) |
| biotope_boruta_select(),mlp,(100, 10, 100) | 0.76 (0.04) | 0.43 (0.04) |
| biotope_boruta_select(),mlp,(50, 10, 50) | 0.77 (0.04) | 0.43 (0.04) |
| biotope_boruta_select(),mlp,(100, 10, 50) | 0.76 (0.04) | 0.42 (0.04) |
| biotope_boruta_select(),mlp,(100, 10, 10) | 0.75 (0.05) | 0.42 (0.05) |
| biotope_boruta_select(),mlp,(100, 10) | 0.76 (0.05) | 0.42 (0.07) |
| biotope_boruta_select(),rf,10,entropy,None | 0.72 (0.04) | 0.42 (0.05) |
| biotope_boruta_select(),svm,1.0,1.0,rbf | 0.71 (0.05) | 0.42 (0.07) |
| biotope_boruta_select(),mlp,(100, 50) | 0.76 (0.05) | 0.42 (0.07) |
| biotope_boruta_select(),mlp,(50, 50) | 0.76 (0.05) | 0.41 (0.07) |
| biotope_boruta_select(),mlp,(100, 50, 10) | 0.77 (0.03) | 0.41 (0.04) |
| biotope_boruta_select(),mlp,(100, 100) | 0.76 (0.05) | 0.41 (0.07) |
| biotope_boruta_select(),mlp,(100, 50, 100) | 0.76 (0.05) | 0.41 (0.07) |
| biotope_boruta_select(),mlp,(50, 10, 100) | 0.76 (0.05) | 0.41 (0.07) |
| biotope_boruta_select(),mlp,(50, 10, 10) | 0.76 (0.05) | 0.41 (0.06) |
| biotope_boruta_select(),mlp,(100, 50, 50) | 0.76 (0.05) | 0.41 (0.07) |
| biotope_boruta_select(),svm,1.0,0.1,rbf | 0.73 (0.05) | 0.41 (0.06) |
| biotope_boruta_select(),svm,0.1,0.1,rbf | 0.73 (0.05) | 0.4 (0.05) |
| biotope_boruta_select(),mlp,(50, 100, 10) | 0.76 (0.03) | 0.4 (0.06) |
| biotope_boruta_select(),mlp,(10, 10, 50) | 0.76 (0.05) | 0.4 (0.06) |
| biotope_boruta_select(),mlp,(10, 100, 50) | 0.75 (0.05) | 0.4 (0.06) |
| biotope_boruta_select(),mlp,(10, 100, 100) | 0.75 (0.05) | 0.4 (0.06) |
| biotope_boruta_select(),mlp,(10, 10) | 0.76 (0.05) | 0.4 (0.07) |
| biotope_boruta_select(),mlp,(10, 100) | 0.75 (0.05) | 0.4 (0.06) |
| biotope_boruta_select(),mlp,(50, 50, 10) | 0.76 (0.04) | 0.39 (0.05) |
| biotope_boruta_select(),mlp,(50, 50, 100) | 0.75 (0.05) | 0.39 (0.07) |
| biotope_boruta_select(),mlp,(50, 100) | 0.75 (0.05) | 0.39 (0.07) |
| biotope_boruta_select(),mlp,(100, 100, 50) | 0.75 (0.05) | 0.39 (0.07) |
| biotope_boruta_select(),mlp,(100, 100, 100) | 0.75 (0.05) | 0.39 (0.07) |
| biotope_boruta_select(),mlp,(10, 50, 10) | 0.75 (0.04) | 0.39 (0.05) |
| biotope_boruta_select(),mlp,(10, 10, 10) | 0.75 (0.05) | 0.39 (0.06) |
| biotope_boruta_select(),mlp,50 | 0.75 (0.05) | 0.39 (0.07) |
| biotope_boruta_select(),mlp,(50, 100, 100) | 0.75 (0.05) | 0.39 (0.07) |
| biotope_boruta_select(),mlp,(10, 50) | 0.75 (0.05) | 0.39 (0.06) |
| biotope_boruta_select(),mlp,(10, 50, 50) | 0.75 (0.05) | 0.39 (0.06) |
| biotope_boruta_select(),mlp,(50, 10) | 0.75 (0.05) | 0.39 (0.06) |
| biotope_boruta_select(),mlp,(50, 100, 50) | 0.76 (0.05) | 0.39 (0.06) |
| biotope_boruta_select(),xgb,gblinear,0.5 | 0.76 (0.05) | 0.39 (0.07) |
| biotope_boruta_select(),mlp,(50, 50, 50) | 0.75 (0.05) | 0.39 (0.07) |
| biotope_boruta_select(),xgb,gblinear,0.3 | 0.76 (0.05) | 0.39 (0.07) |
| biotope_boruta_select(),mlp,100 | 0.75 (0.05) | 0.39 (0.07) |
| biotope_boruta_select(),mlp,(10, 10, 100) | 0.74 (0.05) | 0.39 (0.07) |
| biotope_boruta_select(),mlp,10 | 0.75 (0.05) | 0.39 (0.07) |
| biotope_boruta_select(),xgb,gblinear,0.1 | 0.75 (0.05) | 0.38 (0.07) |
| biotope_boruta_select(),mlp,(10, 100, 10) | 0.73 (0.06) | 0.37 (0.06) |
| biotope_boruta_select(),mlp,(10, 50, 100) | 0.75 (0.04) | 0.37 (0.05) |
| biotope_corr_select(),svm,10.0,10.0,rbf | 0.75 (0.06) | 0.34 (0.08) |
| biotope_corr_select(),svm,1.0,10.0,rbf | 0.75 (0.06) | 0.34 (0.08) |
| biotope_boruta_select(),svm,0.1,10.0,rbf | 0.72 (0.04) | 0.34 (0.07) |
| biotope_boruta_select(),svm,10.0,1.0,rbf | 0.68 (0.05) | 0.33 (0.04) |
| biotope_corr_select(),svm,0.1,10.0,rbf | 0.74 (0.06) | 0.33 (0.09) |
| biotope_boruta_select(),svm,1.0,10.0,rbf | 0.72 (0.04) | 0.33 (0.07) |
| biotope_boruta_select(),nb,10.0 | 0.7 (0.04) | 0.31 (0.04) |
| biotope_boruta_select(),svm,10.0,10.0,rbf | 0.7 (0.04) | 0.31 (0.07) |
| biotope_boruta_select(),nb,0.1 | 0.7 (0.04) | 0.3 (0.03) |
| biotope_boruta_select(),nb,1.0 | 0.7 (0.05) | 0.3 (0.03) |
| biotope_select(),svm,10.0,10.0,rbf | 0.62 (0.02) | 0.25 (0.04) |
| biotope_select(),svm,1.0,10.0,rbf | 0.61 (0.03) | 0.23 (0.06) |
| biotope_select(),svm,0.1,10.0,rbf | 0.59 (0.03) | 0.22 (0.05) |
| biotope_nzv_select(),svm,10.0,0.1,rbf | 0.56 (0.07) | 0.21 (0.05) |
| biotope_nzv_select(),svm,0.1,0.1,rbf | 0.56 (0.09) | 0.19 (0.04) |
| biotope_nzv_select(),svm,0.1,10.0,rbf | 0.55 (0.06) | 0.19 (0.03) |
| biotope_nzv_select(),svm,0.1,1.0,rbf | 0.54 (0.06) | 0.19 (0.05) |
| biotope_nzv_select(),mlp,(10, 10) | 0.56 (0.07) | 0.19 (0.03) |
| biotope_nzv_select(),mlp,(10, 10, 10) | 0.56 (0.07) | 0.19 (0.03) |
| biotope_nzv_select(),mlp,(10, 10, 100) | 0.56 (0.07) | 0.19 (0.03) |
| biotope_nzv_select(),mlp,(10, 10, 50) | 0.56 (0.07) | 0.19 (0.03) |
| biotope_nzv_select(),mlp,(10, 100) | 0.56 (0.07) | 0.19 (0.03) |
| biotope_nzv_select(),mlp,(10, 100, 10) | 0.56 (0.07) | 0.19 (0.03) |
| biotope_nzv_select(),mlp,(10, 100, 100) | 0.56 (0.07) | 0.19 (0.03) |
| biotope_nzv_select(),mlp,(10, 100, 50) | 0.56 (0.07) | 0.19 (0.03) |
| biotope_nzv_select(),mlp,(10, 50) | 0.56 (0.07) | 0.19 (0.03) |
| biotope_nzv_select(),mlp,(10, 50, 10) | 0.56 (0.07) | 0.19 (0.03) |
| biotope_nzv_select(),mlp,(10, 50, 100) | 0.56 (0.07) | 0.19 (0.03) |
| biotope_nzv_select(),mlp,(100, 10) | 0.56 (0.07) | 0.19 (0.03) |
| biotope_nzv_select(),mlp,(100, 10, 10) | 0.56 (0.07) | 0.19 (0.03) |
| biotope_nzv_select(),mlp,(100, 10, 100) | 0.56 (0.07) | 0.19 (0.03) |
| biotope_nzv_select(),mlp,(100, 10, 50) | 0.56 (0.07) | 0.19 (0.03) |
| biotope_nzv_select(),mlp,(100, 100) | 0.56 (0.07) | 0.19 (0.03) |
| biotope_nzv_select(),mlp,(100, 100, 10) | 0.56 (0.07) | 0.19 (0.03) |
| biotope_nzv_select(),mlp,(100, 100, 100) | 0.56 (0.07) | 0.19 (0.03) |
| biotope_nzv_select(),mlp,(100, 100, 50) | 0.56 (0.07) | 0.19 (0.03) |
| biotope_nzv_select(),mlp,(100, 50) | 0.56 (0.07) | 0.19 (0.03) |
| biotope_nzv_select(),mlp,(100, 50, 10) | 0.56 (0.07) | 0.19 (0.03) |
| biotope_nzv_select(),mlp,(100, 50, 100) | 0.56 (0.07) | 0.19 (0.03) |
| biotope_nzv_select(),mlp,(100, 50, 50) | 0.56 (0.07) | 0.19 (0.03) |
| biotope_nzv_select(),mlp,(50, 10) | 0.56 (0.07) | 0.19 (0.03) |
| biotope_nzv_select(),mlp,(50, 10, 10) | 0.56 (0.07) | 0.19 (0.03) |
| biotope_nzv_select(),mlp,(50, 10, 100) | 0.56 (0.07) | 0.19 (0.03) |
| biotope_nzv_select(),mlp,(50, 10, 50) | 0.56 (0.07) | 0.19 (0.03) |
| biotope_nzv_select(),mlp,(50, 100) | 0.56 (0.07) | 0.19 (0.03) |
| biotope_nzv_select(),mlp,(50, 100, 10) | 0.56 (0.07) | 0.19 (0.03) |
| biotope_nzv_select(),mlp,(50, 100, 100) | 0.56 (0.07) | 0.19 (0.03) |
| biotope_nzv_select(),mlp,(50, 100, 50) | 0.56 (0.07) | 0.19 (0.03) |
| biotope_nzv_select(),mlp,(50, 50) | 0.56 (0.07) | 0.19 (0.03) |
| biotope_nzv_select(),mlp,(50, 50, 10) | 0.56 (0.07) | 0.19 (0.03) |
| biotope_nzv_select(),mlp,(50, 50, 100) | 0.56 (0.07) | 0.19 (0.03) |
| biotope_nzv_select(),mlp,(50, 50, 50) | 0.56 (0.07) | 0.19 (0.03) |
| biotope_nzv_select(),mlp,10 | 0.56 (0.07) | 0.19 (0.03) |
| biotope_nzv_select(),mlp,100 | 0.56 (0.07) | 0.19 (0.03) |
| biotope_nzv_select(),mlp,50 | 0.56 (0.07) | 0.19 (0.03) |
| biotope_nzv_select(),xgb,gblinear,0.1 | 0.56 (0.07) | 0.19 (0.03) |
| biotope_nzv_select(),xgb,gblinear,0.3 | 0.56 (0.07) | 0.19 (0.03) |
| biotope_nzv_select(),xgb,gblinear,0.5 | 0.56 (0.07) | 0.19 (0.03) |
| biotope_nzv_select(),mlp,(10, 50, 50) | 0.55 (0.07) | 0.19 (0.03) |
| biotope_nzv_select(),svm,1.0,10.0,rbf | 0.52 (0.03) | 0.18 (0.05) |
| biotope_nzv_select(),svm,1.0,0.1,rbf | 0.55 (0.07) | 0.18 (0.03) |
| biotope_nzv_select(),svm,1.0,1.0,rbf | 0.55 (0.04) | 0.18 (0.01) |
| biotope_nzv_select(),nb,0.1 | 0.55 (0.04) | 0.17 (0.01) |
| biotope_nzv_select(),nb,1.0 | 0.55 (0.04) | 0.17 (0.01) |
| biotope_nzv_select(),nb,10.0 | 0.55 (0.04) | 0.17 (0.01) |
| biotope_nzv_select(),rf,1000,entropy,None | 0.47 (0.03) | 0.16 (0.02) |
| biotope_nzv_select(),rf,1000,entropy,log2 | 0.47 (0.03) | 0.16 (0.02) |
| biotope_nzv_select(),rf,1000,entropy,sqrt | 0.47 (0.03) | 0.16 (0.02) |
| biotope_nzv_select(),rf,1000,gini,None | 0.47 (0.03) | 0.16 (0.02) |
| biotope_nzv_select(),rf,1000,gini,log2 | 0.47 (0.03) | 0.16 (0.02) |
| biotope_nzv_select(),rf,1000,gini,sqrt | 0.47 (0.03) | 0.16 (0.02) |
| biotope_nzv_select(),svm,10.0,10.0,rbf | 0.54 (0.04) | 0.16 (0.01) |
| biotope_nzv_select(),rf,100,entropy,None | 0.47 (0.03) | 0.16 (0.02) |
| biotope_nzv_select(),rf,100,entropy,log2 | 0.47 (0.03) | 0.16 (0.02) |
| biotope_nzv_select(),rf,100,entropy,sqrt | 0.47 (0.03) | 0.16 (0.02) |
| biotope_nzv_select(),rf,100,gini,None | 0.47 (0.03) | 0.16 (0.02) |
| biotope_nzv_select(),rf,100,gini,log2 | 0.47 (0.03) | 0.16 (0.02) |
| biotope_nzv_select(),rf,100,gini,sqrt | 0.47 (0.03) | 0.16 (0.02) |
| biotope_nzv_select(),rf,10,entropy,None | 0.49 (0.04) | 0.16 (0.02) |
| biotope_nzv_select(),rf,10,entropy,log2 | 0.49 (0.04) | 0.16 (0.02) |
| biotope_nzv_select(),rf,10,entropy,sqrt | 0.49 (0.04) | 0.16 (0.02) |
| biotope_nzv_select(),rf,10,gini,None | 0.49 (0.04) | 0.16 (0.02) |
| biotope_nzv_select(),rf,10,gini,log2 | 0.49 (0.04) | 0.16 (0.02) |
| biotope_nzv_select(),rf,10,gini,sqrt | 0.49 (0.04) | 0.16 (0.02) |
| biotope_nzv_select(),svm,10.0,1.0,rbf | 0.48 (0.08) | 0.16 (0.02) |
| biotope_nzv_select(),xgb,gbtree,0.5 | 0.49 (0.04) | 0.16 (0.02) |
| biotope_nzv_select(),xgb,gbtree,0.3 | 0.48 (0.04) | 0.15 (0.03) |
| biotope_nzv_select(),xgb,gbtree,0.1 | 0.48 (0.04) | 0.15 (0.03) |

**Table E: Performance of model selection candidates on BIOTOPE training/evaluation data**

# **e4: BIOTOPE model performance**


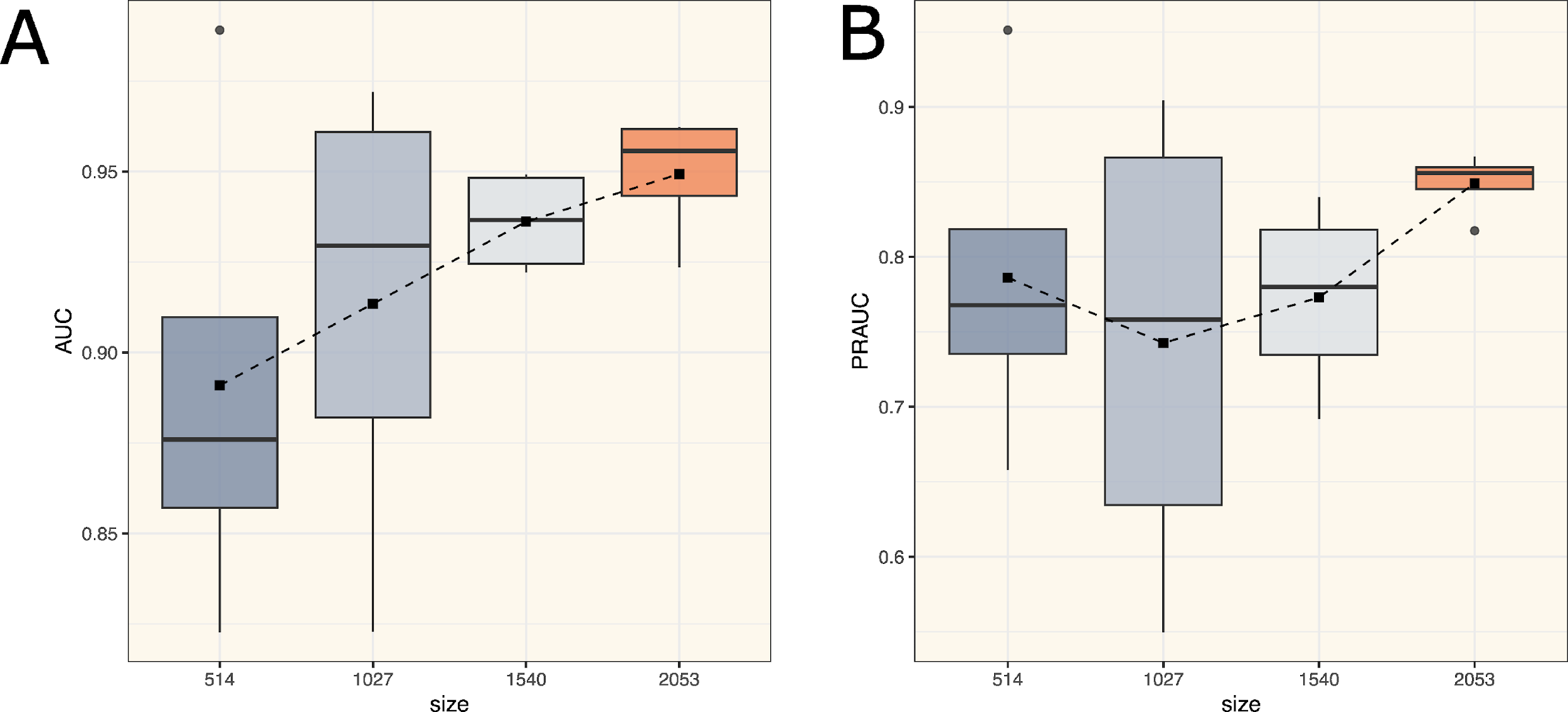


**Figure B: Performance statistics on BIOTOPE training data as a function of dataset size**

**
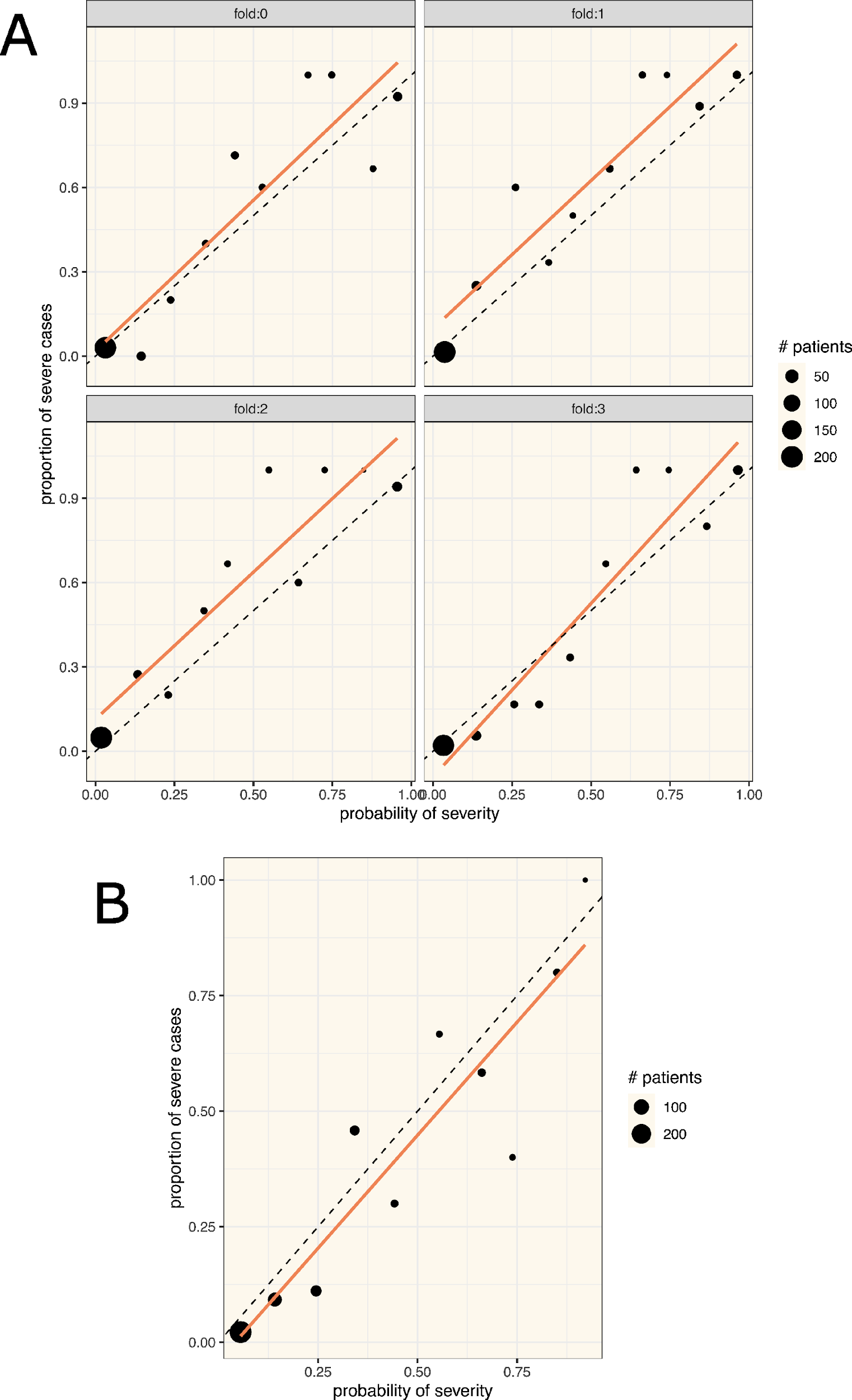
**

**Figure C:**  **Probability calibration results for**   **(A) nested calibration on BIOTOPE training dataset and**   **(B) selected BIOTOPE model on BIOTOPE testing dataset; both calibrated probabilities (x-axis) and severity status (y-axis) are sorted into bins of size 0.1 from 0 to 1 based on the calibrated probabilities; the black broken line represents a line of perfect fit and the orange solid line represents the fitted line between proportion of severe cases and probability of severity**

| subset size | logistic regression parameters (intercept, slope) | mean PRAUC (SD) | mean AUC (SD) |
| --- | --- | --- | --- |
| 514 ( 25%) | (-0.21,0.95) | 0.79 ( 0.12) | 0.89 ( 0.07) |
| 1027 ( 50%) | (-0.13,1.18) | 0.74 ( 0.17) | 0.91 ( 0.07) |
| 1540 ( 75%) | (0.58,1.30) | 0.77 ( 0.07) | 0.94 ( 0.01) |
| 2053 ( 100%) | (0.15,1.15) | 0.85 ( 0.02) | 0.95 ( 0.02) |
|  |  |  |  |

**Table F: Performance of the selected BIOTOPE model on a cumulatively increasing range of subsets of the BIOTOPE training dataset (using stratified subsets) (logistic regression parameters correspond to the logistic regression model fitted using the severity status as the response variable and log odds of the calibrated probability as the predictor variable)**

| method | training logistic regression parameters (intercept, slope) | mean training PRAUC (sd) | mean training AUC (sd) | testing logistic regression parameters (intercept, slope) | testing PRAUC | testing AUC |
| --- | --- | --- | --- | --- | --- | --- |
| BIOTOPE features | (0.35, 1.11) | 0.84 (0.06) | 0.95 (0.03) | (-0.32,1.13) | 0.57 | 0.87 |
| PERCH features | (-0.01, 0.96) | 0.36 (0.03) | 0.72 (0.05) | (1.29,1.87) | 0.40 | 0.74 |
| PREPARE features | (-0.21, 0.77) | 0.40 (0.08) | 0.73 (0.05) | (-0.94,0.81) | 0.30 | 0.70 |
| RISC features | (0.03,0.95) | 0.22 (0.03) | 0.6 (0.03) | (-0.67,0.77) | 0.14 | 0.56 |
| RISC-Malawi features | (0.49,1.20) | 0.18 (0.07) | 0.48 (0.09) | (-0.06,1.19) | 0.23 | 0.67 |
| WHO features | (-0.37,0.73) | 0.22 (0.03) | 0.59 (0.04) | (5.78,4.71) | 0.17 | 0.61 |

**Table G: Performance of selected random forest classifier in combination with WHO, RISC-Malawi, RISC, PERCH and PREPARE feature selection methods as well as approach trained using all BIOTOPE features (logistic regression parameters correspond to the logistic regression model fitted using the severity status as the response variable and log odds of the calibrated probability as the predictor variable)**

**
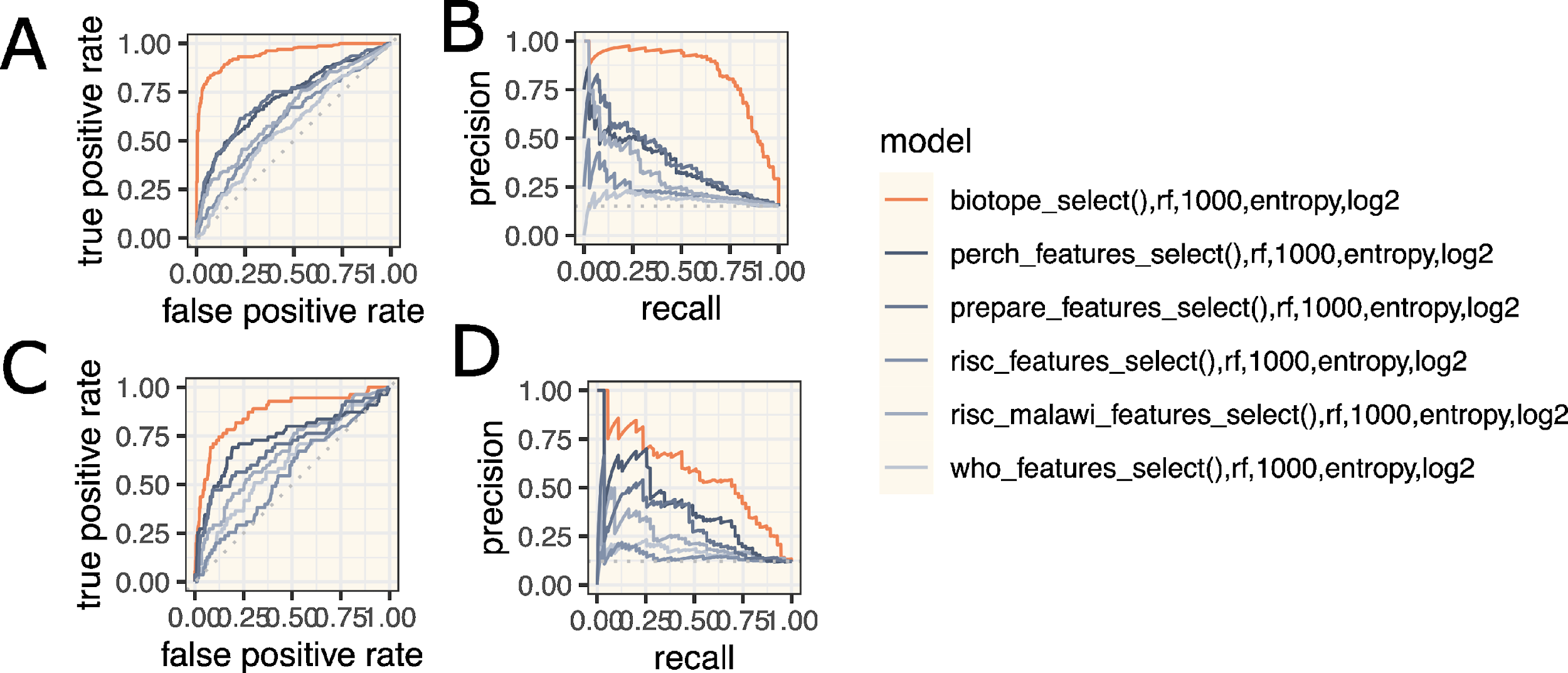
**

**Figure D: Performance of selected random forest classifier in combination with WHO, RISC-Malawi, RISC, PERCH and PREPARE feature selection methods as well as approach trained using all BIOTOPE features on BIOTOPE training data (A,B) and BIOTOPE testing data (C,D)**

# **e5: Feature importance in BIOTOPE data**


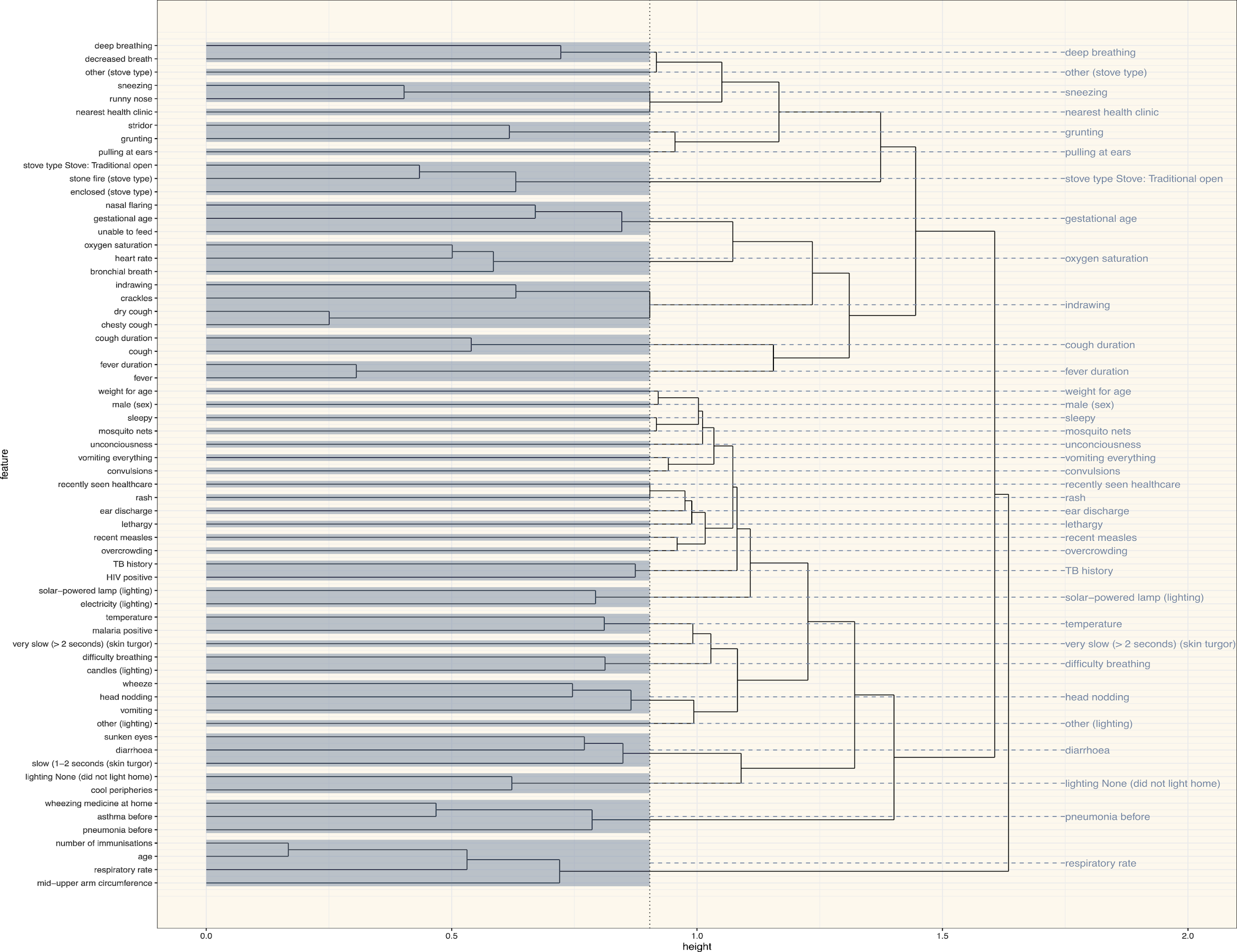


**Figure E: Clustering dendrogram of BIOTOPE features and representative members for each cluster**


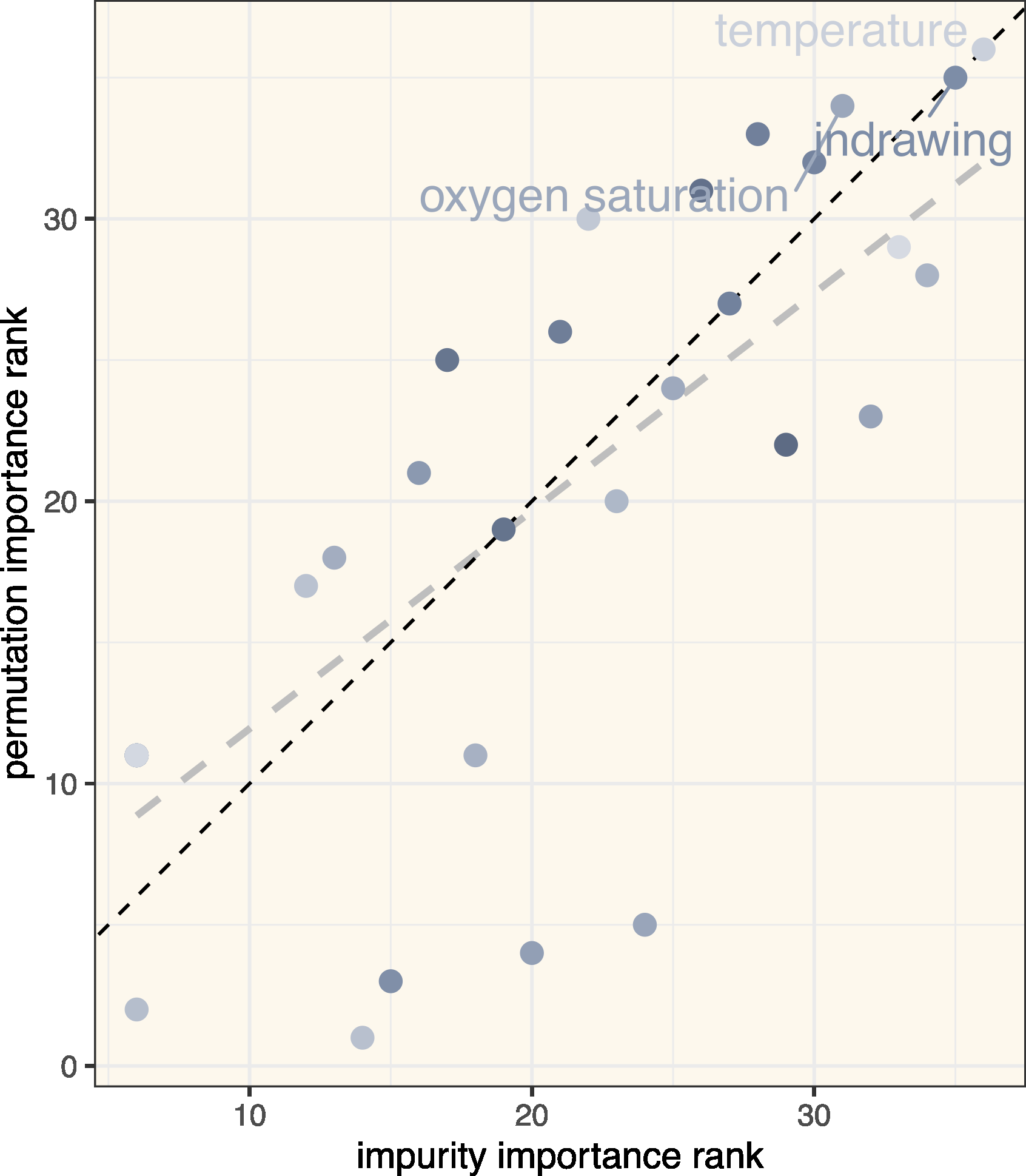


**Figure F: Correlation between impurity-based importance and permutation-based importance on BIOTOPE data**

# **e6: Effects of varying WHO danger sign representation in BIOTOPE data**


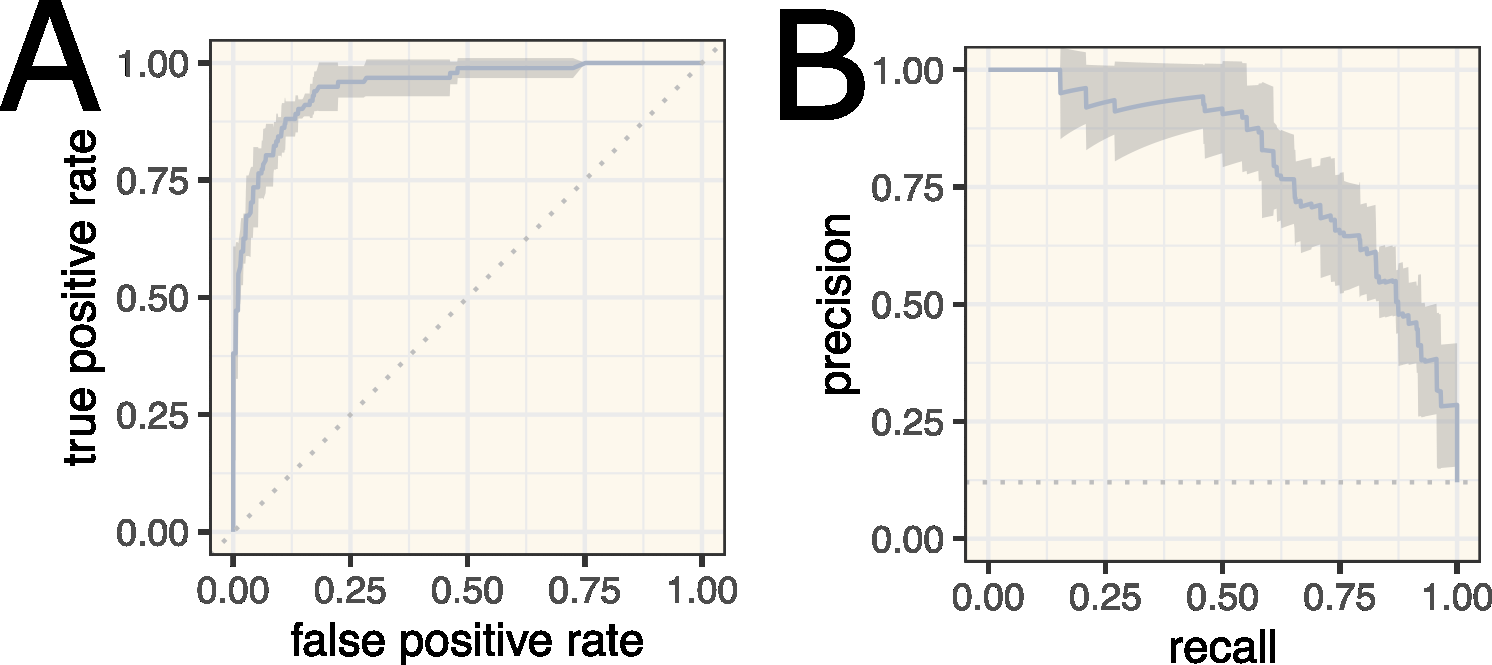


**Figure G: Performance of BIOTOPE random forest classifier when all participants with a WHO danger sign are removed (logistic regression intercept and slope: (0.26,1.07)) (logistic regression parameters correspond to the logistic regression model fitted using the severity status as the response variable and log odds of the calibrated probability as the predictor variable)**


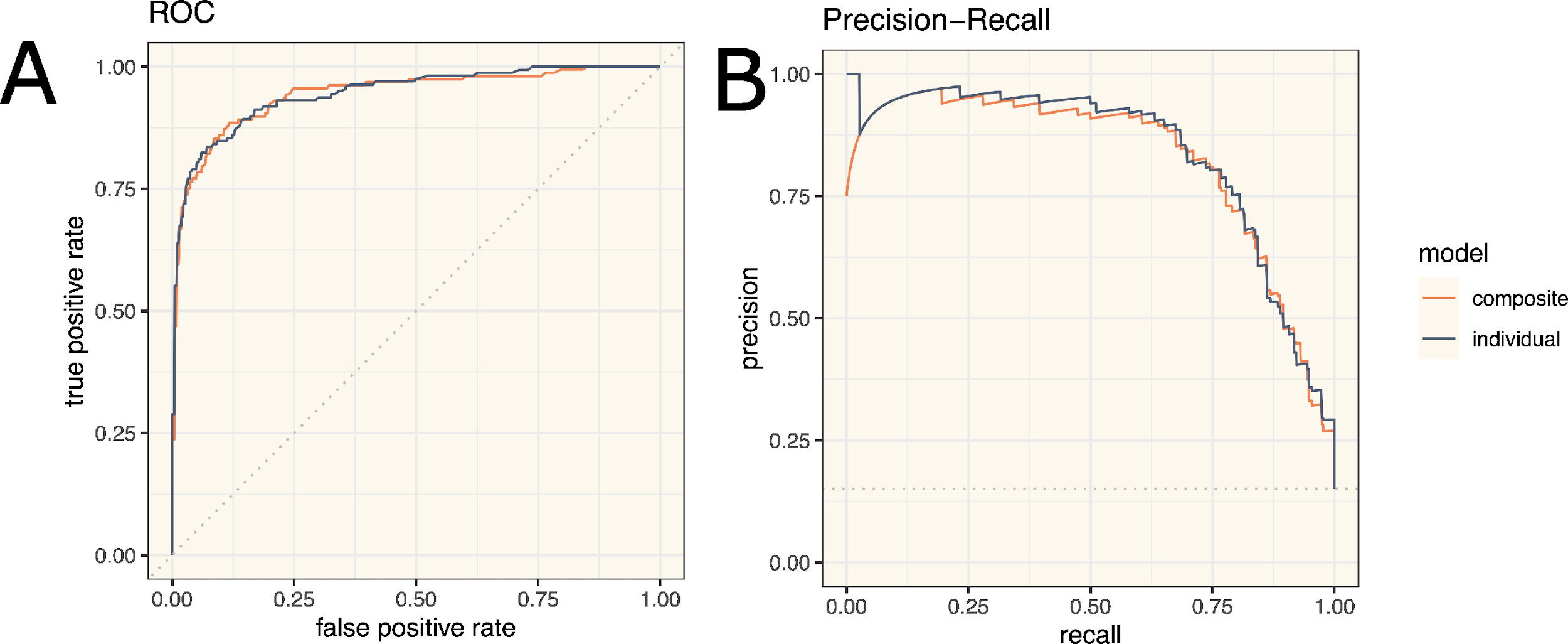


**Figure H: Performance of BIOTOPE random forest classifier for different WHO danger sign representations (composite logistic regression intercept and slope: (0.26,1.12), individual logistic regression intercept and slope: (0.35,1.11)) (logistic regression parameters correspond to the logistic regression model fitted using the severity status as the response variable and log odds of the calibrated probability as the predictor variable)**

*composite WHO danger sign: The presence of one or more WHO danger signs is treated as a single variable*

*individual WHO danger sign variables: Each danger sign is treated as an individual variable*

# **e7: Machine learning glossary**

| Data preprocessing | Data preprocessing is the process of preparing data for machine learning models. This is the first step in creating a machine learning model. For instance in this paper it included defining the outcome and removing participant and feature data that had more than 50% of data missing. |
| --- | --- |
| Hyperparameters | Hyperparameters correspond to metaparameters that define the parameter architecture (e.g. number of parameters) without specifying the values of the parameters comprising the architecture. |
| Hyperparameter optimisation | This is the process of identifying the optimal set of hyperparameters for a learning algorithm. |
| Parameter | Hyperparameters are chosen before training begins.The learning algorithm attempts to learn the optimal parameter values given the hyperparameterisation. Parameter values are continuously updated during learning. At the end of the training process, the final parameter values define the model. |
| *k*-fold cross-validation approach | *k*-fold cross-validation is a technique for evaluating predictive models. The dataset is divided into *k* subsets or folds. For each fold, the model is evaluated on the *k*th fold after having been trained on the remaining *k*-1 folds.. Performance metrics from each fold can be aggregated to estimate the model's generalisation performance and estimate uncertainty in that estimation. |
| Support vector machines | Support Vector Machines (SVMs) are a class of supervised machine learning algorithms used for classification and regression tasks. These algorithms typically seek to find a hyperplane that separates observations from different classes such that the distance from observations of both classes to the hyperplane are maximised. Often, to achieve such separation, the original data is mapped into a higher-dimensional space. Predictions are made according to the equation of the hyperplane. |
| Naive Bayes | Naive Bayes is a probabilistic classifier based on Bayes' theorem. Specifically, observations are assigned to the class which maximises the joint probability distribution of class label and predictor variables. For this method, predictors are assumed to be conditionally independent given the class label. |
| Neural network | Neural networks belong to the class of nature-inspired computing algorithms. Specifically, an artificial neural network computational model is inspired by the architecture and mechanics of biological neural networksNeural networks consist of interconnected nodes (or neurons) organised into layers. Predictions are obtained by propagating input data sequentially through each layer via learned parameters. |
| Random forest | The random forest approach pertains to a technique used in machine learning for both classification and regression tasks based on decision tree learning. The random forest method expands on decision tree learning by combining many decision trees to make predictions, where each tree is trained on a different subset of the training data and features. |
| Boruta feature selection | The Boruta feature selection algorithm is designed to identifies relevant features from a dataset by comparing the performance of the model when the feature’s values are randomly permuted across the observations versus the performance of the model when the feature’s values are held intact. For high-dimensional datasets, the number of features is large and some features may be irrelevant or redundant, which can lead to overfitting and increased computational complexity. Boruta feature selection can facilitate in diagnosing and remedying these issues. |
| Dummy encoding | Dummy encoding is a technique used in machine learning to convert categorical variables into a numerical format that can be used for machine learning algorithms. Specifically, each category within the categorical variable is reprocessed as a binary variable taking a value of 1 if, for a given observation, the observation belongs to that category and 0 otherwise. For certain algorithms, a designated “reference” class can be omitted for model identifiability. |
| Near-zero variance feature selection | Near-zero variance feature selection is a data preprocessing technique used in machine learning to identify and remove or flag features that exhibit narrow variance across observations in a dataset. Given the lack of variation in the data, near-zero variance features can be problematic for machine learning models as they often do not contribute meaningful information and may lead to overfitting or reduced model performance. Removing such features can lead to improvements in accuracy, robustness, parsimony and explainability of a given model. |
| Learning curve analysis | Learning curve analysis is an approach used in machine learning to assess the performance of a model as the amount of data available to the model increases. Specifically, it involves recording the model's performance metrics (such as the area under the curve) against the number of training examples. Learning curve analysis provides insights into a model's behaviour, including its ability to learn from data, detect overfitting or underfitting, and estimate the amount of data required for optimal performance. |
| ROC curve | A receiver operating characteristic (ROC) curve is a graphical representation typically used to evaluate the performance of binary classification models across different classification thresholds. Specifically, it plots the true positive rate (TPR) against the false positive rate (FPR) at a range of thresholds.  TPR, also known as sensitivity or recall, measures the number of positive instances (i.e. true positives) that are correctly identified by the model as a proportion of all positive instances.  FPR measures the proportion of negative instances (true negatives) that are incorrectly classified as positive by the model as a proportion of all negative instances. FPR focuses on the rate of false alarms or false positives generated by the model. |
| Precision recall curve | A precision-recall (PR) curve is a graphical representation typically used to evaluate the performance of binary classification models, particularly in situations where the classes are imbalanced. It plots the trade-off between precision and recall at different classification thresholds. Precision and recall are two important metrics in binary classification that measure different aspects of a model's performance.  Precision is a measure of the accuracy of the positive predictions made by the model.  Recall is a measure of the model's ability to identify all the positives in the dataset.  A higher precision indicates fewer false positives, while a higher recall indicates fewer false negatives. |

**Figure I: Machine learning glossary**

# **e8: Immunisation status of those included in BIOTOPE**

| name | 6<=age<12 | age>=12 | age>=6 |
| --- | --- | --- | --- |
| BCG (birth) | 457/459 (99.6%) | 1265/1269 (99.7%) | 1722/1728 (99.7%) |
| OPV (birth) | 459/459 (100%) | 1266/1269 (99.8%) | 1725/1728 (99.8%) |
| DPT-Hib-HepB (6 weeks) | 459/459 (100%) | 1268/1269 (99.9%) | 1727/1728 (99.9%) |
| PCV (6 weeks) | 459/459 (100%) | 1268/1269 (99.9%) | 1727/1728 (99.9%) |
| OPV (6 weeks) | 459/459 (100%) | 1268/1269 (99.9%) | 1727/1728 (99.9%) |
| Rota (6 weeks) | 459/459 (100%) | 1268/1269 (99.9%) | 1727/1728 (99.9%) |
| DPT-Hib-HepB (10 weeks) | 456/459 (99.3%) | 1267/1269 (99.8%) | 1723/1728 (99.7%) |
| OPV (10 weeks) | 457/459 (99.6%) | 1266/1269 (99.8%) | 1723/1728 (99.7%) |
| PCV (10 weeks) | 456/459 (99.3%) | 1266/1269 (99.8%) | 1722/1728 (99.7%) |
| Rota (10 weeks) | 457/459 (99.6%) | 1266/1269 (99.8%) | 1723/1728 (99.7%) |
| DPT-Hib-HepB (14 weeks) | 447/459 (97.4%) | 1264/1269 (99.6%) | 1711/1728 (99%) |
| PCV (14 weeks) | 446/459 (97.2%) | 1265/1269 (99.7%) | 1711/1728 (99%) |
| OPV (14 weeks) | 448/459 (97.6%) | 1265/1269 (99.7%) | 1713/1728 (99.1%) |
| IPV (14 weeks) | 439/459 (95.6%) | 1264/1269 (99.6%) | 1703/1728 (98.6%) |
| Measles (9 months) | 176/459 (38.3%) | 1242/1269 (97.9%) | 1418/1728 (82.1%) |
| Malaria | 26/459 (5.7%) | 197/1269 (15.5%) | 223/1728 (12.9%) |

**Table I: Immunisation status of those included BIOTOPE2 cohort**

# **e9: BIOTOPE study training schedule**

Two days of training for all research staff was undertaken covering the following topics. Site visits for monitoring and evaluation were undertaken every two weeks for the first month, every three weeks for the second month and every month thereafter.

Topics covered during training included

- Overview of the BIOTOPE 2 Study
- Pneumonia in Paediatrics (Signs and Symptoms, Causes, WHO classification of Pneumonia, WHO treatment guideline etc)
- Detailed sample collection procedures, Blood, Oropharyngeal and Nasopharyngeal swabs
- Child Protection policy/protocol
- Project Monitoring and Quality Check
- Clinical procedures to be involved in collection of data and Instruments to be involved and their operations
- Overview of the BIOTOPE Study (Methods and break down of responsibility)
- Laboratory procedures for FBC, MRDT, HIV RDT and Laboratory samples preparation for archiving for those to be shipped to Ireland
- An overview of bioethics including definition, principles, importance of considering ethics in Research, compensation is given to the participants and the implications
- Informed Consent in the BIOTOPE 2 Project
- Clinical Component of Case Report Form (CRF)
- Demographics and Laboratory component of CRF

# **e10: Public Involvement**


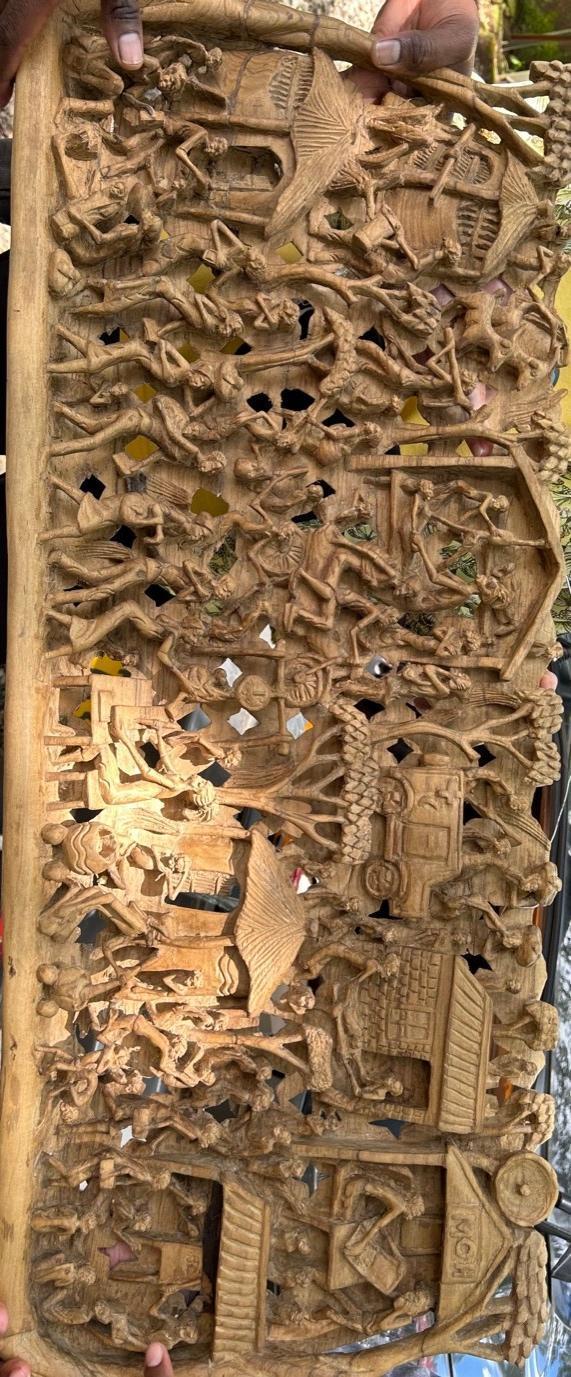


As part of the project, we conducted a needs assessment with local communities and health workers to ensure our solutions aligned with their needs. Collaborating with an artist from the Kungoni Centre in Mua, Malawi, we used the results from the needs assessment and the initial findings of the project to shape the plans further. The artist created a sculpture titled *"Life is Getting Better,"* which reflects the community’s vision of how the BIOTOPE project will bring positive changes. The sculpture tells the stories shared by parents, community leaders, and health workers about the anticipated impact of the project.

The image above shows the sculpture, and an explanation of its context is provided below.

A preprint outlining interviews is available at [A Human Factors Approach to Childhood Pneumonia A qualitative study in Malawian primary care](https://www.medrxiv.org/content/10.1101/2024.01.03.23300675v1) https://doi.org/10.1101/2024.01.03.23300675

**Every Kwacha Counts (Top right corner)** At the beginning of this story we see people busy trying to make a living with the resources they have. In Malawi 80% of people rely on the land for their livelihood. It is from the land that they get their daily food which is maize. The rainy season begins in November and continues until March and proves the busiest time for people. It is also the time when sickness has more of an impact. People often only have one meal at this time of the year as their food reserves are depleted and yet it is the time when they engage in a lot of hard physical work. Such a combination promotes various types of illness especially malaria and respiratory problems. When the rain finishes people still try to maintain gardens near rivers or damp places. The artist shows a man with his watering can pouring water on his plants which he will sell in the local market. The area of Mua has been blessed over the years form the generosity of wellwishers who have built irrigation schemes which allows people to cultivate for most of the year. People have a lot of plants and fruit which can boost their health but a lot is sold to cover school fees and medical costs. In this scene there are also some goats which are common animals for rearing in the area together with fowl and some cows. Meat is a luxury, and animals are reared for sale to help in challenging times such as medical emergencies or to buy fertiliser for their fields.

**We are all sick together (Bottom left)** The next scene reminds us the there is little understanding of the nuclear family in Malawi. Any event whether good (weddings, birth of child) or bad (funeral, sickness) involves the extended family both from the mother and fathers side. It is support system that bears much fruit especially when the unexpected sickness of a child happens. Here we see the family discussing how they will proceed, and as always money is a big deciding factor. Modern technology has greatly assisted families in their decision making. Here we see the father using his phone to contact other family members asking for assistance. Life is also made easier by the many means by which people can transfer money, easing the pressure financially.

**Beginning the journey in the health system. (Bottom centre)** The first port of call in the child’s medical journey is what is locally called “scalu.” This word is a Chewa pronunciation of the English word “scale”, which is what is used to weigh the child. It is here that the parents get the first indications about the health of their child. Traditionally it was the domain of women but as our carver presents there is now an insistence that men are also expected to accompany their partners. That is why we see both parents walking to the local place where children are received. One of the fathers is carrying his child on the back which also shows changing attitudes to the father’s role in caring for his child. They also move with their phone so they can keep the family at home updated as to the progress of the illness. The child is shown on the scale being weighed and this is one of the first indicators of malnutrition and impending illness. It is here that the “red or blue book (health passport)” – the health passport is used for recording purposes and will be the main reference for other interventions in the health of the child. Minor assistance may be given at this stage if the child is not seriously ill. The woman at the desk, who receives the parents is someone who has received further education and is aware of what signs are to be noted in terms of sickness. Over the years in Malawi there has been increasing support to provide opportunities for the education of the girl child and preventing early marriages. Education is seen as one of the key tools in promoting women in Malawi. The artist also depicts this woman with a modern hairstyle which shows she has been outside the village influences during her studies. The hospitals we meet on the way Modern technology has meant that people are becoming more aware of health issues and the facilities that are available. For some they have access to television, others listen to the radio while other information will be sent through the mobile phone, as was the case for Covid-19. For that reason, the parents of a sick child are aware of the need for a quick medical attention. 1 At the centre of this panel the carver shows the parents using a motor bike to bring their sick child to the district hospital. It is more expensive to hire a motorbike but it ensures the child will be taken care of quickly. In the top middle scene, the child is connected up to a drip and basic medical attention can be provided. Since mobile network is widespread it means that communication between the district and general hospital is vastly improved. If the medical staff need advice they can contact their central hospital who would have more qualified staff who would advise a referral. It also means that an ambulance can be swiftly sent to transport the sick child. The ambulance carries two symbols the cross and the crescent, as the artist reminds us that access to medical assistance should hold no discrimination. We see the ambulance arriving and the stretcher taking the sick child into the hospital. It is here that any additional care can be given to help the child. It is at the hospital that modern technology is used to provide the best medical assistance possible.

**Modern Communication** Due to various types of communication and technology there is increasing means of improving the health services provided here in Malawi. On the right-top corner we have the offices of the Ministry of Health (MOH). It is here that information related to health services, the spread of disease and current medical activities are sent and correlated. Computers in all their formats help to ensure that this information is organised well and put to good use. The satellite dish makes sure that sharing of information is not confined to Malawi but involves international cooperation. Such sharing of information ensures that the medical services in Malawi continually progress. Children are one of the main beneficiaries of this progress

**The Future is Bright (lower right corner**) We find the fruits of a good health system and collaboration. The young child has now fully recovered and has returned to the family. Food is being cooked and a sense of peace has returned to the household. There is a new hope that the future for this child will be better than what the parents had to endure. The artists depict the child attending school, learning ABC from the blackboard. There is great enthusiasm for education in Malawi and it is seen as the best future to offer a child. However, paying for school fees becomes an increasingly difficult prospect if resources are used for medical expenses. If the child is healthy and happy then it provides greater opportunities for planning into the future. As with technological advances in medicine so too there is hope that education will also benefit from these changes.

# **e11: Link to Github repository**

<https://github.com/BIOTOPEproject>

# **e12: Tripod AI checklist**

**

**

**
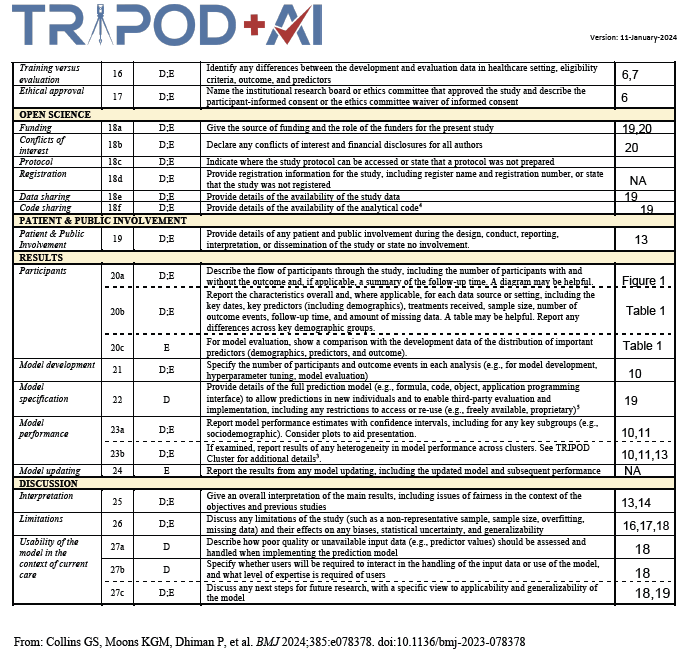
**

# **e13 Discrimination of existing childhood pneumonia risk scores vs. BIOTOPE**

| **Method / score** | **Setting** | **Outcome Predicted** | **Reported AUC ROC** |
| --- | --- | --- | --- |
| **PERCH severity score** | Hospitalised children with severe/very severe pneumonia (multi-country cohort) | In-hospital mortality | 0.84 (1) |
|  | Hospitalised children in the PREPARE pooled dataset (multi-country studies) | In hospital pneumonia related mortality | 0.55 (95% CI 0.37–0.73)(2) |
| **PREPARE risk assessment tool** | Hospitalised children in the PREPARE pooled dataset (multi-country studies) | In hospital pneumonia related mortality | 0.83 (95% CI 0.81–0.84)(2) |
| **RISC (HIV-negative)** | Hospitalised children in the PREPARE pooled dataset (multi-country studies) | Hospitalised pneumonia-related mortality | 0.66 (95% CI 0.58–0.73)(3) |
|  | Hospitalised children with severe/very severe pneumonia (multi-country cohort) | In-hospital mortality | 0.76(1) |
| **RISC-Malawi** | Hospitalised children in the PREPARE pooled dataset (multi-country studies) | Hospitalised pneumonia-related mortality | 0.75 (95% CI 0.74–0.77)(3) |
|  | Validation study in hospitalised children in Kenya | In hospital mortality | 0.77(4) |
| **WHO danger signs** | HIV−negative children aged 1–59 months enrolled in the Pneumonia Etiology Research for Child Health (PERCH) study with severe or very severe pneumonia in hospital | In-hospital mortality | Number of danger signs 0.82(1) |
| BIOTOPE cohort (Current study) | Children presenting in primary care with WHO defined pneumonia in Malawi | Hospitalisation and/or mortality within 7 days | 0.87 |

1. Gallagher KE, Knoll MD, Prosperi C, Baggett HC, Brooks WA, Feikin DR, et al. The Predictive Performance of a Pneumonia Severity Score in Human Immunodeficiency Virus-negative Children Presenting to Hospital in 7 Low- and Middle-income Countries. Clin Infect Dis Off Publ Infect Dis Soc Am. 2020 Mar 3;70(6):1050–7.

2. Rees CA, Colbourn T, Hooli S, King C, Lufesi N, McCollum ED, et al. Derivation and validation of a novel risk assessment tool to identify children aged 2-59 months at risk of hospitalised pneumonia-related mortality in 20 countries. BMJ Glob Health. 2022 Apr;7(4):e008143.

3. Rees CA, Hooli S, King C, McCollum ED, Colbourn T, Lufesi N, et al. External validation of the RISC, RISC-Malawi, and PERCH clinical prediction rules to identify risk of death in children hospitalized with pneumonia. J Glob Health. 2021 Oct 9;11:04062.

4. Ogero M, Ndiritu J, Sarguta R, Tuti T, Akech S. Pediatric prognostic models predicting inhospital child mortality in resource‐limited settings: An external validation study. Health Sci Rep. 2023 Aug;6(8):e1433.

# **e14 Overview of the Integrated Community Health Information System (iCHIS) in Malawi**

The Integrated Community Health Information System (iCHIS) is a Ministry of Health–owned digital platform designed to support routine data collection, clinical decision support, supervision, and reporting at the community level in Malawi. Developed on the DHIS2 Tracker architecture, iCHIS replaces fragmented, paper-based and program-specific tools with a unified system that integrates household registration, individual longitudinal records, disease surveillance, service delivery, supervision, and reporting within a single interoperable platform. The system is primarily used by Health Surveillance Assistants (HSAs) via Android tablets in offline mode, with synchronization occurring when connectivity is available, and by supervisors and managers through a web-based interface for monitoring and decision-making

Deployment of iCHIS is following a phased national rollout strategy led by the Ministry of Health in collaboration with multiple implementing partners. Rollout includes district readiness assessments, digital literacy training, training of trainers, supervised field practice, and ongoing technical support. As of2025, iCHIS has been deployed with partial or full coverage in approximately 14 districts, reaching about 19% of the national community health workforce and serving over four million individuals. Of the planned system modules, eleven have been developed, including foundational registers (community, household, and person), integrated community case management, maternal and neonatal care, immunization, non-communicable diseases, reporting, supervision, and electronic disease surveillance. Deployment intensity varies by district depending on partner support, infrastructure readiness, and availability of devices and supervision capacity

In routine use, iCHIS supports point-of-care service delivery, real-time clinical guidance, and longitudinal tracking of individuals and households. Overall, iCHIS represents a maturing national digital health platform that has substantially improved integration and availability of community-level data, while continuing to face systemic constraints commonly seen in large-scale digital health implementations. It continues to be the single IT enabled community health information system in Malawi
