# Supplementary material for: Prediction of hospitalisation in young children with pneumonia in Malawi: A machine learning-based approach
Source: PLoS Med. 2026 Jun 9;23(6):e1005122. doi: 10.1371/journal.pmed.1005122 (PMC13271506; doi:10.1371/journal.pmed.1005122)
Supplement: Attachment — Submitted filename: Response to reviewers.docx [file pmed.1005122.s005.docx]

**Response to Reviewers:**

**Comments from the academic editor:**

**1.**	**I don't think the authors address how and why this will facilitate a better prediction tool that is implementable, either through better feature selection or weighting. I am not sure the author's were trying to answer the questions from that perspective, but I it is imperative that a prediction tool that is not useable in the intended context is not that helpful even if you can demonstrate better performance. Developing a model that can also be used in routine practice or one that demonstrates some new key insight I think is needed for broader impact.**

We agree that practical implementation is key. Therefore in choosing variables in this model, we have aligned them with the variables being collected by the iCHIS (integrated Community Health Information System) being implemented by the Malawi Ministry of Health. This allows a much broader range of variables to be used as it collects data not just on childhood illness but also on household composition and community health information. By using data collected from a number of sources the iCHIS system allows us to include a number of variables that are not traditionally collected in clinical datasets.

We have added the following to the discussion to highlight this:

*Although the model contains more variables than traditionally used in a risk score, it does align with the variables collected in the iCHIS system as developed by the Malawi Ministry of Health. As such, the model can be deployed in a real world setting. Work is currently underway to integrate this score directly into the iCHIS system.*

We have added the following to the methods:

*A critical challenge in prediction model development is ensuring that tools are implementable in the settings for which they are designed. Although our model includes a larger set of variables than traditional risk scores, these have been intentionally aligned with the data collected by the Integrated Community Health Information System (iCHIS) being implemented by the Malawi Ministry of Health. This alignment ensures that the model can be deployed within existing community health workflows without adding extra data collection burden. Moreover, by drawing on iCHIS data—including household and community-level indicators in addition to clinical features—the model has the potential to leverage a richer feature set, allowing improved weighting of predictors and potentially more accurate risk stratification. Importantly, integration within iCHIS also facilitates sustainability: the model can be updated and retrained as new data accumulate, maintaining its relevance across changing epidemiological and health system contexts.*

We have added the following to the conclusion:

*Embedding the tool within iCHIS will not only enhance its immediate usability in Malawi but also provides a framework for scalable adaptation to other geographic settings. In doing so, this approach moves beyond demonstrating predictive performance of machine learning tools to creating a tool that is both clinically relevant and sustainable in real-world practice. Further work is ongoing to implement this.*

**2.**	**I worry that some of their findings are driven by prediction only within their dataset (any scoring tool developed elsewhere likely to perform worse) and the number of variables they have in their dataset. The most important variables still seem to be the ones that would be expected and are likely in the other risk tools.**

We appreciate the concern and note that this point is addressed in more detail in response to the points raised in the review below. We have provided a demonstration that even when restricted to the same variables used for other scores, the use of a machine learning framework leads to significant improvements in prediction. Training on one dataset and evaluating on a dataset collected at a different time point and in different clinics to the original training dataset enabled us to evaluate the extent to which the prediction is affected by dataset shift and, thus, the likelihood that our models, trained at one time point, could be used for prediction at a subsequent timepoint in the Malawi context. Indeed a broader validation is currently underway using different seasons and locations to highlight this further. However even without training and testing of this algorithm in different sites and over different time periods, we demonstrate the potential value of the model as a practical tool within Malawi, where childhood pneumonia is a significant public health problem. A practical implementation of this is currently being investigated as the initial use case. Application in other healthcare systems is envisaged in the future, however, in order to demonstrate that a model trained in one setting can be applied in another, generation of data comparable to BIOTOPE in other settings will most likely be required to provide a basis to train models for that system. We feel that presence of some of the most important variables identified in other risk scores provides reassurance that the algorithm is clinically appropriate. However, the results also highlight other variables not traditionally included in risk scores (Figure 3) such as gestational age, distance from health facility and type of stove, showing the importance of these factors. Some of these variables also align with the recent WHO research priorities on areas such as prematurity (Guideline on management of pneumonia and diarrhoea in children up to 10 years of age World Health Organisation 2024).

**3.**	**I was not totally clear, but it does seem that the authors developed models that only used the features used in other commonly used prediction tools (that are presumably applied in actual practice). I think this is important if that is the case, and in many ways those comparisons might be the most important (i.e., demonstrating that applying machine learning to the same variables that are commonly available still improves performance). However, the reviewers also raise important points as to whether those comparisons are even appropriate given the difference of setting (outpatient vs already hospitalized).**

We have applied our model separately using only the variables that have been included in other tools as well as with a larger collection of variables collected in the BIOTOPE studies. This is discussed in greater detail in response to the relevant reviewer comments. We show that we achieve an improvement in performance by applying machine learning to the same set of variables that have been used by other tools and a further improvement in performance with a machine learning model trained on all of the variables we collected which are aligned with the iCHIS system being implemented in Malawi. While it is true that the other tools have been developed for use in the hospital setting, in practice these tools are used to assess severity. Our work demonstrates that these tools are sub-optimal for the critical application of evaluating the severity of pneumonia in the outpatient setting and particularly primary care where most patients present. Our intention is not to critique the existing tools within their intended use context, but to demonstrate that a machine learning model applied to the same variables and to additional variables that we have collected can far exceed their performance within the outpatient setting, which is very relevant for case management in under-resourced healthcare settings. Also by using variables which are already being collected in a national electronic health system (iCHIS) we can utilise a broader range of variables than would be possible if just collecting clinical data and that this broader range of variables have benefit in predicting severity. We have shown that the existing tools do not perform as well for this application.

**REVIEWER COMMENTS**

**Reviewer #1:**

**The paper applied machine learning (ML) models to predict the hospitalization and/or death within 7 days after initial presentation in 2-59-month-old Malawian children with pneumonia. The features of the ML models included demographic, assessment finding, vital sign, HIV, lifestyle data. The data were further analyzed using impurity-based and permutation-based feature importance analysis, learning curve analysis, mutual information analysis, and correlation analysis. The suggestions for revising the paper are listed below.**

**1.	In the "Study population" section of methods, the authors have mentioned that the 2016 BIOTOPE cohort was used as testing dataset, while the 2022-2023 cohort used as the training dataset. It would be helpful if the authors to clarify the reason for this choice, as it is more common to use older datasets for model training, and more recent datasets for testing, in order to simulate a prospective validation. Could the author clarify**?

This choice was made because Biotope (2022/23 cohort) was larger than Biotope (2016 cohort) and, typically, the larger dataset is selected for training while the smaller dataset is used for model evaluation in machine learning.

The following sentence has been added to the Study Population subsection of Methods:

*Although collected at a later date, the BIOTOPE training cohort was used for training purposes due to its comprising a larger number of participants.*

**2.	In this study, one of the predicted outcomes—death—is represented by only a single case within the entire cohort, which is included in the training set, with no corresponding cases in the testing set. Given that machine learning approaches are inherently data-driven and rely on sufficient representation to learn meaningful patterns, a single instance is insufficient to develop a robust or generalizable predictive model. This extreme class imbalance renders the outcome unreliable for model evaluation. I recommend that the authors either redefine this outcome or consider excluding it from the analysis due to the lack of representative data.**

Hospitalisation and/or death constitute a composite variable outcome and this outcome was a pre-specified endpoint. We do not attempt to predict death as an outcome on its own. Removing the single individual who died from this composite outcome would have minimal impact on the results, given that it is a single observation. Given that this outcome was defined prior to the study start, we feel that it is appropriate to retain it in its current form and highlight in the Discussion that the death component of this outcome is represented by a single case.

We have added the following to the discussion:

*Death is a more objective representation but is a rare outcome in primary care cohorts with only one death in our cohort. However, in primary care, one of the main objectives is to appropriately identify those who require hospitalisation. As such, we consider that this is a useful initial outcome measure. Further studies validating this–and alternative–score(s) should be conducted.*

**3.	Age-related variability is an important consideration in paediatric populations, as normal ranges for vital signs vary with development stage. For example, the respiratory and heart rate are typically higher in infants and gradually increases and stabilize with age. Could the authors clarify whether age-based normalization was applied to features such as respiratory rate and heart rate data before inputting them into the machine learning models**?

The selected random forest classifier is capable of accommodating interactions between predictor variables, such as the interaction between age and heart rate or age and respiratory rate. In other words, the model can, for example, allow the effect of heart rate on the predicted outcome to be different for patients in different age groups. Therefore age based normalisation is not necessary prior to inputting them into the machine learning models.

**4.	In the "Feature importance analysis" section of methods, the authors have described those two distinct approaches for evaluating feature importance were applied: (1) impurity-based approach and (2) a permutation-based. It is hard to understand (what calculations or implementation details were applied?). Could the authors clarify how these methods were applied in this study? Including relevant equations or methodological steps would be helpful for reader understanding.**

We thank the reviewer for the suggestion and have provided additional information on the variable selection methods in the revised manuscript.

The Feature importance analysis section in the methods now reads:

*We assessed feature importance using two distinct approaches: (1) a feature impurity-based approach, whereby features are progressively graded in importance as a result of how “pure” tree branches become (i.e. the heterogeneity of a branch with respect to the class outcome) as a result of splitting the data on that feature across all trees considered by the random forest*[*^14^*](https://www.zotero.org/google-docs/?Lv1YZZ) *and (2) a permutation-based approach which computes importance as a function of the change in classification performance of the model resulting from using a permuted feature (i.e. randomly reassigning a feature’s values to patients such that any signal contained within the feature with respect to class outcome is perturbed) relative to the original unpermuted feature. We performed permutation-based feature importance after cluster-based feature selection as per the description above. Feature importance analysis was only performed on the BIOTOPE training cohort data; furthermore, derived importances were not used in any downstream analyses.*

**5.	In Table S2, the authors provide an overview of features used in training model. It may be helpful to include the features used in existing models such as RISC, PERCH, and PREPARE for comparison? Additionally, providing the missing rates for BIOTOPE cohort in this table, would help clarify whether model performance may have been influenced by missing data.**

We thank the reviewer for these suggestions. The features used in existing models have been added to Table S2 in the supplemental file. Missing data rates have also been added to the supplemental file.

**6.	The three figures, Figure 1 to 3, need to have better resolution. In Figure 3, the text is too small to read; please enlarge the font size or adjust the layout to improve readability.**

Higher resolution images have been uploaded with this revised version.

7.	**Some of the related ML studies in pediatric pneumonia prediction may be included in the manuscript:**

*****	[**(1)**](https://xmed.jmir.org/2025/1/e57719/)

*****	[**https://medinform.jmir.org/2022/1/e28934**](https://medinform.jmir.org/2022/1/e28934)

*****	[**https://onlinelibrary.wiley.com/doi/abs/10.1002/ppul.26897**](https://onlinelibrary.wiley.com/doi/abs/10.1002/ppul.26897)

We thank the reviewer for providing these additional resources which have been added to the introduction.

**Reviewer #2:**

**I commend the author on their ongoing work to reduce pneumonia related mortality in children living in low- and middle-income countries.**

**1.**	**My recommendation would be to include RISC-Malawi (doi: 10.1371/journal.pone.0168126 as part of the analysis). This is important as (1) its the only published tool studying the same population and (2) when externally validated by the PREPARE group it outperformed all other clinical prediction tools including RISC and PERCH (doi: 10.7189/jogh.11.04062) making it the best performing pneumonia-related mortality clinical prediction tool that**

We thank the reviewer for this suggestion and note that the RISC-Malawi method is now included as a comparative model and is discussed throughout the main manuscript; however, we note that the derived random forest approach still outperforms all compared approaches.

**2.**	**Though I am not an expert in machine learning and hope the manuscript will undergo evaluation by a technical expert in data science to ensure the methods given the counts of outcome are appropriate, I commend the authors on their ingenuity. The ML algorithm was designed to identify children with WHO defined pneumonia, diagnosed in outpatient settings in rural Malawi, at risk for hospitalization or death. I am concerned about the adequacy of sample size to determine this.**

**Inadequate number of deaths in the training (1 out of 1872 cases) and testing (0 out of 212 cases); why is death information missing for 181 and 244 cases in the different cohorts?**

**Hospitalization/death occurred in 14.3% of training cohort and 12.1% of testing cohort; yet WHO danger signs (an indication for hospitalization) was more prevalent in both cohorts at 17.6% and 15.9% respectively. So what is being described, in terms of hospitalization practices, may be (1) a deviation from national guidance or (2) change in clinical status or (3) reflect differences in interpretation of clinical status at time of presentation to the hospital.**

Thank you for this important point. Death information is missing for patients either due to a loss in follow-up or data recording failure. The manuscript does not attempt to predict death as an outcome on its own; instead, death and hospitalization are considered together as a composite outcome. Removing the single individual who died from this composite outcome would have minimal impact on the results, given that it is a single observation. Given that this outcome was defined prior to the study start, we feel that it is appropriate to retain it in its current form and highlight in the Discussion that the death component of this outcome is represented by a single case.

We have added the following to the discussion

*Death is a more objective representation but is a rare outcome in primary care cohorts with only one death in our cohort. However, in primary care, one of the main objectives is to appropriately identify those who require hospitalisation. As such, we consider that this is a useful initial outcome measure. Further studies validating this–and alternative–score(s) should be conducted.*

We also note in the discussion that, *“We used a marker of severity defined by hospitalisation and/or death within seven days of assessment. Study participants may have been hospitalised for reasons other than severe pneumonia and hospitalisation is a subjective decision dependent on a number of factors relating to the health system, health worker and family”*.

We feel that the complex nature of the relationship between WHO danger signs and hospitalisation justifies the inclusion of WHO danger signs as a predictor covariate ( and is further discussed in 3. below). We accept that hospitalisation is a complex decision influenced by a number of factors which are non clinical as highlighted by the reviewer. However, we do believe it is a legitimate outcome measure particularly in primary care where this study is based – particularly given that one of the main decisions is whether to escalate care or not. From a health systems perspective, hospital admission also directly reflects economic and logistical burden. Hospitalisation frequently represents the threshold at which significant healthcare costs are incurred.

**3.**	**Since WHO danger signs were evaluated as part of the model but they also define the indication for the outcome of interest, I'm unclear if this is a methodologically sound approach.**

While WHO danger signs are indeed an indication for hospitalisation, their inclusion in our model reflects the pragmatic nature of the study. In practice, hospitalisation decisions were not based solely on WHO criteria. Clinicians exercised their own judgement, and the admitting clinician was different from the referring primary care clinician. This meant that individual interpretation and contextual factors influenced the decision to admit.

This is supported by our data: although WHO danger signs were present in 17.6% of the training cohort and 15.9% of the testing cohort, actual hospitalisation or death occurred less frequently (14.3% and 12.1% respectively). This discrepancy highlights both the variability in clinical decision-making and the imperfect alignment between guideline criteria and real-world outcomes, which has also been observed in other studies.

Therefore, we believe including WHO danger signs in the model is methodologically sound, as it captures a clinically relevant and widely used set of variables, while recognising that hospitalisation decisions are ultimately multifactorial. We have also undertaken an analysis of the BIOTOPE algorithm in those children without a WHO danger sign showing good performance also. In this case the BIOTOPE random forest model (excluding children who had a WHO danger sign) had an AUC of 0.95 and a PRAUC of 0.79. We have added a 2x2 table to the supplemental file to convey this information also.

**4.**	**The prediction tools compared (RISC, PERCH, and PREPARE) were designed to be used in hospitalized/emergency center patients (who were then hospitalized), not children evaluated in outpatient settings. They were designed to predict death - whereas the BIOTOPE tool did not include enough subjects who died to be able to distinguish those who died vs survived their illness. Though I understand the desire to compare these tools, its very important that these distinctions are clear and adequately discussed.**

We agree with this and have added the following to the limitations section:

*The prediction tools used for comparison (RISC, RISC-Malawi, PERCH and PREPARE) were originally developed and validated in hospital populations, rather than in children presenting in outpatient settings. In addition, these tools were designed to predict mortality, whereas the BIOTOPE dataset contained too few deaths to reliably distinguish between children who died and those who survived. Nevertheless, comparison with these established tools is still important, as they represent the current benchmarks in paediatric pneumonia risk stratification. Evaluating their performance in a primary care population provides valuable insight into their generalizability and highlights the potential added value of the BIOTOPE tool in broader clinical contexts.*

**5.**	**I wish there was more discussion about the actual applicability of the developed tool in outpatient settings. The paragraph on the development of continuous tools didn't sound applicable as inherently children evaluated in outpatient settings are not continuously monitored.**

We apologise for the lack of clarity in this section. Our reference to “continuous” tools was not intended to imply continuous patient monitoring, which we agree is not feasible in outpatient or primary care settings. Rather, we were referring to continuous retraining of the model, where the algorithm is periodically updated as new data are received.

We have included this section below to make this clearer:

Compared to existing risk scores, we have implemented an approach that can be continuously retrained and redeployed using machine learning. This does not involve continuous monitoring of individual patients. Instead, it refers to using routinely collected healthcare data, as it becomes available, to track model performance and retrain the algorithm when necessary. In this way, the model can adapt to changes such as seasonality or regional variation without requiring a complete offline redevelopment process. In practice, the algorithm can monitor its own accuracy and, if performance deteriorates, either update itself to restore accuracy or flag the need for review by an external panel. A robust AI governance framework is essential to ensure that updates are not triggered by trivial fluctuations, while significant shifts (e.g. emergence of new pathogens) are carefully investigated and understood. This approach facilitates the integration of models into routine healthcare data streams, supporting their ongoing clinical relevance and reliability.

We have also been more explicit about how the features included are based on the iCHIS system being implemented in Malawi throughout the manuscript.

**Overall I commend the author's ingenuity but recommend a closer look at if the tool they developed is methodologically sound, should be compared to hospital mortality tools, and, if they choose to compare their tool to hospital mortality tools they should be comparing it to RISC-Malawi as it was derived from a Malawian population hospitalized for pneumonia.**

We thank the reviewer for their kind words and note that we have now incorporated RISC-Malawi into the collection of comparative models.

**REVIEWER #3:**

**This manuscript describes the development and validation of a machine learning-based classifier to predict hospitalization/mortality within 7 days after initial presentation, for Malawian children with pneumonia aged between 2 and 59 months (median about 16 months). A training cohort (BIOTOPE) of 2053 children was collected between 2022 and 2023, and the test cohort of 456 children in 2016. A random forest classifier trained on all BIOTOPE features was found to achieve an AUC of 0.87 on the test dataset, significantly better than existing risk scores (PERCH, PREPARE, RISC, WHO severity criteria)**

**While the findings are encouraging, some issues might be considered:**

**1. In the Abstract, "Bakcground" might be "Background".**

We thank the reviewer for bringing this to our attention. This has been corrected.

**2. In the Data preprocessing subsection, it is stated that a patient is excluded if more than 50% of their data is missing, and that missing values were imputed. If possible, the percentage of missing data for each feature might be stated in supplementary material**.

We thank the reviewer for this comment and note that a table conveying missingness has been added to Table 1 in the main manuscript.

**3. In the Model Selection subsection, it is stated that four classifier families (SVM, naive Bayes, neural network, random forest) were explored, moreover with four different feature subsets. The full set of results for all these combinations should be presented in the supplementary material. Currently, it appears that only the best result for each model is shown (eTable 1). Information as to how each feature subset performs is of practical interest, given that some of the BIOTOPE features might not be available/collected in other studies or scenarios.**

We thank the reviewer for this comment and note that results for all explored combinations have been added to the supplemental file.

**4. In the Model Selection subsection, it is stated that for the random forest model in particular, the number of trees was fixed at 1000, and that each tree was grown until maximal purity with (again) fixed parameters based on n_features and n_observations. However, random forests are known to have a number of other possible hyperparameters, such as the number of features considered when splitting a node, the maximum depth of each tree, the maximum number of leaf nodes in each tree, etc. As such, it should be explained in greater detail as to what hyperparameterisation was actually performed for random forests (eTable 1 suggests none), and if not, why those common hyperparameters were not explored (especially as these hyperparameters appear to be available in boruta_py.py in the GitHub repo).**

We thank the reviewer for bringing this to our attention. Several hyperparameters were indeed explored. More detail is provided in the revised manuscript on the Random Forest hyperparameters that were tuned and how this was achieved.

We have added the following section to the Model Selection section of Methods:

*… a random forest family (*$n_{estimators}\times criterion\times n_{maximum features}$*;* $n_{estimators}\in\{10,100,1000\}$*,* $criterion \in\{gini impurity, entropy\}$*,* $n_{maximum features} \in\{\sqrt{n_{features}}, {log}_{2}(n_{features})\}$*) where each tree was grown until maximal purity was achieved (each individual tree was trained on a randomly sampled-with-replacement sample of observations of size* $n_{observations}$ *(*$n_{estimators}, n_{features}$ *and* $n_{observations}$ *correspond to the total number of trees, features and observations respectively in the supplied dataset while* $n_{maximum features}$ *corresponds to the maximum number of features used to build an individual tree)).*

**5. In the Results section, it is stated that the model was evaluated on a subset of patients without WHO danger signs. The significance of this exclusion might be explained further - what is special about the subset of patients without WHO danger signs? How are WHO danger signs defined?**

The WHO danger signs are defined by the World Health Organisation and are a set of clinical symptoms and signs which are designated to guide a health worker that a child should be hospitalised. There have been concerns that they have not functioned as well in identifying those who require hospitalisation in the recent past. However they are also subjective and, for instance, in this study, although WHO danger signs were present in 17.6% of the training cohort and 15.9% of the testing cohort, actual hospitalisation or death occurred less frequently (14.3% and 12.1% respectively). This discrepancy highlights both the variability in clinical decision-making and the imperfect alignment between guideline criteria and real-world outcomes, which has also been observed in other studies. However because the WHO danger signs are a criteria for admission we also undertook an analysis where children did not have danger signs and therefore did not have a current guideline criterion for admission. We felt that this allowed us to explore if the algorithm also worked well in patients without danger signs who, on a clinical basis, would require admission. In this case, the BIOTOPE random forest model (excluding children who had a WHO danger sign) had an AUC of 0.95 and a PRAUC of 0.79.

We have included the following in the results section:

*In order to determine if children hospitalised without a current criterion for admission based on the WHO danger signs could also be identified by the BIOTOPE algorithm, we evaluated the performance of the model in those without WHO danger signs (eFigure 7 and 8). In this case, the BIOTOPE random forest model (excluding children with a WHO danger sign) had an AUC of 0.95 and a PRAUC of 0.79.*

**6. In the Discussion section, it is stated that the WHO, RISC, PERCH and PREPARE models used only mortality (and not hospitalization) as an outcome measure. Given this the claim that the developed model is superior to those existing models may not be particularly valid, given the extremely low prevalence of death in the BIOTOPE cohort (1/2084). Notably, the exposition suggests only the difference in the input features used as affecting performance, while neglecting the difference in outcome definition. It is later acknowledged that death is a more objective but rare outcome, which may have affected performance for the other risk models.**

We agree with the reviewer that death is a more objective outcome measure, but in the context of primary care the key clinical decision is whether a child is at risk of deterioration and therefore requires hospital referral. Mortality is influenced not only by illness severity but also by downstream factors such as quality of hospital care, availability of medications, and access to oxygen, which vary across settings. For this reason, prior to study commencement we selected a pragmatic combined outcome of hospitalisation or death, as this is of direct relevance to health workers in primary care making referral decisions. We acknowledge that the difference in outcome definitions, in addition to the input features used, may partly explain the differences in model performance.

We have clarified this point in the Discussion section:

*The prediction tools used for comparison (RISC, RISC-Malawi, PERCH and PREPARE) were originally developed and validated in hospital populations, rather than in children presenting in outpatient settings. In addition, these tools were designed to predict mortality, whereas the BIOTOPE dataset contained too few deaths to reliably distinguish between children who died and those who survived. Nevertheless, comparison with these established tools is still important, as they represent the current benchmarks in paediatric pneumonia risk stratification. Evaluating their performance in a primary care population provides valuable insight into their generalizability and highlights the potential added value of the BIOTOPE tool in broader clinical contexts. Furthermore, the difference in outcome is clinically relevant: in primary care, the central question for health workers is not only whether a child will survive, but whether they are at risk of deterioration and therefore require referral for higher-level care. While mortality is a more objective endpoint, it is rare in outpatient cohorts such as BIOTOPE and is influenced by multiple health system factors beyond the initial clinical presentation. By contrast, hospitalisation is a pragmatic and actionable outcome that reflects real-world decision-making in primary care. Nevertheless, we recognise that both the outcome definition and the predictor features may affect model performance. This should be considered when comparing across models and requires further validation and clinical impact studies.*

**7. In the Discussion section, while dataset shift (and possible mitigation by continual learning) is discussed, the actual impact of the machine learning model is not explained - will treatment of the patient(s) differ depending on the diagnosis of the model? Moreover, if so, would it be expected that this would in turn also affect the performance and evaluation of the model? For example, the model might be very successful in flagging patients at risk of impending hospitalization or death. However, because of this predictive power, the patients receive appropriate care, and many of them therefore avoid hospitalization/death. In turn, since they no longer suffer hospitalization/death, the model then appears to be poorly predictive upon retrospective review. This might be further discussed.**

We thank the reviewer for raising this important issue. It is important to note that the model is not to replace clinical judgment but to act as a decision-support tool. In practice, the health worker would review the model’s prediction and either accept or reject the advice. This reduces the risk of a circular argument where the model simply predicts hospitalisation and thereby drives the outcome.

We have now addressed this point in the discussion:

*When the model is used to guide hospitalisation decisions, there is a risk that, during retraining, it may begin to learn institutional admission practices rather than true clinical severity. Moreover, by influencing patient management decisions, the model may inadvertently alter the data on which it is subsequently trained, thereby degrading its own performance. This creates a dependency between the model’s prediction (severity, defined by hospitalisation and/or death) and its recommended action (hospitalisation based on predicted severity).*

*To mitigate this circularity, future approaches could aim to decouple prediction from clinical action by redefining the target outcome as clinical deterioration rather than hospitalisation. Deterioration could be defined by objective indicators such as re-presentation to healthcare within a specified timeframe, prolonged hospital stay, or escalation to a higher level of care. Another potential safeguard would be to record clinicians’ intended management decisions before model input, enabling comparison between model predictions and independent clinical judgment, and allowing assessment of the model’s true added value. At scale, variation in climate, resources, and clinician training would also naturally introduce diversity in decision-making allowing monitoring of model performance and clinical outcomes, but continuous oversight by an AI governance committee would be essential to monitor these. Ultimately, clinical impact studies will be necessary to determine the model’s real-world effectiveness.*

8. **Given that it is stated that pneumonia is the single biggest cause of mortality in children under 5 years of age, the applicability of the random forest model to other datasets would be of interest. It should be highly recommended to validate the model on datasets from other sources/countries (possibly with a reduced set of features), if possible.**

We agree with the reviewer on this point. We are collecting data from other seasons and locations for a future study and also working with collaborators to validate the model on datasets from other countries and timer periods

**9. In Table 1, the denominator for the Death outcome is lower than that for the Hospitalization outcome (and the input features). This might be explained.**

Differences across denominators are explained by missingness in the data. In particular, data on deaths are missing for 181 patients in the training dataset and 244 patients in the testing dataset; there are no missing data for hospitalisation. Missing data do not contribute to the denominator counts; as a result, denominators reflect the number of complete instances for a given feature.

**10. From Table 2, (S13) risk score methods had AUC<=0.67 on the test set, compared to AUC=0.87 for the BIOTOPE random forest model. It might be commented as to whether these (relatively low) AUCs by PERCH/PREPARE/RISC/WHO are consistent with existing literature on their performance.**

We have now added a table to the supplemental file (S13) to show how these scores have performed on other datasets and also included the settings where the studies were conducted for comparison. We have also added to the discussion *An external validation study noted that although pneumonia risk scores have performed well among the cohorts in which they were derived, their performance diminished when externally applied.*

**11. In eFigure 1, the study sites are shown. Their status as training or test cohort might also be listed.**

We thank the reviewer for this suggestion. This suggestion has now been implemented in the supplemental file.

**12. For the list of input variables in S2, it might be considered to firstly format them as a table (with index numbers), and secondly to include columns for each of the models and subsets (BIOTOPE [x4 subsets from feature selection]/PERCH/PREPARE/RISC/WHO) indicating what features were available/used for each of these models.**

We thank the reviewer for this suggestion. This suggestion has now been implemented.

**Reviewer #4:**

**The manuscript describes a study to develop and evaluate a machine learning model to predict hospitalisation and/or death in childhood pneumonia using data from the BIOTOPE cohort study. The training and testing cohorts differ in location, with 9 primary care centres in Northern Malawi used for training and 2 used for testing, and time, with the training data from 2022/23 and testing data from 2016.**

**Note that I have expertise in applied machine learning, but not pneumonia, so my review predominantly focuses on the modeling aspect of the work.**

**Importance of the research question:**

**Pneumonia is the leading cause of death in children under 5 years globally. The authors aimed to develop a tool to improve risk stratification, which has the potential to improve patient outcomes by informing triage, referral and treatment decisions. Such a tool with the potential to improve outcomes for patients with pneumonia is, therefore, of high importance.**

**Advance over existing knowledge:**

**Four previous models to predict the similar outcomes are cited and the authors compare their model to these previous models. These previous models use death as the outcome to be predicted and are based on data from hospitalized patients. The BIOTOPE study instead uses data from patients in a primary care setting and has hospitalisation and/or death as the outcome to be predicted. The data from the 2016 cohort of the BIOTOPE study were previously published, but the 2022/23 cohort data have not previously been published. The new data and model, therefore, represent an important advance over these previous studies.**

**Interest to clinicians and policymakers who are not specialists in this topic:**

**The application of machine learning to predict patient outcomes and support clinical decisions is a widely used approach across fields of medical research. The study is, therefore, likely to be of interest to clinicians and policymakers developing similar tools in other fields of medicine.**

**What are the main claims of the paper and how significant are they for the discipline?**

**The main claim of the paper is that the authors have developed a machine learning model to predict hospitalization and/or death from pneumonia that outperforms previous models. Such a claim, if it holds up upon better measures of model evaluation (see comments below), appears to be significant for the discipline. However, I am not an expert in pneumonia so others would be better placed to judge.**

**It is unsurprising that the model outperforms previous models as these are trained using data from a different setting and with a different outcome. The fact that other models based on the same setting as used in this study do not exist is a strength of the current work.**

**1.**	**There is only 1 death event between the training and testing cohorts. Therefore, the focus of the manuscript should be on hospitalisation, e.g. "death" could be removed from the title.**

The manuscript does not attempt to predict death as an outcome on its own; instead, death and hospitalization are considered together as a composite outcome. Removing the single individual who died from this composite outcome would have minimal impact on the results, given that it is a single observation. Given that this outcome was defined prior to the study start, we feel that it is appropriate to retain it in its current form and highlight in the Discussion that the death component of this outcome is represented by a single case.

We have added the following to the discussion:

*Death is a more objective representation but is a rare outcome in primary care cohorts with only one death in our cohort. However, in primary care, one of the main objectives is to appropriately identify those who require hospitalisation. As such, we consider that this is a useful initial outcome measure. Further studies validating this–and alternative–score(s) should be conducted.*

We also note in the discussion that, *“We used a marker of severity defined by hospitalisation and/or death within seven days of assessment. Study participants may have been hospitalised for reasons other than severe pneumonia and hospitalisation is a subjective decision dependent on a number of factors relating to the health system, health worker and family”*.

We accept that hospitalisation is a complex decision influenced by a number of factors which are non clinical as highlighted by the reviewer. However, we do believe it is a legitimate outcome measure particularly in primary care where this study is based – particularly given that one of the main decisions is whether to escalate care or not. From a health systems perspective, hospital admission also directly reflects economic and logistical burden. Hospitalisation frequently represents the threshold at which significant healthcare costs are incurred.

**2.**	**A good explanation of how the work fits in the context of previous literature and policy guidelines is given. However, more emphasis on the differences between the settings and outcomes of the different models, which is well articulated in the Discussion section, should be explained in the Introduction.**

We have added the following sentence to the Introduction:

*It is important to note that risk models explored for comparative purposes involved hospitalised patients and used mortality alone as an outcome measure, implying a sicker cohort than BIOTOPE, which involved primary care patients only.*

**3.**	**Clarification of model evaluation procedures together with additional measures of model performance are necessary to judge whether the data and analyses support the claims.**

**Model probability calibration was not performed. This performance measure is key to understanding uncertainty in the model predictions.**

We thank the reviewer for bringing this to our attention. Model calibration results have now been included in the supplemental file.

The *Prediction of severity of childhood pneumonia using machine learning in BIOTOPE training data* section of Results now states:

*Predicted probability calibration was performed by fitting a logistic model to the relationship between predicted probability and observed probability.*

While we note the importance of a well-calibrated model, we also note that an accurate ordering of patients provides utility even in cases where probabilities are not well-calibrated.

**4.**	**The suitability of the approach for handling missing data requires investigation. Specifically, it is important to consider whether the missing data were the data missing at random or not. Sensitivity analysis should be performed to understand whether the results are robust to the strategy used to handle missing data.**

The data are missing at random (MAR) albeit not missing completely at random (MCAR). In the supplemental file. We also generated model performance results on missingness-free datasets; in particular, for both the BIOTOPE training cohort and BIOTOPE testing cohort datasets, we removed all patients for which any data were missing; we then trained our model on the resulting BIOTOPE training cohort data and tested performance of the derived model on the resulting BIOTOPE testing cohort data. For the missingness-free datasets, we achieved a test dataset AUC of 0.85 (0.87 for the imputed dataset) and a test dataset PRAUC of 0.68 (0.57 for the imputed dataset). Although the AUC is improved for the missingness-free dataset, this may be because some of the more difficult-to-classify patients are removed. For this reason, we report the results on the imputed dataset in the main manuscript.

**5.**	**Table 2: Measures of uncertainty, e.g. standard deviation, should be included in addition to mean for model performance metrics.**

We thank the reviewer for bringing this oversight to our attention. We have now included standard deviations for all model performance results.

**6.**	**Within the discipline of applied machine learning in medicine the study, in its present form, is not outstanding as the model evaluation procedure does not follow best practice (e.g. by following TRIPOD/TRIPOD-AI guidelines).**

We have included the TRIPOD AI checklist in this version; furthermore, we have included the following to the Study Population section Methods:

*This study is reported as per the Transparent Reporting of a Multivariable Prediction Model for Individual Prognosis Or Diagnosis (TRIPOD) AI statement (S1 Checklist).*

**7.**	**The novelty is in the data rather than the machine learning, which makes use of classical methods. Four different classes of machine learning model were trained and evaluated. However, models which have been shown to exhibit excellent performance on tabular data, e.g. XGBoost, were not studied. The application of a tabular foundation model (Hollmann et al. Nature 2025) could be a novel methodological approach to try, but not necessary.**

We thank the reviewer for bringing the above study to our attention. We have added XGBoost to the list of candidate models used for model selection and discussed the corresponding results throughout the main manuscript. In general, the best random forest parameterisation still outperforms the XGBoost parameterisations explored.

We have also added the following to the Model Selection subsection of Methods:

*XGBoost models (*$base learner\times learning rate; base learner \in\{tree, linear model\}, learning rate \in\{0.1, 0.3, 0.5\}$*) were also explored using the xgboost Python package …*

**8.**	**The study does not follow the TRIPOD or TRIPOD-AI guidelines, which are highly relevant for the study. The quality of the modelling would be substantially improved by following these guidelines.**

We have included the TRIPOD AI checklist in this version.

**9.**	**It is not clear how the performance evaluation on the training data was done. From the description, it does not appear that nested cross-validation was used for internal validation of the model. If not performed, this internal validation strategy would lead to "data leakage" and overoptimistic estimates of model generalizability. It is important to clarify this point.**

We thank the reviewer for raising this point. Model performance is reported to justify selection of a specific method and corresponding parameterisation. The selected model/parameterisation is then assessed on the unseen BIOTOPE testing cohort data. However, nested cross-validation was performed using the model selection candidates; the results of this procedure are now included in the main manuscript. In particular, the *Prediction of severity of childhood pneumonia using machine learning in BIOTOPE training data* section of Results now states, “*... (a nested cross-validation procedure yielded an mean AUC of 0.94 (sd: 0.01) and a mean PRAUC of 0.81 (sd: 0.04)).*”

**10.** **Page 6, Study population: It is not clear whether the sites included in the 2016 cohort are a subset of those included in the 2022/23 cohort or different sites. This point should be clarified.**

I will clarify this. 2016 sites are also in 2022/2023 sites. We have added details on this to table S1 in the supplemental file

**11.** **Page 8, Model selection: It is not clear whether the feature selection was performed separately for the training and testing data. If feature selection was performed once on all the data, rather than separately for the training and testing data, there would be "data leakage" resulting in overoptimistic estimates of model generalizability. This point should be clarified.**

Feature importance was only performed on the BIOTOPE training cohort data. The resulting importances were not used in any downstream analysis. We have made this clearer in the Methods section of the main manuscript.

The *Feature importance analysis* section of Methods now reads:

*Feature importance analysis was only performed on the BIOTOPE training cohort data; furthermore, derived importances were not used in any downstream analyses.*

**12.** **The names and version numbers of software packages used should be included in the Methods section so that the analysis can be replicated.**

Software packages and version numbers have been added to the main manuscript.

**13.** **Abstract: "background" is spelled incorrectly.**

We thank the reviewer for bringing this to our attention. This has been corrected.

**14.** **Page 6, Study population: "difficult breathing" be "difficulty breathing"?**

We thank the reviewer for bringing this to our attention. This has been corrected.

**15.** **Page 7, Data preprocessing: It would be informative to state the number of covariates and a brief description of what kind of data they are, e.g. demographic, symptoms, in the Methods section text as these may not be obvious to a general medical audience. It would also be informative to add a correlation matrix for the covariates and outcome data as a supplementary figure to visualise how they are all related to each other.**

We thank the reviewer for their comment and note that we have now provided a brief descriptor for each covariate and included a correlation matrix in the supplemental file.

16. **Page 8, Model selection: "most optimal" should be "optimal"**.

We thank the reviewer for bringing this to our attention. This has been corrected.

**For studies that develop a prediction model or evaluate its performance, please ensure that the study is reported according to the TRIPOD statement (**[**https://www.equator-network.org/reporting-guidelines/tripod-statement**](https://www.equator-network.org/reporting-guidelines/tripod-statement)**) and include the completed checklist as Supporting Information. Please add the following statement, or similar, to the Methods: "." This study is reported as per the Transparent Reporting of a Multivariable Prediction Model for Individual Prognosis Or Diagnosis (TRIPOD) statement (S1 Checklist)For studies using machine learning, please use the TRIPOD-AI checklist. When completing the checklist, please use section and paragraph numbers, rather than page numbers.**

This has been added to the supplemental file

**Journal Requirements:**

**1) Please ensure that the funders and grant numbers match between the Financial Disclosure field and the Funding Information tab in your submission form. Note that the funders must be provided in the same order in both places as well.**

**- State the initials, alongside each funding source, of each author to receive each grant.**

**- State what role the funders took in the study. If the funders had no role in your study, please state: “The funders had no role in study design, data**

**collection and analysis, decision to publish, or preparation of the manuscript.”**

This has been completed

**2) We have amended your Competing Interest statement to comply with journal style. We kindly ask that you double check the statement and let us know if anything is incorrect.**

This has been done

**3) We have noticed that you have uploaded Supporting Information files, but you have not included a list of legends. Please add a full list of legends for your Supporting Information files after the references list.**

This has been done

**4) Figure S1: please (a) provide a direct link to the base layer of the map (i.e., the country or region border shape) and ensure this is also included in the figure legend; and (b) provide a link to the terms of use / license information for the base layer image or shapefile. We cannot publish proprietary or copyrighted maps (e.g. Google Maps, Mapquest) and the terms of use for your map base layer must be compatible with our CC BY 4.0 license.**

This was created in Google Maps but is not a major requirement for the article as they are listed in the table and we have updated the table with comments from reviewers. This makes the map redundant so we have removed the map

**5) Figure S12: Please confirm (a) that you are the photographer; or (b) provide written permission from the photographer to publish the photo(s) under our CC BY 4.0 license**

I can confirm that Joe Gallagher is the photographer and so no permission is required
