# Supplementary material for: Prediction of hospitalisation in young children with pneumonia in Malawi: A machine learning-based approach
Source: PLoS Med. 2026 Jun 9;23(6):e1005122. doi: 10.1371/journal.pmed.1005122 (PMC13271506; doi:10.1371/journal.pmed.1005122)
Supplement: Attachment — Submitted filename: Response to Reviewers (1).docx [file pmed.1005122.s006.docx]

**Response to Reviewers**

-----------------------------------------------------------
Comments from the academic editor:

**In terms of response to my comments, I would like to know a little more about the Integrated Community Health Information System (iCHIS). Is something that is being implemented widely with complete data collection? It wasn't clear what could be expected in terms of everyone having this level of extensive available. There are many detailed data collection efforts though completeness is often a very different story.**

The Integrated Community Health Information System (iCHIS) is a Malawi Ministry of Health–owned national platform being rolled out in a phased manner at present. It is built on the DHIS2 platform. iCHIS operates in selected districts with partial or full deployment, reflecting variability in Health Surveillance Assistant (HSA) training, supervision, device availability, and connectivity. While iCHIS enables detailed household- and individual-level data capture, data completeness varies in practice due to non-mandatory fields although key clinical and demographic fields are mandatory fields. These limitations are well recognised within the national digital health strategy and are typical of large-scale community health information systems in low-resource settings. Although an initial study describing communities of practice has been published (Kanjo et al below), further work to fully describe the implementation of iCHIS is still ongoing and so not possible to report in detail at present.

Importantly, the BIOTOPE AI algorithm is well aligned with both the current functionality and future trajectory of iCHIS in terms of design. This is the national and only community IT record in use in Malawi at present. The score can be integrated as a lightweight analytic layer within existing iCHIS workflows without requiring universal data completeness or additional data collection burden. This approach aligns with Malawi’s national digital health architecture, which prioritises interoperable, modular systems built on DHIS2. Currently we are undertaking an implementation science based study on the integration of the BIOTOPE AI tool into the iCHIS system which will allow us to answer this question more completely although initial feedback from health workers is promising and we will report these findings in due course. In order to describe the iCHIS system more fully we have added a section to the supplementary file in the appendix on it.

## **Supplemental File: S14 Overview of the Integrated Community Health Information System (iCHIS) in Malawi**

The Integrated Community Health Information System (iCHIS) is a Ministry of Health–owned digital platform designed to support routine data collection, clinical decision support, supervision, and reporting at the community level in Malawi. Developed on the DHIS2 Tracker architecture, iCHIS replaces fragmented, paper-based and program-specific tools with a unified system that integrates household registration, individual longitudinal records, disease surveillance, service delivery, supervision, and reporting within a single interoperable platform. The system is primarily used by Health Surveillance Assistants (HSAs) via Android tablets in offline mode, with synchronization occurring when connectivity is available, and by supervisors and managers through a web-based interface for monitoring and decision-making

Deployment of iCHIS is following a phased national rollout strategy led by the Ministry of Health in collaboration with multiple implementing partners. Rollout includes district readiness assessments, digital literacy training, training of trainers, supervised field practice, and ongoing technical support. As of2025, iCHIS has been deployed with partial or full coverage in approximately 14 districts, reaching about 19% of the national community health workforce and serving over four million individuals. Of the planned system modules, eleven have been developed, including foundational registers (community, household, and person), integrated community case management, maternal and neonatal care, immunization, non-communicable diseases, reporting, supervision, and electronic disease surveillance. Deployment intensity varies by district depending on partner support, infrastructure readiness, and availability of devices and supervision capacity

In routine use, iCHIS supports point-of-care service delivery, real-time clinical guidance, and longitudinal tracking of individuals and households. Overall, iCHIS represents a maturing national digital health platform that has substantially improved integration and availability of community-level data, while continuing to face systemic constraints commonly seen in large-scale digital health implementations. It continues to be the single IT enabled community health information system in Malawi

Kanjo C, Manda TD, Byson L, Twabi A, Munthali KG. Community of Practice in Practice: Successful Implementation of Integrated Community Health Information Systems. In: 2022 IST-Africa Conference (IST-Africa) [Internet]. Ireland: IEEE; 2022 [cited 2025 Oct 12]. p. 1–8. Available from: https://ieeexplore.ieee.org/document/9845579

-----------------------------------------------------------
Comments from the reviewers:

**Reviewer #1: I appreciate that the authors have addressed the previous comments. However, since the dataset includes only one death case, it is not appropriate to claim that the model can predict mortality. I would also recommend revising the manuscript title to "A Machine Learning Algorithm to Predict Hospitalisation in Childhood Pneumonia in Malawi" to better reflect the scope and location of the study.**

We thank the reviewer for this comment and have changed the title to reflect this


**Reviewer #2:**

**In science, we have an obligation to present our work and findings accurately, ensuring that our readers understand the results and can determine whether they are generalizable to different settings. By clinging to a composite hospitalization/death outcome, despite only 1 death in the entire dataset, the authors have chosen, in my humble opinion, to misrepresent their work. It doesn't matter whether a paragraph in the discussion touches on this point or if this was a pre-specified endpoint. Simply including death as a potential outcome that this tool could predict in the title is misleading at best. This is the primary reason why I urge rejection of this manuscript.**

We thank the reviewer for their detailed comments and for underscoring the importance of accurate outcome definition and transparent reporting. We respectfully disagree that inclusion of the prespecified composite hospitalisation/death outcome misrepresents the study or warrants rejection of the manuscript. However, in line with the comments of Reviewer 1 we have changed the title to “A Machine Learning Algorithm to Predict Hospitalisation in Childhood Pneumonia in Malawi" and detail in the manuscript to better reflect the scope and location of the study.

As clearly stated in the Discussion, the outcome definition reflects both methodological pragmatism and clinical relevance in a primary care setting. We explicitly acknowledge the rarity of mortality in outpatient cohorts and the limitations this imposes:

“Death is a more objective representation but is a rare outcome in primary care cohorts with only one death in our cohort. However, in primary care, one of the main objectives is to appropriately identify those who require hospitalisation. As such, we consider that this is a useful initial outcome measure.” We have added the following text and references to highlight this also. This is consistent with other studies showing low mortality in primary care

32.	Falade AG, Uchendu OC, Bakare AA, Colbourn T, Olojede OE, Abuo JK, et al. Outcome and management of 2-59-month-old Nigerian children with chest indrawing pneumonia at primary-level healthcare facilities: a prospective cohort study. J Glob Health. 2026 Jan 12;16:04004.

33.	Jacobs C, Nkwemu C, Ngambi BB, Silavwe V, Qazi SA, Nisar YB. Outcomes of children aged 2-59 months with chest indrawing pneumonia managed on an outpatient basis in selected primary health facilities in Zambia. J Glob Health. 2025 Jul 11;15:04089.

34.	Tigabu Z, Toni AT, Guadu T, Yilma TM, Awoke T, Engdaw GT, et al. Management and outcomes of chest-indrawing pneumonia among children aged 2-59 months in a programme setting in Ethiopia: a prospective observational study. J Glob Health. 2025 Aug 4;15:04217.

35.	Suhag ZH, Pal A, Naeem M, Ahmed I, Khuwaja NA, Khakwani S, et al. Outcome and management of children with chest indrawing pneumonia at primary health care settings in Pakistan: an observational cohort study. J Glob Health. 2025 Mar 28;15:04096.

The composite outcome was pre-specified and chosen to reflect the real-world decision-making challenge faced by frontline health workers, namely identifying children at risk of deterioration who require referral. As further discussed:

“In primary care, the central question for health workers is not only whether a child will survive, but whether they are at risk of deterioration and therefore require referral for higher-level care.” and we have now highlighted in the text the issue of treatment failure and provided the following reference

.	Pathak BG, Nisar YB, Natchu UCM, Manapurath R, Thakur A, Mazumder S, et al. Management of chest indrawing pneumonia in children aged 2-59 months by community-level workers compared to standard care on clinical outcomes: systematic review and meta-analysis. J Glob Health. 2025 Jun 20;15:04169.

We also explicitly recognise that hospitalisation is a subjective outcome influenced by health system and clinician factors:

“Study participants may have been hospitalised for reasons other than severe pneumonia and hospitalisation is a subjective decision dependent on a number of factors relating to the health system, health worker and family.”

These limitations are openly acknowledged and are presented as areas for further validation rather than overstated conclusions. In response to reviewer feedback, and consistent with this framing, we have revised the title and manuscript to clearly emphasise that the model’s primary utility lies in predicting hospitalisation rather than mortality.

**Additional points relavent to why I recommend rejection of this manuscript:
Utilization of the Ministry of Health hospitalization criteria to predict hospitalization is not methodologically sound. Just integrate a checklist for indications for hospital referral into iCHS. Just because a clinician makes a decision to hospitalize a patient or not hospitalize them it does not mean that decision was sound - this is why post-discharge follow up data is crucial and death is an important outcome that needs to be taken into consideration**.

We also thank the reviewer for raising concerns regarding the methodological validity of using hospitalisation as an outcome and for highlighting the potential fallibility of clinician decision-making. We respectfully disagree with the assertion that this approach is unsound.

This was a multi-site study conducted across geographically and temporally distinct primary care settings, involving health workers who underwent rigorous, standardised training in Integrated Management of Childhood Illness (IMCI) prior to study initiation. Hospitalisation decisions were therefore made by trained clinicians applying nationally and internationally endorsed guidelines, reflecting real-world practice in the settings for which the tool is intended.

The comparator used—the WHO danger signs—is itself a checklist already embedded within IMCI and iCCM.

We have highlighted these issues in the discussion “There have been concerns regarding the ability of risk scores to predict poor outcomes in child pneumonia(5,10,27) An external validation study noted that although pneumonia risk scores have performed well among the cohorts in which they were derived, their performance diminished when externally applied(28). A study from Malawi showed only 37.2% of children hospitalised with pneumonia had a WHO danger sign(27). Although concerns may arise that this is due to admission of low risk children, a further study showed 39% of fatal cases of pneumonia in children in hospital in Kenya were defined as having non-severe pneumonia(5).”

We agree that hospitalisation decisions may not always be optimal and that post-discharge outcomes, including mortality, are critically important. This study did follow up patients for 7 days following initial assessment. Death is a rare outcome and in primary care identification of a child needs referral to a higher level of care is an important predictive outcome

Importantly, we also demonstrate that the model’s predictions are clinically plausible:

“The identified variables, such as chest indrawing, heart rate, temperature and markers of nutrition, have been associated with poorer outcomes in other studies. This provides reassurance that the model is clinically sensible.”

As outlined in the discussion also

“Death is a more objective representation but is a rare outcome in primary care cohorts with only one death in our cohort. However, in primary care, one of the main objectives is to appropriately identify those who require hospitalisation. As such, we consider that this is a useful initial outcome measure.”

We believe that comparing this tool against established, internationally endorsed referral criteria, applied by rigorously trained health workers across multiple sites, is both methodologically appropriate and clinically relevant.

**Reviewer #3: We thank the authors for addressing our previous comments, and note the planned future validation of the model.**

Thank you


**Reviewer #4: I wish to thank the authors for addressing my comments. In my opinion, the manuscript is now improved. I have a few remaining points, which I think are important to address:**

**I thank the authors for adding the model probability calibration. However, it is not clear to me whether this has been performed on the training data (in which case it should also be performed on the testing data - probability calibration should be included in both the nested cross-validation procedure for internal validation and external validation, just like AUC and AUPRC) or the testing data (in which case the description should be moved to the subsection describing the external validation, and also included as part of the nested cross-validation procedure). Moreover, the authors have only added one sentence to the text to state that probability calibration was performed. They should add a description and a discussion of the calibration result. From the figure it can be seen that there is substantial miscalibration. For the calibration plot, the unity line (y = x) should be added so that the miscalibration can be clearly appreciated. Also, the slope and y-intercept of the
logistic regression model should be added to the plot and described in the main text. The probability calibration is arguably the most important measure of the predictive performance of a model. In reality, users of the model for are given a predicted probability of hospitalisation or death and it is important to understand the accuracy of that predicted probability. The model discrimination (as assessed by the AUC) indicates how well the model correctly predicts the outcomes of two individuals that have different outcomes, which is not the scenario that users of the model face. Therefore, it is vital to describe and discuss the probability calibration in more detail. Also, the comparison with the other models should include assessment of the probability calibration.**

Thank you for this detailed and constructive comment. We agree that probability calibration is a critical component of predictive model performance and that it requires clearer description in the paper.

To address the concerns, we have made the following changes to the manuscript:

First, we have clarified that probability calibration is incorporated into the model training and evaluation process, alongside discrimination metrics. As now stated in the final sentences of the Model Selection subsection of the Methods:

"In the case of model selection, probability calibration was performed after initial model training by fitting a logistic model to the relationship between predicted probability and observed response on previously unused data from the BIOTOPE training dataset. Nested cross-validation (with j=4 outer folds) was performed to evaluate the model selection procedure. The selected model was externally validated on the BIOTOPE testing dataset. The selected model underwent retraining and recalibration on the BIOTOPE training dataset before application to the BIOTOPE testing dataset."

Second, we have expanded the reporting of calibration results in the Prediction of severity of childhood pneumonia using machine learning in BIOTOPE training data subsection. In addition to AUC and PRAUC, we now report the expected calibration error (ECE) for the selected random forest model, both during model selection and within the nested cross-validation procedure. These additions explicitly quantify calibration performance and its variability across folds.

This section now reads:

"We trained several machine learning classifiers on the BIOTOPE training data using stratified k-fold cross-validation and collected information on several performance metrics (Methods). Across all considered classifiers, a random forest classifier trained on all BIOTOPE features in the training dataset exhibited the best performance for both AUC and PRAUC, achieving a mean AUC of 0.95 (Standard Deviation (SD): 0.03) (S3: BIOTOPE model selection) and a mean PRAUC of 0.84 (SD: 0.06); furthermore, this classifier yielded an expected calibration error of 0.08 (SD: 0.05). With a view to quantifying the generalisation error of the cross-validated model selection approach, we employed a nested cross-validation procedure; this procedure yielded a mean AUC of 0.94 (SD: 0.03), a mean PRAUC of 0.84 (SD: 0.04) and an expected calibration error of 0.09 (SD: 0.06))."

Third, we have extended calibration assessment to the external validation setting. In the Dataset shift across BIOTOPE datasets subsection, we now report the ECE obtained when the trained model is evaluated on the BIOTOPE testing dataset, alongside AUC and PRAUC. This addresses the concern that calibration should be evaluated consistently during both internal and external validation.

This text now reads:

"To externally test the derived random forest classifier trained on the BIOTOPE training dataset and assess dataset shift, we tested its performance on the BIOTOPE testing dataset (Methods). The trained classifier achieved a mean AUC of 0.87 and a mean PRAUC of 0.57 on the BIOTOPE testing dataset; this performance was superior to any of the compared methods (Table 2). In addition, the model yielded an expected calibration error of 0.16 (calibration intercept: -9.76, calibration slope: 3.75)."

Together, these changes clarify where and how probability calibration is performed, incorporate calibration into both internal and external validation, and explicitly report calibration performance alongside traditional discrimination metrics, as recommended.

**The authors have responded to my comment about missing data in my previous review. However, I do not think that my point has been adequately addressed in the revised manuscript. From their response to my comment, it seems as though the authors have performed some kind of sensitivity analysis and analysis of whether data are missing at random. However, I cannot see these analyses in the supplement and I cannot see a reference to it in the main text. I strongly suggest including a sensitivity analysis of the method for handling missing data in the supplement and briefly commenting on the results of this analysis, e.g. whether data are missing at random or not and the impact of the strategy for handling missing data on the results, in the main text**.

In order to address this we have added the below subsection to the results section of the main text:

**Sensitivity of model performance to data imputation procedure**

Data imputation facilitates the use of a larger amount of patient data; however, with a view to assessing the effect of the data imputation procedure on model performance, we applied the selected model to datasets filtered to retain only complete observations. When trained and tested on the filtered BIOTOPE training dataset (1,756 observations) (Methods), the model achieved an AUC of 0.95 (SD: 0.02) and a PRAUC of 0.84 (SD: 0.08); when applied to the imputed BIOTOPE training dataset (2,053 observations), the model achieved an AUC of 0.95 (SD: 0.03) and a PRAUC of 0.84 (SD: 0.06). In comparison, when trained on the filtered BIOTOPE training dataset and tested on the filtered BIOTOPE testing dataset (104 observations), the model achieved an AUC of 0.86 and a PRAUC of 0.71; when trained on the imputed BIOTOPE training dataset and tested on the imputed BIOTOPE testing dataset (460 observations), the model achieved an AUC of 0.87 and a PRAUC of 0.57.

**I wish to thank the authors for adding the correlation matrix. I recommend changing the colorscale so that it is diverging and centered at 0, e.g. red (+1), white (0), blue (-1).**

This has been updated

**In the abstract, the quoted model AUC and AUPRC values are described as "mean" values. The external validation values should be single values rather than means. I suspect that this phrasing is a typo.**

This is correct. Thank you for identifying this and we have corrected this.

**In the abstract, the wording used implies that "more dynamic prediction" is facilitated by machine learning. However, this is not the case. A traditional statistical model could just as easily be updated over time. Please rephrase this misleading wording**.

We have rephrased this to “The use of machine learning offers the promise of significantly improved prediction of pneumonia severity”

**In the introduction on page 5, the authors describe using machine learning and artificial intelligence techniques. However, these are not two distinct approaches employed in the current study. Therefore, I suggest just using "machine learning" (or clarify what the difference is in the context of this study).**
We have kept the reference to machine learning techniques alone throughout
