# Supplementary material for: Prediction of hospitalisation in young children with pneumonia in Malawi: A machine learning-based approach
Source: PLoS Med. 2026 Jun 9;23(6):e1005122. doi: 10.1371/journal.pmed.1005122 (PMC13271506; doi:10.1371/journal.pmed.1005122)
Supplement: Attachment — Submitted filename: Reviewer response3.docx [file pmed.1005122.s007.docx]

We thank the editor and reviewer for the further comments and opportunity to further develop the paper based on these. Our responses are below.

**Comments from the academic editor:**


**In the Methods, they could specify there that there was only 1 death in their dataset rather than just say low-mortality.**
This has been updated to read “*Due to only one death in this cohort this model is best used to assess hospitalisation risk*”

**I think abstract should also make this type of clarification somewhere as well. Could be a limitation at end of abstract.**

This has been updated to include the line: “*However, mortality-related findings are not applicable as there was only one death in this cohort*.”

**In some ways, a good description of their outcome is children at risk of poor outcomes but okay to stay with hospitalization too (just another way of describing the meaning of the outcome).
With regards to R2 other comment, death and/or objective deterioration would certainly be better outcomes to use if feasible. But in this circumstance where it isn't feasible, I do still think hospitalization is a reasonable outcome. If there is a more robust objective "indications for hospitalizations" that go beyond WHO Danger Signs that could be specified as an alternative outcome, it would strengthen the paper to demonstrate the models performance against that as well.**

We agree that other objective indications for hospitalisation would be helpful but the WHO Danger Signs are the internationally accepted criteria for hospital referral in children, embedded within IMCI and iCCM frameworks and endorsed by the WHO and the Ministry of Health in Malawi and many other countries. They represent the current standard against which referral decisions are benchmarked globally, and their use as a comparator is, we believe, therefore methodologically appropriate.

However, we are able to demonstrate that the model's strong predictive performance is not confined to children who meet WHO Danger Sign criteria. eFigures 7 and 8 specifically demonstrate that the model performs robustly in predicting hospitalisation among children without WHO Danger Signs — a group in whom conventional severity criteria would not trigger referral. This is arguably the most clinically important subgroup, as these are the children most at risk of being missed by current guidelines. The model's ability to identify at-risk children in this group provides evidence of its added value beyond current internationally endorsed criteria, and further supports the validity and utility of hospitalisation as the outcome measure. We have added to the following paragraph in the paper

*“In order to determine if children hospitalised without a current criterion for admission based on the WHO danger signs could also be identified by the BIOTOPE algorithm, we evaluated the performance of the model in those without WHO danger signs (eFigure 7 and 8). In this case, the BIOTOPE random forest model (excluding children with a WHO danger sign) had an AUC of 0.95 (SD: 0.02) and a PRAUC of 0.80 (SD: 0.07) (probability calibration logistic regression intercept and slope (0.26,1.07) respectively). This is a clinically important subgroup, as these are the children most at risk of being missed by current guidelines. The model's ability to identify at-risk children in this group provides evidence of the added value of this model.”*

Also, in primary care the clinical imperative is to identify those children who require a higher level of care, rather than to predict mortality alone. Mortality is an important outcome but is a rare event in primary care cohorts — a finding consistent with the literature cited in our paper and reflected in the single death recorded in our training cohort. Hospitalisation therefore captures the clinically actionable decision point at which this tool is intended to support health workers, making it not only a pragmatic but an appropriate primary outcome.

-----------------------------------------------------------
**Comments from the reviewers:**

**1) Most of the references to probability calibration focus on expected calibration error. The calibration intercept and slope are more informative measures of probability calibration than expected calibration error, which does not provide information on whether the predicted probabilities are under- or overestimates, and depends on binning. Therefore, calibration intercept and slope should be reported in addition to the expected calibration error and these values should be reported in the main text wherever AUC and PRAUC are described instead of, or in addition to, the expected calibration error.**

We thank the reviewer for this suggestion. This has been implemented.

**2) The Discussion should include a detailed interpretation of the probability calibration results and these findings should be referenced in the Conclusion and Abstract. The model appears to have very poor probability calibration when tested on the external dataset (intercept = -9.76, slope = 3.75). It is vital that readers are aware of this substantial limitation when trying to decide if and how they should use the model. This discussion could also mention recalibrating the model for different settings.**

These results are now referenced in Conclusion and Abstract. It appears that there has been some confusion resulting from the “calibrated intercept” and “calibrated slope” that we provided. The intercept and slope (intercept = -9.76, slope = 3.75) we provided pertain to the parameters used to transform the raw probabilities into the calibrated probabilities on the training data, not the relationship between the calibrated probabilities and the severity status of the patients on test data. Note that even if no calibration had been required in training, we would still not expect an intercept of 0 and a slope of 1, as we used a logistic regression to fit the log-odds of a case as a function of the uncalibrated probability. The documents have now been updated to include an evaluation of the calibrated probabilities on the test data via a logistic regression model. In this case the parameters correspond to the logistic regression model fitted using the severity status as the response variable and log-odds of the calibrated probability as the predictor variable. As a consequence, a perfectly calibrated model is expected to produce an intercept value of 0 and a slope value of 1. We obtained -0.32 and 1.13 for the intercept and slope, respectively, on the test data.

**3) eFigure 3, showing the probability calibration results, does not appear to be referenced anywhere in the main text. A reference to this figure should be added to clarify which part of the text relates to it. It is not clear to me how the logistic regression model described in the Methods and probability calibration slope and intercept quoted in the main text relate to eFigure 3. Also, this figure appears to show different results from the previous version of the manuscript (although I could be wrong about this point).  These points should be clarified. The figure caption should describe what the figure in more detail, e.g. what does the orange line represent?**

We thank the reviewer for these observations. eFigure3 is now referenced in the main manuscript. We have now clarified the approaches used to evaluate the probability calibration in the methods; we have also expanded on the description of the figure caption for eFigure3. The results given represent the most up-to-date results and analyses.

**4) Table 2, eTable 6 and eTable 7 and Figure 2, eFigure 2, eFigure 4, eFigure 7 and eFigure 8 do not include the probability calibration performance results. Probability calibration results are complementary to AUC and PRAUC and so should be added to assess model performance anywhere AUC and PRAUC have been used.**

Tables and figures have been updated such that information on calibrated intercept and slope estimates is available for all devised models.

**5) In the new text under "Model selection" in the Methods section, "logistic model" should be "logistic regression model”**

We thank the reviewer for this. This has now been corrected.
