# Supplementary material for: Prediction of hospitalisation in young children with pneumonia in Malawi: A machine learning-based approach
Source: PLoS Med. 2026 Jun 9;23(6):e1005122. doi: 10.1371/journal.pmed.1005122 (PMC13271506; doi:10.1371/journal.pmed.1005122)
Supplement: Attachment — Submitted filename: Response to editor.docx [file pmed.1005122.s008.docx]

**Response to editor:**

***We would suggest the following edits to the Abstract: 'Key limitations include the use of hospitalisation and/or death as a severity outcome, which may reflect health system factors rather than true disease severity, mortality-based comparisons were not possible due to low mortality in these primary care cohorts, and comparator tools having been develop for hospital populations rather than primary care populations.**

This has been done

***Please also revise the conclusions section of the Abstract to summarise what can be concluded from the study, since at the moment if focuses too much on what cannot be concluded.**
This now reads

This machine learning score outperformed traditional pneumonia risk scores in predicting hospitalisation within 7 days in Malawian children presenting to primary care. Traditional pneumonia risk scores diminish in performance when externally applied to new datasets suggesting they may not generalise well beyond their original derivation settings. Mortality-related findings are not applicable as there was only one death in this cohort. Overall these findings support the potential of machine learning to meaningfully improve early identification of children at risk of severe pneumonia in low-resource primary care settings. Further external validation and clinical impact studies are needed to confirm these results.

***Please move the Author Summary to immediately after the abstract.**

This has been done

***Please briefly mention in the Author Summary what happened in the training dataset.**
We have added: Multiple classifier types were evaluated using cross-validation, with a random forest model trained on clinical features—including temperature, weight-for-age, chest indrawing, heart rate, and respiratory rate—selected as the best performer.

***We would suggest the following edits to the Author Summary: 'This study shows that a machine learning–based approach may more accurately identify children at risk of poor outcomes from pneumonia in primary care settings than current risk scores.**

This has been done

*** Please check that any use of the word significant is supported by the data, and if not please remove it.**

We have not identified the use of the word “significant” or “significantly” in reference to data in the paper
 *** In the Competing interests statement please replace 'The remaining authors have no conflicts of interest to declare.' with 'The other authors declare that no competing interests exist.'**
This has been done

*** Please ensure that your code is shared in a way that follows best practice and facilitates reproducibility and reuse. Because Github depositions can be readily changed or deleted, we request that you to make a permanent DOI'd copy (e.g. in Zenodo) and provide the URL in the Data Availability statement.**

We have added a Zenodo DOI 10.5281/zenodo.20234098

***In the ethics statement, please clarify if the word 'guardians' refers to legal guardians and update the text accordingly.**

We have added that “Under the Malawi Child Care, Protection and Justice Act, a guardian is an adult legally responsible for a child's welfare, upbringing, and property.”
