# Supplementary material for: Prediction of hospitalisation in young children with pneumonia in Malawi: A machine learning-based approach
Source: PLoS Med. 2026 Jun 9;23(6):e1005122. doi: 10.1371/journal.pmed.1005122 (PMC13271506; doi:10.1371/journal.pmed.1005122)
Supplement: Peer review history

# Editor report: Decision Letter 0

**DOI:** 10.1371/journal.pmed.1005122.r001

**Authors:** Andreia Cunha

7 May 2025

Dear Dr Gallagher,

Thank you for submitting your manuscript entitled "A Machine Learning Algorithm to Predict Hospitalisation and/or Death in Childhood Pneumonia" for consideration by PLOS Medicine.

Your manuscript has now been evaluated by the PLOS Medicine editorial staff and I am writing to let you know that we would like to send your submission out for external peer review.

However, before we can send your manuscript to reviewers, we need you to complete your submission by providing the metadata that is required for full assessment. To this end, please login to Editorial Manager where you will find the paper in the 'Submissions Needing Revisions' folder on your homepage. Please click 'Revise Submission' from the Action Links and complete all additional questions in the submission questionnaire.

Please re-submit your manuscript within two working days, i.e. by May 09 2025 11:59PM.

Login to Editorial Manager here: https://www.editorialmanager.com/pmedicine

Once your full submission is complete, your paper will undergo a series of checks in preparation for peer review. Once your manuscript has passed all checks it will be sent out for review.

Feel free to email me at acunha@plos.org if you have any queries relating to your submission.

Kind regards,

Andreia Cunha, PhD

Senior Editor

PLOS Medicine

# Peer review: Decision Letter 1

**DOI:** 10.1371/journal.pmed.1005122.r002

**Authors:** Andreia Cunha

15 Jul 2025

Dear Dr Gallagher,

Many thanks for submitting your manuscript "A Machine Learning Algorithm to Predict Hospitalisation and/or Death in Childhood Pneumonia" (PMEDICINE-D-25-01572R1) to PLOS Medicine and sincere apologies for the delay in getting back to you with a decision. The paper has been reviewed by subject experts and a statistician; their comments are included below and can also be accessed here: [LINK]

As you will see, the reviewers find the work of potential interest but raise a number of substantive concerns about the claims made in light of the evaluation of performance presented and about the potential clinical utility. After discussing the paper with the editorial team and an academic editor with relevant expertise, I'm pleased to invite you to revise the paper in response to the reviewers' comments resolving the concerns in full. We plan to send the revised paper to some or all of the original reviewers, and we cannot provide any guarantees at this stage regarding publication.

In addition to these revisions, you may need to complete some formatting changes, which you will receive in a follow up email. A member of our team will be in touch with a set of requests shortly. If you do not receive a separate email within a few days, please assume that checks have been completed, and no additional changes are required.

When you upload your revision, please include a point-by-point response that addresses all of the reviewer and editorial points, indicating the changes made in the manuscript and either an excerpt of the revised text or the location (eg: page and line number) where each change can be found. Please also be sure to check the general editorial comments at the end of this letter and include these in your point-by-point response. When you resubmit your paper, please include a clean version of the paper as the main article file and a version with changes tracked as a marked-up manuscript. It may also be helpful to check the guidelines for revised papers at http://journals.plos.org/plosmedicine/s/revising-your-manuscript for any that apply to your paper.

We ask that you submit your revision by Oct 07 2025 11:59PM. However, if this deadline is not feasible, please contact me by email, and we can discuss a suitable alternative.

Don't hesitate to contact me directly with any questions (acunha@plos.org).

Best regards,

Andreia

Andreia Cunha, PhD

Senior editor

PLOS Medicine

acunha@plos.org

-----------------------------------------------------------

Comments from the academic editor:

- I don't think the authors address how and why this will facilitate a better prediction tool that is implementable, either through better feature selection or weighting. I am not sure the author's were trying to answer the questions from that perspective, but I it is imperative. A prediction tool that is not useable in the intended context is not that helpful even if you can demonstrate better performance. Developing a model that can also be used in routine practice or one that demonstrates some new key insight I think is needed for broader impact.

- I worry that some of their findings are driven by prediction only within their dataset (any scoring tool developed elsewhere likely to perform worse) and the number of variables they have in their dataset. The most important variables still seem to be the ones that would be expected and are likely in the other risk tools.

- I was not totally clear, but it does seem that the authors developed models that only used the features used in other commonly used prediction tools (that are presumably applied in actual practice). I think this is importatn if that is the case, and in many ways those comparisons might be the most important (i.e., demonstrating that applying machine learning to the same variables that are commonly available still improves performance). However, the reviewers also raise important points as to whether those comparisons are even appropriate given the difference of setting (outpatient vs already hospitalized).

-----------------------------------------------------------

Comments from the reviewers:

Reviewer #1: The paper applied machine learning (ML) models to predict the hospitalization and/or death within 7 days after initial presentation in 2-59-month-old Malawian children with pneumonia. The features of the ML models included demographic, assessment finding, vital sign, HIV, lifestyle data. The data were further analyzed using impurity-based and permutation-based feature importance analysis, learning curve analysis, mutual information analysis, and correlation analysis. The suggestions for revising the paper are listed below.

1. In the "Study population" section of methods, the authors have mentioned that the 2016 BIOTOPE cohort was used as testing dataset, while the 2022-2023 cohort used as the training dataset. It would be helpful if the authors to clarify the reason for this choice, as it is more common to use older datasets for model training, and more recent datasets for testing, in order to simulate a prospective validation. Could the author clarify?

2. In this study, one of the predicted outcomes—death—is represented by only a single case within the entire cohort, which is included in the training set, with no corresponding cases in the testing set. Given that machine learning approaches are inherently data-driven and rely on sufficient representation to learn meaningful patterns, a single instance is insufficient to develop a robust or generalizable predictive model. This extreme class imbalance renders the outcome unreliable for model evaluation. I recommend that the authors either redefine this outcome or consider excluding it from the analysis due to the lack of representative data.

3. Age-related variability is an important consideration in pediatric populations, as normal ranges for vital signs vary with development stage. For example, the respiratory and heart rate are typically higher in infants and gradually increases and stabilize with age. Could the authors clarify whether age-based normalization was applied to features such as respiratory rate and heart rate data before inputting them into the machine learning models?

4. In the "Feature importance analysis" section of methods, the authors have described those two distinct approaches for evaluating feature importance were applied: (1) impurity-based approach and (2) a permutation-based. It is hard to understand (what calculations or implementation details were applied?). Could the authors clarify how these methods were applied in this study? Including relevant equations or methodological steps would be helpful for reader understanding.

5. In Table S2, the authors provide an overview of features used in training model. It may be helpful to include the features used in existing models such as RISC, PERCH, and PREPARE for comparison? Additionally, proving the missing rates for BIOTOPE cohort in this table, would help clarify whether model performance may have been influenced by missing data.

6. The three figures, Figure 1 to 3, need to have better resolution. In Figure 3, the text is too small to read; please enlarge the font size or adjust the layout to improve readability.

7. Some of the related ML studies in pediatric pneumonia prediction may be included in the manuscript:

* https://xmed.jmir.org/2025/1/e57719/

* https://medinform.jmir.org/2022/1/e28934

* https://onlinelibrary.wiley.com/doi/abs/10.1002/ppul.26897

Reviewer #2: I commend the author on their ongoing work to reduce pneumonia related mortality in children living in low- and middle-income countries.

My recommendation would be to include RISC-Malawi (doi: 10.1371/journal.pone.0168126 as part of the analysis). This is important as (1) its the only published tool studying the same population and (2) when externally validated by the PREPARE group it outperformed all other clinical prediction tools including RISC and PERCH (doi: 10.7189/jogh.11.04062) making it the best performing pneumonia-related mortality clinical prediction tool that has been externally validated (AUC = 0.75, 95% CI = 0.74-0.77).

Though I am not an expert in machine learning and hope the manuscript will undergo evaluation by a technical expert in data science to ensure the methods given the counts of outcome are appropriate, I commend the authors on their ingenuity. The ML algorithm was designed to identify children with WHO defined pneumonia, diagnosed in outpatient settings in rural Malawi, at risk for hospitalization or death. I am concerned about the adequacy of sample size to determine this.

Inadequate number of deaths in the training (1 out of 1872 cases) and testing (0 out of 212 cases); why is death information missing for 181 and 244 cases in the different cohorts?

Hospitalization/death occurred in 14.3% of training cohort and 12.1% of testing cohort; yet WHO danger signs (an indication for hospitalization) was more prevalent in both cohorts at 17.6% and 15.9% respectively. So what is being described, in terms of hospitalization practices, may be (1) a deviation from national guidance or (2) change in clinical status or (3) reflect differences in interpretation of clinical status at time of presentation to the hospital.

Since WHO danger signs were evaluated as part of the model but they also define the indication for the outcome of interest, I'm unclear if this is a methodologically sound approach.

The prediction tools compared (RISC, PERCH, and PREPARE) were designed to be used in hospitalized/emergency center patients (who were then hospitalized), not children evaluated in outpatient settings. They were designed to predict death - whereas the BIOTOPE tool did not include enough subjects who died to be able to distinguish those who died vs survived their illness. Though I understand the desire to compare these tools, its very important that these distinctions are clear and adequately discussed.

I wish there was more discussion about the actual applicability of the developed tool in outpatient settings. The paragraph on the development of continuous tools didn't sound applicable as inherently children evaluated in outpatient settings are not continuously monitored.

Overall I commend the author's ingenuity but recommend a closer look at if the tool they developed is methodologically sound, should be compared to hospital mortality tools, and, if they choose to compare their tool to hospital mortality tools they should be comparing it to RISC-Malawi as it was derived from a Malawian population hospitalized for pneumonia.

Reviewer #3: This manuscript describes the development and validation of a machine learning-based classifier to predict hospitalization/mortality within 7 days after initial presentation, for Malawian children with pneumonia aged between 2 and 59 months (median about 16 months). A training cohort (BIOTOPE) of 2053 children was collected between 2022 and 2023, and the test cohort of 456 children in 2016. A random forest classifier trained on all BIOTOPE features was found to achieve an AUC of 0.87 on the test dataset, significantly better than existing risk scores (PERCH, PREPARE, RISC, WHO severity criteria)

While the findings are encouraging, some issues might be considered:

1. In the Abstract, "Bakcground" might be "Background".

2. In the Data preprocessing subsection, it is stated that a patient is excluded if more than 50% of their data is missing, and that missing values were imputed. If possible, the percentage of missing data for each feature might be stated in supplementary material.

3. In the Model Selection subsection, it is stated that four classifier families (SVM, naive Bayes, neural network, random forest) were explored, moreover with four different feature subsets. The full set of results for all these combinations should be presented in the supplementary material. Currently, it appears that only the best result for each model is shown (eTable 1). Information as to how each feature subset performs is of practical interest, given that some of the BIOTOPE features might not be available/collected in other studies or scenarios.

4. In the Model Selection subsection, it is stated that for the random forest model in particular, the number of trees was fixed at 1000, and that each tree was grown until maximal purity with (again) fixed parameters based on n_features and n_observations. However, random forests are known to have a number of other possible hyperparameters, such as the number of features considered when splitting a node, the maximum depth of each tree, the maximum number of leaf nodes in each tree, etc. As such, it should be explained in greater detail as to what hyperparameterisation was actually performed for random forests (eTable 1 suggests none), and if not, why those common hyperparameters were not explored (especially as these hyperparameters appear to be available in boruta_py.py in the GitHub repo).

5. In the Results section, it is stated that the model was evaluated on a subset of patients without WHO danger signs. The significance of this exclusion might be explained further - what is special about the subset of patients without WHO danger signs? How are WHO danger signs defined?

6. In the Discussion section, it is stated that the WHO, RISC, PERCH and PREPARE models used only mortality (and not hospitalization) as an outcome measure. Given this the claim that the developed model is superior to those existing models may not be particularly valid, given the extremely low prevalence of death in the BIOTOPE cohort (1/2084). Notably, the exposition suggests only the difference in the input features used as affecting performance, while neglecting the difference in outcome definition. It is later acknowledged that death is a more objective but rare outcome, which may have affected performance for the other risk models.

7. In the Discussion section, while dataset shift (and possible mitigation by continual learning) is discussed, the actual impact of the machine learning model is not explained - will treatment of the patient(s) differ depending on the diagnosis of the model? Moreover, if so, would it be expected that this would in turn also affect the performance and evaluation of the model?

For example, the model might be very successful in flagging patients at risk of impending hospitalization or death. However, because of this predictive power, the patients receive appropriate care, and many of them therefore avoid hospitalization/death. In turn, since they no longer suffer hospitalization/death, the model then appears to be poorly predictive upon retrospective review. This might be further discussed.

8. Given that it is stated that pneumonia is the single biggest cause of mortality in children under 5 years of age, the applicability of the random forest model to other datasets would be of interest. It should be highly recommended to validate the model on datasets from other sources/countries (possibly with a reduced set of features), if possible.

9. In Table 1, the denominator for the Death outcome is lower than that for the Hospitalization outcome (and the input features). This might be explained.

10. From Table 2, existing risk score methods had AUC<=0.67 on the test set, compared to AUC=0.87 for the BIOTOPE random forest model. It might be commented as to whether these (relatively low) AUCs by PERCH/PREPARE/RISC/WHO are consistent with existing literature on their performance.

11. In eFigure 1, the study sites are shown. Their status as training or test cohort might also be listed.

12. For the list of input variables in S2, it might be considered to firstly format them as a table (with index numbers), and secondly to include columns for each of the models and subsets (BIOTOPE [x4 subsets from feature selection]/PERCH/PREPARE/RISC/WHO) indicating what features were available/used for each of these models.

Reviewer #4: Summary:

The manuscript describes a study to develop and evaluate a machine learning model to predict hospitalisation and/or death in childhood pneumonia using data from the BIOTOPE cohort study. The training and testing cohorts differ in location, with 9 primary care centres in Northern Malawi used for training and 2 used for testing, and time, with the training data from 2022/23 and testing data from 2016.

Note that I have expertise in applied machine learning, but not pneumonia, so my review predominantly focuses on the modeling aspect of the work.

Importance of the research question:

Pneumonia is the leading cause of death in children under 5 years globally. The authors aimed to develop a tool to improve risk stratification, which has the potential to improve patient outcomes by informing triage, referral and treatment decisions. Such a tool with the potential to improve outcomes for patients with pneumonia is, therefore, of high importance.

Advance over existing knowledge:

Four previous models to predict the similar outcomes are cited and the authors compare their model to these previous models. These previous models use death as the outcome to be predicted and are based on data from hospitalized patients. The BIOTOPE study instead uses data from patients in a primary care setting and has hospitalisation and/or death as the outcome to be predicted. The data from the 2016 cohort of the BIOTOPE study were previously published, but the 2022/23 cohort data have not previously been published. The new data and model, therefore, represent an important advance over these previous studies.

Interest to clinicians and policymakers who are not specialists in this topic:

The application of machine learning to predict patient outcomes and support clinical decisions is a widely used approach across fields of medical research. The study is, therefore, likely to be of interest to clinicians and policymakers developing similar tools in other fields of medicine.

What are the main claims of the paper and how significant are they for the discipline?

The main claim of the paper is that the authors have developed a machine learning model to predict hospitalization and/or death from pneumonia that outperforms previous models. Such a claim, if it holds up upon better measures of model evaluation (see comments below), appears to be significant for the discipline. However, I am not an expert in pneumonia so others would be better placed to judge.

It is unsurprising that the model outperforms previous models as these are trained using data from a different setting and with a different outcome. The fact that other models based on the same setting as used in this study do not exist is a strength of the current work.

There is only 1 death event between the training and testing cohorts. Therefore, the focus of the manuscript should be on hospitalisation, e.g. "death" could be removed from the title.

Are the claims properly placed in the context of the previous literature? Have the authors treated the literature fairly?

A good explanation of how the work fits in the context of previous literature and policy guidelines is given. However, more emphasis on the differences between the settings and outcomes of the different models, which is well articulated in the Discussion section, should be explained in the Introduction.

Do the data and analyses fully support the claims? If not, what other evidence is required?

Clarification of model evaluation procedures together with additional measures of model performance are necessary to judge whether the data and analyses support the claims.

Model probability calibration was not performed. This performance measure is key to understanding uncertainty in the model predictions.

The suitability of the approach for handling missing data requires investigation. Specifically, it is important to consider whether the missing data were the data missing at random or not. Sensitivity analysis should be performed to understand whether the results are robust to the strategy used to handle missing data.

Table 2: Measures of uncertainty, e.g. standard deviation, should be included in addition to mean for model performance metrics.

Is this paper outstanding in its discipline? If yes, what makes it outstanding? If not, why not?

Within the discipline of applied machine learning in medicine the study, in its present form, is not outstanding as the model evaluation procedure does not follow best practice (e.g. by following TRIPOD/TRIPOD-AI guidelines).

The novelty is in the data rather than the machine learning, which makes use of classical methods. Four different classes of machine learning model were trained and evaluated. However, models which have been shown to exhibit excellent performance on tabular data, e.g. XGBoost, were not studied. The application of a tabular foundation model (Hollmann et al. Nature 2025) could be a novel methodological approach to try, but not necessary.

Does the study conform to any relevant guidelines?

The study does not follow the TRIPOD or TRIPOD-AI guidelines, which are highly relevant for the study. The quality of the modelling would be substantially improved by following these guidelines.

Are details of the methodology sufficient to allow the experiments to be reproduced?

It is not clear how the performance evaluation on the training data was done. From the description, it does not appear that nested cross-validation was used for internal validation of the model. If not performed, this internal validation strategy would lead to "data leakage" and overoptimistic estimates of model generalizability. It is important to clarify this point.

Page 6, Study population: It is not clear whether the sites included in the 2016 cohort are a subset of those included in the 2022/23 cohort or different sites. This point should be clarified.

Page 8, Model selection: It is not clear whether the feature selection was performed separately for the training and testing data. If feature selection was performed once on all the data, rather than separately for the training and testing data, there would be "data leakage" resulting in overoptimistic estimates of model generalizability. This point should be clarified.

The names and version numbers of software packages used should be included in the Methods section so that the analysis can be replicated.

Is any software created by the authors freely available?

The code is made freely available on GitHub.

Is the manuscript well organized and written clearly enough to be accessible to non-specialists?

The manuscript is generally well organized, clearly written and accessible to a general medical audience.

Abstract: "background" is spelled incorrectly.

Page 6, Study population: "difficult breathing" be "difficulty breathing"?

Page 7, Data preprocessing: It would be informative to state the number of covariates and a brief description of what kind of data they are, e.g. demographic, symptoms, in the Methods section text as these may not be obvious to a general medical audience. It would also be informative to add a correlation matrix for the covariates and outcome data as a supplementary figure to visualise how they are all related to each other.

Page 8, Model selection: "most optimal" should be "optimal".

Any attachments provided with reviews can be seen via the following link: [LINK]

--------------------------------------------------------- ---

General editorial requests:

(Note: not all will apply to your paper, but please check each item carefully)

* We ask every co-author listed on the manuscript to fill in a contributing author statement, making sure to declare all competing interests. If any of the co-authors have not filled in the statement, we will remind them to do so when the paper is revised. If all statements are not completed in a timely fashion this could hold up the re-review process. If new competing interests are declared later in the revision process, this may also hold up the submission. Should there be a problem getting one of your co-authors to fill in a statement we will be in contact. Please do not add or remove authors without first discussing this with the handling editor. You can see our competing interests policy here: http://journals.plos.org/plosmedicine/s/competing-interests.

* Please upload any figures associated with your paper as individual TIF or EPS files with 300dpi resolution at resubmission; please read our figure guidelines for more information on our requirements: http://journals.plos.org/plosmedicine/s/figures. While revising your submission, please upload your figure files to the PACE digital diagnostic tool, https://pacev2.apexcovantage.com/. PACE helps ensure that figures meet PLOS requirements. To use PACE, you must first register as a user. Then, login and navigate to the UPLOAD tab, where you will find detailed instructions on how to use the tool. If you encounter any issues or have any questions when using PACE, please email us at PLOSMedicine@plos.org.

* Please ensure that the paper adheres to the PLOS Data Availability Policy (see http://journals.plos.org/plosmedicine/s/data-availability), which requires that all data underlying the study's findings be provided in a repository or as Supporting Information. For data residing with a third party, authors are required to provide instructions with contact information (web or email address) for obtaining the data. Please note that a study author cannot be the contact person for the data. PLOS journals do not allow statements supported by "data not shown" or "unpublished results." For such statements, authors must provide supporting data or cite public sources that include it.

* We expect all researchers with submissions to PLOS in which author-generated code underpins the findings in the manuscript to make all author-generated code available without restrictions upon publication of the work. In cases where code is central to the manuscript, we may require the code to be made available as a condition of publication. Authors are responsible for ensuring that the code is reusable and well documented. Please make any custom code available, either as part of your data deposition or as a supplementary file. Please add a sentence to your data availability statement regarding any code used in the study, e.g. "The code used in the analysis is available from Github [URL] and archived in Zenodo [DOI link]" Please review our guidelines at https://journals.plos.org/plosmedicine/s/materials-software-and-code-sharing and ensure that your code is shared in a way that follows best practice and facilitates reproducibility and reuse. Because Github depositions can be readily changed or deleted, we encourage you to make a permanent DOI'd copy (e.g. in Zenodo) and provide the URL.

FORMATTING - GENERAL

* Abstract: Please structure your abstract using the PLOS Medicine headings (Background, Methods and Findings, Conclusions). Please combine the Methods and Findings sections into one section.

* At this stage, we ask that you include a short, non-technical Author Summary of your research to make findings accessible to a wide audience that includes both scientists and non-scientists. The Author Summary should immediately follow the Abstract in your revised manuscript. This text is subject to editorial change and should be distinct from the scientific abstract. Ideally each sub-heading should contain 2-3 single sentence, concise bullet points containing the most salient points from your study. In the final bullet point of 'What Do These Findings Mean?', please include the main limitations of the study in non-technical language. Please see our author guidelines for more information: https://journals.plos.org/plosmedicine/s/revising-your-manuscript#loc-author-summary.

* Please express the main results with 95% CIs as well as p values. When reporting p values please report as p<0.001 and where higher as the exact p value p=0.002, for example. Throughout, suggest reporting statistical information as follows to improve clarity for the reader "22% (95% CI [13%,28%]; p</=)". Please be sure to define all numerical values at first use.

* Please include page numbers and line numbers in the manuscript file. Use continuous line numbers (do not restart the numbering on each page).

* Please cite the reference numbers in square brackets. Citations should precede punctuation.

FIGURES AND TABLES

* Please provide titles and legends for all figures and tables (including those in Supporting Information files).

* Please define all abbreviations used in each figure/table (including those in Supporting Information files).

* Please consider avoiding the use of red and green in order to make your figure more accessible to those with color blindness.

SUPPLEMENTARY MATERIAL

* Please note that supplementary material will be posted as supplied by the authors. Therefore, please amend it according to the relevant comments outlined here.

* Please cite your Supporting Information as outlined here: https://journals.plos.org/plosmedicine/s/supporting-information

REFERENCES

* PLOS uses the numbered citation (citation-sequence) method and first six authors, et al.

* Please ensure that journal name abbreviations match those found in the National Center for Biotechnology Information (NCBI) databases (http://www.ncbi.nlm.nih.gov/nlmcatalog/journals), and are appropriately formatted and capitalised.

* Where website addresses are cited, please include the complete URL and specify the date of access (e.g. [accessed: 12/06/2023]).

* Please also see https://journals.plos.org/plosmedicine/s/submission-guidelines#loc-references for further details on reference formatting.

MODELLING STUDIES

The following list is derived from Geoffrey P Garnett, Simon Cousens, Timothy B Hallett, Richard Steketee, Neff Walker. Mathematical models in the evaluation of health programmes. (2011) Lancet DOI:10.1016/S0140-6736(10)61505-X:

* If pertinent, please provide a diagram that shows the model structure, including how the natural history of the disease is represented, the process and determinants of disease acquisition, and how the putative intervention could affect the system.

* Please provide a complete list of model parameters, including clear and precise descriptions of the meaning of each parameter, together with the values or ranges for each, with justification or the primary source cited and important caveats about the use of these values noted.

* Please provide a clear statement about how the model was fitted to the data, including goodness-of-fit measure, the numerical algorithm used, which parameter varied, constraints imposed on parameter values, and starting conditions.

* For uncertainty analyses, please state the sources of uncertainties quantified and not quantified [can include parameter, data, and model structure].

* Please provide sensitivity analyses to identify which parameter values are most important in the model. Uncertainty estimates seek to derive a range of credible results on the basis of an exploration of the range of reasonable parameter values. The choice of method should be presented and justified.

* Please discuss the scientific rationale for the choice of model structure and identify points where this choice could influence conclusions drawn. Please also describe the strength of the scientific basis underlying the key model assumptions.

* For studies that develop a prediction model or evaluate its performance, please ensure that the study is reported according to the TRIPOD statement (https://www.equator-network.org/reporting-guidelines/tripod-statement) and include the completed checklist as Supporting Information. Please add the following statement, or similar, to the Methods: "This study is reported as per the Transparent Reporting of a Multivariable Prediction Model for Individual Prognosis Or Diagnosis (TRIPOD) statement (S1 Checklist)." For studies using machine learning, please use the TRIPOD-AI checklist. When completing the checklist, please use section and paragraph numbers, rather than page numbers.

# Author comment: Author response to Decision Letter 2

**DOI:** 10.1371/journal.pmed.1005122.r003

15 Oct 2025

Attachment Submitted filename: Response to reviewers.docx

# Peer review: Decision Letter 2

**DOI:** 10.1371/journal.pmed.1005122.r004

**Authors:** Andreia Cunha

21 Nov 2025

Dear Dr Gallagher,

Many thanks for submitting your revised manuscript "A Machine Learning Algorithm to Predict Hospitalisation and/or Death in Childhood Pneumonia" (PMEDICINE-D-25-01572R2) to PLOS Medicine. The paper has been re-reviewed by subject experts and a statistician; their comments are included below and can also be accessed here: [LINK]

As you will see, the reviewers still maintain some concerns that we must ask you to address. In particular, it is our editorial view that the manuscript should be refocused on the hospitalization outcome, and this should be made clear in the title. After discussing the paper with the editorial team and an academic editor with relevant expertise, I'm pleased to invite you to revise the paper in response to the reviewers' comments. We plan to send the revised paper to some or all of the original reviewers, and we cannot provide any guarantees at this stage regarding publication.

In addition to these revisions, you may need to complete some formatting changes, which you will receive in a follow up email. A member of our team will be in touch with a set of requests shortly. If you do not receive a separate email within a few days, please assume that checks have been completed, and no additional changes are required.

When you upload your revision, please include a point-by-point response that addresses all of the reviewer and editorial points, indicating the changes made in the manuscript and either an excerpt of the revised text or the location (eg: page and line number) where each change can be found. Please also be sure to check the general editorial comments at the end of this letter and include these in your point-by-point response. When you resubmit your paper, please include a clean version of the paper as the main article file and a version with changes tracked as a marked-up manuscript. It may also be helpful to check the guidelines for revised papers at http://journals.plos.org/plosmedicine/s/revising-your-manuscript for any that apply to your paper.

We ask that you submit your revision by Jan 30 2026 11:59PM. However, if this deadline is not feasible, please contact me by email, and we can discuss a suitable alternative.

Don't hesitate to contact me directly with any questions (acunha@plos.org).

Best regards,

Andreia

Andreia Cunha, PhD

Senior editor

PLOS Medicine

acunha@plos.org

-----------------------------------------------------------

Comments from the academic editor:

In terms of response to my comments, I would like to know a little more about the Integrated Community Health Information System (iCHIS). Is something that is being implmeented widely with complete data collection? It wasn't clear what could be expected in terms of everyone having this level of extensive available. There are many detailed data collection efforts though completeness is often a very different story.

-----------------------------------------------------------

Comments from the reviewers:

Reviewer #1: I appreciate that the authors have addressed the previous comments. However, since the dataset includes only one death case, it is not appropriate to claim that the model can predict mortality. I would also recommend revising the manuscript title to "A Machine Learning Algorithm to Predict Hospitalisation in Childhood Pneumonia in Malawi" to better reflect the scope and location of the study.

Reviewer #2: In science, we have an obligation to present our work and findings accurately, ensuring that our readers understand the results and can determine whether they are generalizable to different settings. By clinging to a composite hospitalization/death outcome, despite only 1 death in the entire dataset, the authors have chosen, in my humble opinion, to misrepresent their work. It doesn't matter whether a paragraph in the discussion touches on this point or if this was a pre-specified endpoint. Simply including death as a potential outcome that this tool could predict in the title is misleading at best. This is the primary reason why I urge rejection of this manuscript.

Additional points relavent to why I recommend rejection of this manuscript:

Utilization of the Ministry of Health hospitalization criteria to predict hospitalization is not methodologically sound. Just integrate a checklist for indications for hospital referral into iCHS. Just because a clinician makes a decision to hospitalize a patient or not hospitalize them it does not mean that decision was sound - this is why post-discharge follow up data is crucial and death is an important outcome that needs to be taken into consideration.

Reviewer #3: We thank the authors for addressing our previous comments, and note the planned future validation of the model.

Reviewer #4: I wish to thank the authors for addressing my comments. In my opinion, the manuscript is now improved. I have a few remaining points, which I think are important to address:

I thank the authors for adding the model probability calibration. However, it is not clear to me whether this has been performed on the training data (in which case it should also be performed on the testing data - probability calibration should be included in both the nested cross-validation procedure for internal validation and external validation, just like AUC and AUPRC) or the testing data (in which case the description should be moved to the subsection describing the external validation, and also included as part of the nested cross-validation procedure). Moreover, the authors have only added one sentence to the text to state that probability calibration was performed. They should add a description and a discussion of the calibration result. From the figure it can be seen that there is substantial miscalibration. For the calibration plot, the unity line (y = x) should be added so that the miscalibration can be clearly appreciated. Also, the slope and y-intercept of the logistic regression model should be added to the plot and described in the main text. The probability calibration is arguably the most important measure of the predictive performance of a model. In reality, users of the model for are given a predicted probability of hospitalisation or death and it is important to understand the accuracy of that predicted probability. The model discrimination (as assessed by the AUC) indicates how well the model correctly predicts the outcomes of two individuals that have different outcomes, which is not the scenario that users of the model face. Therefore, it is vital to describe and discuss the probability calibration in more detail. Also, the comparison with the other models should include assessment of the probability calibration.

The authors have responded to my comment about missing data in my previous review. However, I do not think that my point has been adequately addressed in the revised manuscript. From their response to my comment, it seems as though the authors have performed some kind of sensitivity analysis and analysis of whether data are missing at random. However, I cannot see these analyses in the supplement and I cannot see a reference to it in the main text. I strongly suggest including a sensitivity analysis of the method for handling missing data in the supplement and briefly commenting on the results of this analysis, e.g. whether data are missing at random or not and the impact of the strategy for handling missing data on the results, in the main text.

I wish to thank the authors for adding the correlation matrix. I recommend changing the colorscale so that it is diverging and centered at 0, e.g. red (+1), white (0), blue (-1).

In the abstract, the quoted model AUC and AUPRC values are described as "mean" values. The external validation values should be single values rather than means. I suspect that this phrasing is a typo.

In the abstract, the wording used implies that "more dynamic prediction" is facilitated by machine learning. However, this is not the case. A traditional statistical model could just as easily be updated over time. Please rephrase this misleading wording.

In the introduction on page 5, the authors describe using machine learning and artificial intelligence techniques. However, these are not two distinct approaches employed in the current study. Therefore, I suggest just using "machine learning" (or clarify what the difference is in the context of this study).

Any attachments provided with reviews can be seen via the following link: [LINK]

--------------------------------------------------------- ---

General editorial requests:

(Note: not all will apply to your paper, but please check each item carefully)

* We ask every co-author listed on the manuscript to fill in a contributing author statement, making sure to declare all competing interests. If any of the co-authors have not filled in the statement, we will remind them to do so when the paper is revised. If all statements are not completed in a timely fashion this could hold up the re-review process. If new competing interests are declared later in the revision process, this may also hold up the submission. Should there be a problem getting one of your co-authors to fill in a statement we will be in contact. Please do not add or remove authors without first discussing this with the handling editor. You can see our competing interests policy here: http://journals.plos.org/plosmedicine/s/competing-interests.

* Please upload any figures associated with your paper as individual TIF or EPS files with 300dpi resolution at resubmission; please read our figure guidelines for more information on our requirements: http://journals.plos.org/plosmedicine/s/figures. While revising your submission, we strongly recommend that you use PLOS's NAAS tool (https://ngplosjournals.pagemajik.ai/artanalysis) to test your figure files. NAAS can convert your figure files to the TIFF file type and meet basic requirements (such as print size, resolution), or provide you with a report on issues that do not meet our requirements and that NAAS cannot fix.

After uploading your figures to PLOS's NAAS tool - https://ngplosjournals.pagemajik.ai/artanalysis, NAAS will process the files provided and display the results in the "Uploaded Files" section of the page as the processing is complete.

If the uploaded figures meet our requirements (or NAAS is able to fix the files to meet our requirements), the figure will be marked as "fixed" above. If NAAS is unable to fix the files, a red "failed" label will appear above.

When NAAS has confirmed that the figure files meet our requirements, please download the file via the download option, and include these NAAS processed figure files when submitting your revised manuscript.

* Please ensure that the paper adheres to the PLOS Data Availability Policy (see http://journals.plos.org/plosmedicine/s/data-availability), which requires that all data underlying the study's findings be provided in a repository or as Supporting Information. For data residing with a third party, authors are required to provide instructions with contact information (web or email address) for obtaining the data. Please note that a study author cannot be the contact person for the data. PLOS journals do not allow statements supported by "data not shown" or "unpublished results." For such statements, authors must provide supporting data or cite public sources that include it.

* We expect all researchers with submissions to PLOS in which author-generated code underpins the findings in the manuscript to make all author-generated code available without restrictions upon publication of the work. In cases where code is central to the manuscript, we may require the code to be made available as a condition of publication. Authors are responsible for ensuring that the code is reusable and well documented. Please make any custom code available, either as part of your data deposition or as a supplementary file. Please add a sentence to your data availability statement regarding any code used in the study, e.g. "The code used in the analysis is available from Github [URL] and archived in Zenodo [DOI link]" Please review our guidelines at https://journals.plos.org/plosmedicine/s/materials-software-and-code-sharing and ensure that your code is shared in a way that follows best practice and facilitates reproducibility and reuse. Because Github depositions can be readily changed or deleted, we encourage you to make a permanent DOI'd copy (e.g. in Zenodo) and provide the URL.

FORMATTING - GENERAL

* Abstract: Please structure your abstract using the PLOS Medicine headings (Background, Methods and Findings, Conclusions). Please combine the Methods and Findings sections into one section.

* At this stage, we ask that you include a short, non-technical Author Summary of your research to make findings accessible to a wide audience that includes both scientists and non-scientists. The Author Summary should immediately follow the Abstract in your revised manuscript. This text is subject to editorial change and should be distinct from the scientific abstract. Ideally each sub-heading should contain 2-3 single sentence, concise bullet points containing the most salient points from your study. In the final bullet point of 'What Do These Findings Mean?', please include the main limitations of the study in non-technical language. Please see our author guidelines for more information: https://journals.plos.org/plosmedicine/s/revising-your-manuscript#loc-author-summary.

* Please express the main results with 95% CIs as well as p values. When reporting p values please report as p<0.001 and where higher as the exact p value p=0.002, for example. Throughout, suggest reporting statistical information as follows to improve clarity for the reader "22% (95% CI [13%,28%]; p</=)". Please be sure to define all numerical values at first use.

* Please include page numbers and line numbers in the manuscript file. Use continuous line numbers (do not restart the numbering on each page).

* Please cite the reference numbers in square brackets. Citations should precede punctuation.

FIGURES AND TABLES

* Please provide titles and legends for all figures and tables (including those in Supporting Information files).

* Please define all abbreviations used in each figure/table (including those in Supporting Information files).

* Please consider avoiding the use of red and green in order to make your figure more accessible to those with color blindness.

SUPPLEMENTARY MATERIAL

* Please note that supplementary material will be posted as supplied by the authors. Therefore, please amend it according to the relevant comments outlined here.

* Please cite your Supporting Information as outlined here: https://journals.plos.org/plosmedicine/s/supporting-information

REFERENCES

* PLOS uses the numbered citation (citation-sequence) method and first six authors, et al.

* Please ensure that journal name abbreviations match those found in the National Center for Biotechnology Information (NCBI) databases (http://www.ncbi.nlm.nih.gov/nlmcatalog/journals), and are appropriately formatted and capitalised.

* Where website addresses are cited, please include the complete URL and specify the date of access (e.g. [accessed: 12/06/2023]).

* Please also see https://journals.plos.org/plosmedicine/s/submission-guidelines#loc-references for further details on reference formatting.

MODELLING STUDIES

The following list is derived from Geoffrey P Garnett, Simon Cousens, Timothy B Hallett, Richard Steketee, Neff Walker. Mathematical models in the evaluation of health programmes. (2011) Lancet DOI:10.1016/S0140-6736(10)61505-X:

* If pertinent, please provide a diagram that shows the model structure, including how the natural history of the disease is represented, the process and determinants of disease acquisition, and how the putative intervention could affect the system.

* Please provide a complete list of model parameters, including clear and precise descriptions of the meaning of each parameter, together with the values or ranges for each, with justification or the primary source cited and important caveats about the use of these values noted.

* Please provide a clear statement about how the model was fitted to the data, including goodness-of-fit measure, the numerical algorithm used, which parameter varied, constraints imposed on parameter values, and starting conditions.

* For uncertainty analyses, please state the sources of uncertainties quantified and not quantified [can include parameter, data, and model structure].

* Please provide sensitivity analyses to identify which parameter values are most important in the model. Uncertainty estimates seek to derive a range of credible results on the basis of an exploration of the range of reasonable parameter values. The choice of method should be presented and justified.

* Please discuss the scientific rationale for the choice of model structure and identify points where this choice could influence conclusions drawn. Please also describe the strength of the scientific basis underlying the key model assumptions.

* For studies that develop a prediction model or evaluate its performance, please ensure that the study is reported according to the TRIPOD statement (https://www.equator-network.org/reporting-guidelines/tripod-statement) and include the completed checklist as Supporting Information. Please add the following statement, or similar, to the Methods: "This study is reported as per the Transparent Reporting of a Multivariable Prediction Model for Individual Prognosis Or Diagnosis (TRIPOD) statement (S1 Checklist)." For studies using machine learning, please use the TRIPOD-AI checklist. When completing the checklist, please use section and paragraph numbers, rather than page numbers.

# Author comment: Author response to Decision Letter 3

**DOI:** 10.1371/journal.pmed.1005122.r005

2 Feb 2026

Attachment Submitted filename: Response to Reviewers (1).docx

# Peer review: Decision Letter 3

**DOI:** 10.1371/journal.pmed.1005122.r006

**Authors:** Andreia Cunha

10 Mar 2026

Dear Dr Gallagher,

Many thanks for submitting your manuscript "A Machine Learning Algorithm to Predict Hospitalisation in Childhood Pneumonia" (PMEDICINE-D-25-01572R3) to PLOS Medicine. The paper has been reviewed one of the original subject experts and by the academic editor who reviewed your response to Reviewer 2; their comments are included below and can also be accessed here: [LINK]

As you will see, there are still some important concerns that we must ask you to address in full. After discussing the paper with the editorial team and an academic editor with relevant expertise, I'm pleased to invite you to revise the paper in response to the reviewers' comments. We plan to send the revised paper to some of the original reviewers, and we cannot provide any guarantees at this stage regarding publication.

In addition to these revisions, you may need to complete some formatting changes, which you will receive in a follow up email. A member of our team will be in touch with a set of requests shortly. If you do not receive a separate email within a few days, please assume that checks have been completed, and no additional changes are required.

When you upload your revision, please include a point-by-point response that addresses all of the reviewer and editorial points, indicating the changes made in the manuscript and either an excerpt of the revised text or the location (eg: page and line number) where each change can be found. Please also be sure to check the general editorial comments at the end of this letter and include these in your point-by-point response. When you resubmit your paper, please include a clean version of the paper as the main article file and a version with changes tracked as a marked-up manuscript. It may also be helpful to check the guidelines for revised papers at http://journals.plos.org/plosmedicine/s/revising-your-manuscript for any that apply to your paper.

We ask that you submit your revision by Mar 31 2026 11:59PM. However, if this deadline is not feasible, please contact me by email, and we can discuss a suitable alternative.

Don't hesitate to contact me directly with any questions (acunha@plos.org).

Best regards,

Andreia

Andreia Cunha, PhD

Senior editor

PLOS Medicine

acunha@plos.org

-----------------------------------------------------------

Comments from the academic editor:

I think with regards to the composite outcome, their use of it is appropriate in that it capture all children at risk for a bad outcome (which certainly includes death). I think the main issue was the initial framing of describing the models ability to predict hospitalization/death when really much can't be said about death (even though it was initially pre-specficied as an outcome).

Specific Thoughts:

Removing it from the title is good.

In the Methods, they could specify there that there was only 1 death in their dataset rather than just say low-mortality.

I think abstract should also make this type of clarification somewhere as well. Could be a limitation at end of abstract.

In some ways, a good description of their outcome is children at risk of poor outcomes but okay to stay with hospitalization too (just another way of describing the meaning of the outcome).

With regards to R2 other comment, death and/or objective deterioration would certainly be better outcomes to use if feasible. But in this circumstance where it isn't feasible, I do still think hospitalization is a reasonable outcome. If there is a more robust objective "indications for hospitalizations" that go beyond WHO Danger Signs that could be specified as an alternative outcome, it would strengthen the paper to demonstrate the models performance against that as well.

-----------------------------------------------------------

Comments from the reviewers:

Reviewer #4: I wish to thank the authors for addressing my comments. In my opinion, the manuscript is now improved. In particular, the probability calibration is now well described in the Methods section and my comments about the sensitivity analysis and minor comments have been addressed. However, the interpretation and discussion of the substantial miscalibration observed in the external validation are insufficient. The following improvements to the probability calibration would further strengthen this aspect of the work:

1) Most of the references to probability calibration focus on expected calibration error. The calibration intercept and slope are more informative measures of probability calibration than expected calibration error, which does not provide information on whether the predicted probabilities are under- or overestimates, and depends on binning. Therefore, calibration intercept and slope should be reported in addition to the expected calibration error and these values should be reported in the main text wherever AUC and PRAUC are described instead of, or in addition to, the expected calibration error.

2) The Discussion should include a detailed interpretation of the probability calibration results and these findings should be referenced in the Conclusion and Abstract. The model appears to have very poor probability calibration when tested on the external dataset (intercept = -9.76, slope = 3.75). It is vital that readers are aware of this substantial limitation when trying to decide if and how they should use the model. This discussion could also mention recalibrating the model for different settings.

3) eFigure 3, showing the probability calibration results, does not appear to be referenced anywhere in the main text. A reference to this figure should be added to clarify which part of the text relates to it. It is not clear to me how the logistic regression model described in the Methods and probability calibration slope and intercept quoted in the main text relate to eFigure 3. Also, this figure appears to show different results from the previous version of the manuscript (although I could be wrong about this point). These points should be clarified. The figure caption should describe what the figure in more detail, e.g. what does the orange line represent?

4) Table 2, eTable 6 and eTable 7 and Figure 2, eFigure 2, eFigure 4, eFigure 7 and eFigure 8 do not include the probability calibration performance results. Probability calibration results are complementary to AUC and PRAUC and so should be added to assess model performance anywhere AUC and PRAUC have been used.

5) In the new text under "Model selection" in the Methods section, "logistic model" should be "logistic regression model".

Any attachments provided with reviews can be seen via the following link: [LINK]

--------------------------------------------------------- ---

General editorial requests:

(Note: not all will apply to your paper, but please check each item carefully)

* We ask every co-author listed on the manuscript to fill in a contributing author statement, making sure to declare all competing interests. If any of the co-authors have not filled in the statement, we will remind them to do so when the paper is revised. If all statements are not completed in a timely fashion this could hold up the re-review process. If new competing interests are declared later in the revision process, this may also hold up the submission. Should there be a problem getting one of your co-authors to fill in a statement we will be in contact. Please do not add or remove authors without first discussing this with the handling editor. You can see our competing interests policy here: http://journals.plos.org/plosmedicine/s/competing-interests.

* Please upload any figures associated with your paper as individual TIF or EPS files with 300dpi resolution at resubmission; please read our figure guidelines for more information on our requirements: http://journals.plos.org/plosmedicine/s/figures. While revising your submission, we strongly recommend that you use PLOS's NAAS tool (https://ngplosjournals.pagemajik.ai/artanalysis) to test your figure files. NAAS can convert your figure files to the TIFF file type and meet basic requirements (such as print size, resolution), or provide you with a report on issues that do not meet our requirements and that NAAS cannot fix.

After uploading your figures to PLOS's NAAS tool - https://ngplosjournals.pagemajik.ai/artanalysis, NAAS will process the files provided and display the results in the "Uploaded Files" section of the page as the processing is complete.

If the uploaded figures meet our requirements (or NAAS is able to fix the files to meet our requirements), the figure will be marked as "fixed" above. If NAAS is unable to fix the files, a red "failed" label will appear above.

When NAAS has confirmed that the figure files meet our requirements, please download the file via the download option, and include these NAAS processed figure files when submitting your revised manuscript.

* Please ensure that the paper adheres to the PLOS Data Availability Policy (see http://journals.plos.org/plosmedicine/s/data-availability), which requires that all data underlying the study's findings be provided in a repository or as Supporting Information. For data residing with a third party, authors are required to provide instructions with contact information (web or email address) for obtaining the data. Please note that a study author cannot be the contact person for the data. PLOS journals do not allow statements supported by "data not shown" or "unpublished results." For such statements, authors must provide supporting data or cite public sources that include it.

* We expect all researchers with submissions to PLOS in which author-generated code underpins the findings in the manuscript to make all author-generated code available without restrictions upon publication of the work. In cases where code is central to the manuscript, we may require the code to be made available as a condition of publication. Authors are responsible for ensuring that the code is reusable and well documented. Please make any custom code available, either as part of your data deposition or as a supplementary file. Please add a sentence to your data availability statement regarding any code used in the study, e.g. "The code used in the analysis is available from Github [URL] and archived in Zenodo [DOI link]" Please review our guidelines at https://journals.plos.org/plosmedicine/s/materials-software-and-code-sharing and ensure that your code is shared in a way that follows best practice and facilitates reproducibility and reuse. Because Github depositions can be readily changed or deleted, we encourage you to make a permanent DOI'd copy (e.g. in Zenodo) and provide the URL.

FORMATTING - GENERAL

* Abstract: Please structure your abstract using the PLOS Medicine headings (Background, Methods and Findings, Conclusions). Please combine the Methods and Findings sections into one section.

* At this stage, we ask that you include a short, non-technical Author Summary of your research to make findings accessible to a wide audience that includes both scientists and non-scientists. The Author Summary should immediately follow the Abstract in your revised manuscript. This text is subject to editorial change and should be distinct from the scientific abstract. Ideally each sub-heading should contain 2-3 single sentence, concise bullet points containing the most salient points from your study. In the final bullet point of 'What Do These Findings Mean?', please include the main limitations of the study in non-technical language. Please see our author guidelines for more information: https://journals.plos.org/plosmedicine/s/revising-your-manuscript#loc-author-summary.

* Please express the main results with 95% CIs as well as p values. When reporting p values please report as p<0.001 and where higher as the exact p value p=0.002, for example. Throughout, suggest reporting statistical information as follows to improve clarity for the reader "22% (95% CI [13%,28%]; p</=)". Please be sure to define all numerical values at first use.

* Please include page numbers and line numbers in the manuscript file. Use continuous line numbers (do not restart the numbering on each page).

* Please cite the reference numbers in square brackets. Citations should precede punctuation.

FIGURES AND TABLES

* Please provide titles and legends for all figures and tables (including those in Supporting Information files).

* Please define all abbreviations used in each figure/table (including those in Supporting Information files).

* Please consider avoiding the use of red and green in order to make your figure more accessible to those with color blindness.

SUPPLEMENTARY MATERIAL

* Please note that supplementary material will be posted as supplied by the authors. Therefore, please amend it according to the relevant comments outlined here.

* Please cite your Supporting Information as outlined here: https://journals.plos.org/plosmedicine/s/supporting-information

REFERENCES

* PLOS uses the numbered citation (citation-sequence) method and first six authors, et al.

* Please ensure that journal name abbreviations match those found in the National Center for Biotechnology Information (NCBI) databases (http://www.ncbi.nlm.nih.gov/nlmcatalog/journals), and are appropriately formatted and capitalised.

* Where website addresses are cited, please include the complete URL and specify the date of access (e.g. [accessed: 12/06/2023]).

* Please also see https://journals.plos.org/plosmedicine/s/submission-guidelines#loc-references for further details on reference formatting.

# Author comment: Author response to Decision Letter 4

**DOI:** 10.1371/journal.pmed.1005122.r007

24 Mar 2026

Attachment Submitted filename: Reviewer response3.docx

# Peer review: Decision Letter 4

**DOI:** 10.1371/journal.pmed.1005122.r008

**Authors:** Andreia Cunha

16 Apr 2026

Dear Dr. Gallagher,

Sincere apologies for the delay in getting back to you with a decision. Thank you very much for re-submitting your manuscript "A Machine Learning Algorithm to Predict Hospitalisation in Childhood Pneumonia" (PMEDICINE-D-25-01572R4) for review by PLOS Medicine.

I have discussed the paper with my colleagues and the academic editor and it was also seen again by one of the reviewers. I am pleased to say that provided the remaining reviewer, editorial and production issues are dealt with we are planning to accept the paper for publication in the journal.

The remaining issues that need to be addressed are listed at the end of this email. Any accompanying reviewer attachments can be seen via the link below. Please take these into account before resubmitting your manuscript:

[LINK]

***Please note while forming your response, if your article is accepted, you may have the opportunity to make the peer review history publicly available. The record will include editor decision letters (with reviews) and your responses to reviewer comments. If eligible, we will contact you to opt in or out.***

In revising the manuscript for further consideration here, please ensure you address the specific points made by each reviewer and the editors. In your rebuttal letter you should indicate your response to the reviewers' and editors' comments and the changes you have made in the manuscript. Please submit a clean version of the paper as the main article file. A version with changes marked must also be uploaded as a marked up manuscript file.

Please also check the guidelines for revised papers at http://journals.plos.org/plosmedicine/s/revising-your-manuscript for any that apply to your paper. If you haven't already, we ask that you provide a short, non-technical Author Summary of your research to make findings accessible to a wide audience that includes both scientists and non-scientists. The Author Summary should immediately follow the Abstract in your revised manuscript. This text is subject to editorial change and should be distinct from the scientific abstract.

In addition to these revisions, you may need to complete some formatting changes, which you will receive in a follow up email. A member of our team will be in touch with a set of requests shortly. If you do not receive a separate email within a few days, please assume that checks have been completed, and no additional changes are required.

We expect to receive your revised manuscript within 1 week. Please email us (plosmedicine@plos.org) if you have any questions or concerns.

We ask every co-author listed on the manuscript to fill in a contributing author statement. If any of the co-authors have not filled in the statement, we will remind them to do so when the paper is revised. If all statements are not completed in a timely fashion this could hold up the re-review process. Should there be a problem getting one of your co-authors to fill in a statement we will be in contact. YOU MUST NOT ADD OR REMOVE AUTHORS UNLESS YOU HAVE ALERTED THE EDITOR HANDLING THE MANUSCRIPT TO THE CHANGE AND THEY SPECIFICALLY HAVE AGREED TO IT.

Please ensure that the paper adheres to the PLOS Data Availability Policy (see http://journals.plos.org/plosmedicine/s/data-availability), which requires that all data underlying the study's findings be provided in a repository or as Supporting Information. For data residing with a third party, authors are required to provide instructions with contact information for obtaining the data. PLOS journals do not allow statements supported by "data not shown" or "unpublished results." For such statements, authors must provide supporting data or cite public sources that include it.

To enhance the reproducibility of your results, we recommend that you deposit your laboratory protocols in protocols.io, where a protocol can be assigned its own identifier (DOI) such that it can be cited independently in the future. Additionally, PLOS ONE offers an option to publish peer-reviewed clinical study protocols. Read more information on sharing protocols at https://plos.org/protocols?utm_medium=editorial-email&utm_source=authorletters&utm_campaign=protocols

Please review your reference list to ensure that it is complete and correct. If you have cited papers that have been retracted, please include the rationale for doing so in the manuscript text, or remove these references and replace them with relevant current references. Any changes to the reference list should be mentioned in the rebuttal letter that accompanies your revised manuscript.

Please note, when your manuscript is accepted, an uncorrected proof of your manuscript will be published online ahead of the final version, unless you've already opted out via the online submission form. If, for any reason, you do not want an earlier version of your manuscript published online or are unsure if you have already indicated as such, please let the journal staff know immediately at plosmedicine@plos.org.

If you have any questions in the meantime, please contact me or the journal staff on plosmedicine@plos.org.

We look forward to receiving the revised manuscript by Apr 23 2026 11:59PM.

Sincerely,

Andreia Cunha, PhD

Senior Editor

PLOS Medicine

plosmedicine.org

------------------------------------------------------------

Requests from Editors:

GENERAL EDITORIAL REQUESTS

* At this stage, we ask that you include a short, non-technical Author Summary of your research to make findings accessible to a wide audience that includes both scientists and non-scientists. The Author Summary should immediately follow the Abstract in your revised manuscript. This text is subject to editorial change and should be distinct from the scientific abstract. Ideally each sub-heading should contain 2-3 single sentence, concise bullet points containing the most salient points from your study. In the final bullet point of ‘What Do These Findings Mean?’ Please include the main limitations of the study in non-technical language.

Please see our author guidelines for more information: https://journals.plos.org/plosmedicine/s/revising-your-manuscript#loc-author-summary.

* Please confirm that your title complies with PLOS Medicine's style. Your title must be nondeclarative and not a question. It should begin with main concept if possible. "Effect of" should be used only if causality can be inferred, i.e., for an RCT. Please place the study design ("A randomized controlled trial," "A retrospective study," "A modelling study," etc.) in the subtitle (ie, after a colon). A possible suggestion: Prediction of hospitalisation in young children with pneumonia in Malawi: A machine learning–based approach.

* Please confirm that your abstract complies with our requirements, including format (three sections: Background, Methods and Findings, and Conclusions) and providing all the information relevant to this study type https://journals.plos.org/plosmedicine/s/submission-guidelines#loc-abstract . Please Remove "and relevance" from the Conclusions subheading.

* Please ensure that the Introduction ends with a clear description of the study question or hypothesis.

* Please ensure that all abbreviations are defined at first use throughout the text.

* Please confirm that all numbers presented in the abstract are present and identical to numbers presented in the main manuscript text.

GENERAL

* Please review your text for claims of novelty or primacy (e.g. 'for the first time') and remove this language. In addition, please check that any use of statistical terms (such as trend or significant) are supported by the data, and if not please remove them.

* Please remove the 'conclusions' subheading from the discussion. Please also remove any other subheadings from the discussion.

* Statistical reporting: Please revise throughout the manuscript, including tables and figures.

- Please report statistical information as follows to improve clarity for the reader ""22% (95% CI [13,28]; p</=)"".

- Please separate upper and lower bounds with commas instead of hyphens as the latter can be confused with reporting of negative values.

- Please repeat statistical definitions (HR, CI etc.) for each set of parentheses."

* In the last sentence of the Abstract Methods and Findings section, please describe the main limitation(s) of the study's methodology.

* In the author summary, please revise formatting and ensure you use bullet points.

FUNDING STATEMENT

* The funding statement should include: specific grant numbers, initials of authors who received each award, URLs to sponsors’ websites. Also, please state whether any sponsors or funders (other than the named authors) played any role in study design, data collection and analysis, the decision to publish, or preparation of the manuscript. If they had no role in the research, include this sentence: “The funders had no role in study design, data collection and analysis, decision to publish, or preparation of the manuscript.”

COMPETING INTERESTS STATEMENT

* All authors must declare their relevant competing interests per the PLOS policy, which can be seen here: https://journals.plos.org/plosmedicine/s/competing-interests For authors with ties to industry, please indicate whether any of the interests has a financial stake in the results of the current study.

DATA AVAILABILITY

* PLOS Medicine requires that the de-identified data underlying the specific results in a published article be made available, without restrictions on access, in a public repository or as Supporting Information at the time of article publication, provided it is legal and ethical to do so. Please see the policy at

http://journals.plos.org/plosmedicine/s/data-availability

and FAQs at

http://journals.plos.org/plosmedicine/s/data-availability#loc-faqs-for-data-policy

* PLOS defines the “minimal data set” to consist of the data set used to reach the conclusions drawn in the manuscript with related metadata and methods, and any additional data required to replicate the reported study findings in their entirety. Authors do not need to submit their entire data set, or the raw data collected during an investigation. Please submit the following data:

The values behind the means, standard deviations and other measures reported;

The values used to build graphs;

The points extracted from images for analysis.

* The Data Availability Statement (DAS) requires revision. For each data source used in your study:

a) If the data are freely or publicly available, note this and state the location of the data: within the paper, in Supporting Information files, or in a public repository (include the DOI or accession number).

b) If the data are owned by a third party but freely available upon request, please note this and state the owner of the data set and contact information for data requests (web or email address). Note that a study author cannot be the contact person for the data.

c) If the data are not freely available, please describe briefly the ethical, legal, or contractual restriction that prevents you from sharing it. Please also include an appropriate contact (web or email address) for inquiries (again, this cannot be a study author).

* For studies in which a novel model is central to the manuscript's findings, as is the case here, authors are responsible for providing the source code needed to replicate the study's findings in a repository (such as GitHub, SourceForge or Bitbucket) or a cloud computing service (such as Code Ocean). Protection of authors’ intellectual property will not be cause for exception. Please explain in the manuscript’s Data Availability Statement how readers can access the shared code.

ETHICS AND CONSENT

* Please provide the name(s) of the institutional review board(s) that provided ethical approval and include the approval number.

* Please also Indicate the form of consent obtained (written/oral). Please ensure that the research complies with the PLOS policy in full: https://journals.plos.org/plosmedicine/s/human-subjects-research#loc-patient-privacy-and-informed-consent-for-publication

FIGURES

* Figures cannot be reproduced from other sources that are not CC-BY. Please provide a different figure.

* Please provide titles and legends for all figures and tables (including those in Supporting Information files). Please define all acronyms used in each figure or table in its corresponding legend.

INCLUSIVITY IN GLOBAL RESEARCH

PLOS has an 'Inclusivity in Global Research' policy which aims to promote collaboration and inclusivity in global health research. You are required to complete PLOS’ questionnaire on inclusivity in global research and submit it with your revised paper. The policy and questionnaire can be found at https://journals.plos.org/plosone/s/best-practices-in-research-reporting.

MODELLING STUDIES

The following list is derived from Geoffrey P Garnett, Simon Cousens, Timothy B Hallett, Richard Steketee, Neff Walker. Mathematical models in the evaluation of health programmes. (2011) Lancet DOI:10.1016/S0140-6736(10)61505-X:

* If pertinent, please provide a diagram that shows the model structure, including how the natural history of the disease is represented, the process and determinants of disease acquisition, and how the putative intervention could affect the system.

* Please provide a complete list of model parameters, including clear and precise descriptions of the meaning of each parameter, together with the values or ranges for each, with justification or the primary source cited and important caveats about the use of these values noted.

* Please provide a clear statement about how the model was fitted to the data, including goodness-of-fit measure, the numerical algorithm used, which parameter varied, constraints imposed on parameter values, and starting conditions.

* For uncertainty analyses, please state the sources of uncertainties quantified and not quantified [can include parameter, data, and model structure].

* Please provide sensitivity analyses to identify which parameter values are most important in the model. Uncertainty estimates seek to derive a range of credible results on the basis of an exploration of the range of reasonable parameter values. The choice of method should be presented and justified.

* Please discuss the scientific rationale for the choice of model structure and identify points where this choice could influence conclusions drawn. Please also describe the strength of the scientific basis underlying the key model assumptions.

* For studies that develop a prediction model or evaluate its performance, please ensure that the study is reported according to the TRIPOD statement (https://www.equator-network.org/reporting-guidelines/tripod-statement) and include the completed checklist as Supporting Information. Please add the following statement, or similar, to the Methods: "This study is reported as per the Transparent Reporting of a Multivariable Prediction Model for Individual Prognosis Or Diagnosis (TRIPOD) statement (S1 Checklist)." For studies using machine learning, please use the TRIPOD-AI checklist. When completing the checklist, please use section and paragraph numbers, rather than page numbers.

Comments from Reviewers:

Reviewer #4: I thank the authors for addressing my previous comments. In my opinion, the manuscript is now very close to being suitable for publication. The authors have satisfactorily addressed my previous points 1, 3 and 5. I have two final minor comments regarding my previous points 2 and 4, after which I would consider the manuscript suitable for publication.

Regarding point 2, the authors' clarification of the calibration intercept and slope is helpful, and the inclusion of probability calibration metrics improves the manuscript. However, the Discussion still lacks a clear interpretation of these values and their practical implications. In particular, the authors should explicitly explain what an intercept of -0.32 and a slope of 1.13 imply about model reliability (in terms of systematic bias and predictions being too extreme), and whether recalibration would be necessary before use in new settings. Additionally, the statement of "accurate probability calibration" in the Conclusion is not supported by the reported calibration metrics, given that the calibration slope (1.13) and intercept (-0.32) indicate some degree of miscalibration.

Regarding point 4, Table 2 appears to be missing the logistic regression intercept and slope values (for probability calibration) for all models except the BIOTOPE random forest model. These metrics should be included for the remaining models.

Any attachments provided with reviews can be seen via the following link:

[LINK]

# Author comment: Author response to Decision Letter 5

**DOI:** 10.1371/journal.pmed.1005122.r009

16 May 2026

Attachment Submitted filename: Response to editor.docx

# Peer review: Decision Letter 5

**DOI:** 10.1371/journal.pmed.1005122.r010

**Authors:** Andreia Cunha

18 May 2026

Dear Dr Gallagher,

On behalf of my colleagues and the Academic Editor, Dr Aaloke Mody, I am pleased to inform you that we have agreed to publish your manuscript "Prediction of hospitalisation in young children with pneumonia in Malawi: A machine learning–based approach." (PMEDICINE-D-25-01572R5) in PLOS Medicine.

Before your manuscript can be formally accepted you will need to complete some formatting changes, which you will receive in a follow up email. Please be aware that it may take several days for you to receive this email; during this time no action is required by you. Once you have received these formatting requests, please note that your manuscript will not be scheduled for publication until you have made the required changes.

In the meantime, please log into Editorial Manager at http://www.editorialmanager.com/pmedicine/, click the "Update My Information" link at the top of the page, and update your user information to ensure an efficient production process.

PRESS

We frequently collaborate with press offices. If your institution or institutions have a press office, please notify them about your upcoming paper at this point, to enable them to help maximise its impact. If the press office is planning to promote your findings, we would be grateful if they could coordinate with medicinepress@plos.org. If you have not yet opted out of the early version process, we ask that you notify us immediately of any press plans so that we may do so on your behalf.

We also ask that you take this opportunity to read our Embargo Policy regarding the discussion, promotion and media coverage of work that is yet to be published by PLOS. As your manuscript is not yet published, it is bound by the conditions of our Embargo Policy. Please be aware that this policy is in place both to ensure that any press coverage of your article is fully substantiated and to provide a direct link between such coverage and the published work. For full details of our Embargo Policy, please visit http://www.plos.org/about/media-inquiries/embargo-policy/.

To enhance the reproducibility of your results, we recommend that you deposit your laboratory protocols in protocols.io, where a protocol can be assigned its own identifier (DOI) such that it can be cited independently in the future. Additionally, PLOS ONE offers an option to publish peer-reviewed clinical study protocols. Read more information on sharing protocols at https://plos.org/protocols?utm_medium=editorial-email&utm_source=authorletters&utm_campaign=protocols

Thank you again for submitting to PLOS Medicine. We look forward to publishing your paper.

Sincerely,

Andreia Cunha, PhD

Senior Editor

PLOS Medicine
